# Supplementary material for: Structurally diverse macrocycle co-crystals for solid-state luminescence modulation
Source: Nat Commun. 2024 Mar 21;15:2535. doi: 10.1038/s41467-024-46788-6 (PMC10957888; doi:10.1038/s41467-024-46788-6)
Supplement: Supplementary file 1 — Supplementary Information [file 41467_2024_46788_MOESM1_ESM.pdf]

Supplementary Information for

Structurally Diverse Macrocycle Co-Crystals for Solid-State Luminescence Modulation

*Bin Li,[1] Lingling Liu,[1] Yuan Wang,[1] Kun Liu,[1] Zhe Zheng,[1] Shougang Sun,[2] Yongxu Hu,[2] Liqiang Li[2] & Chunju Li[1]**

[1]Academy of Interdisciplinary Studies on Intelligent Molecules, Tianjin Key Laboratory of Structure and Performance for Functional Molecules, College of Chemistry, Tianjin Normal University, Tianjin 300387, P. R. China.

[2]Tianjin Key Laboratory of Molecular Optoelectronic Sciences, Department of Chemistry, Institute of Molecular Aggregation Science, Tianjin University, Tianjin 300072, China.

E-mail: cjli@shu.edu.cn

Contents

1. Supplementary Methods 2
2. Supplementary Discussion 3
   2.1 Synthesis and crystal structure of Pe[3] 3
   2.2 Crystal engineering of CT co-crystals 6
   2.3 Crystallographic data 16
   2.4 Photophysical characterizations 22
   2.5 DFT calculation 30
   2.6 Mechanism of MCCs formation at different stoichiometries 31
3. Supplementary Reference 48

1. Supplementary Methods

TCNB (98%) was purchased from the commercial source without further purification. The preparation of Pe[3] was according to our previous work.[1] Nuclear magnetic resonance (NMR) spectra were recorded on Bruker Avance III 500 MHz. Chemical shifts are reported in ppm relative to the signals corresponding to the residual non-deuterated solvents ($CDCl_3$: δ_H = 7.26 ppm and δ_C = 77.0 ppm). All single crystal X-ray diffraction data were collected on a Bruker D8-Venture or Bruker APEX-II CCD detector using Mo-Kα (λ = 0.71073 Å) or Ga-Kα (λ = 1.34138 Å) radiation. The crystal structure was solved and refined against all F^2 values using the SHELX and Olex 2 suite of programs.[2,3] Fluorescence microscopy images were collected at room temperature on a SOPTOP ICX41 imaging system excited at 375 nm. Solid fluorescence spectra were performed with an Agilent Cary Eclipse spectrophotometer. Solid fluorescence quantum yields were performed on HAMAMATSU C9920-02 by absolute method. Fluorescence lifetimes were measured on FLS1000. The geometries of all the ground state co-crystals were selected from the corresponding X-ray single crystal diffraction data. All calculations were performed using the Gaussian 16 software package.[4] The HOMO/LUMOs of MCCs were calculated with the popular functional B3LYP/6-31G(d, p). The excited state property of Pe-TCNB was calculated at CAM-B3LYP/6-311G* level. Natural transition orbitals (NTOs) were evaluated with the dominant particle-hole pair contributions and the associated transition weights. The orbitals were visualized by VMD program (the isovalue was set as 0.02) assisted with the Multiwfn program.[5]

2. Supplementary Discussion

2.1 Synthesis and crystal structure of Pe[3]

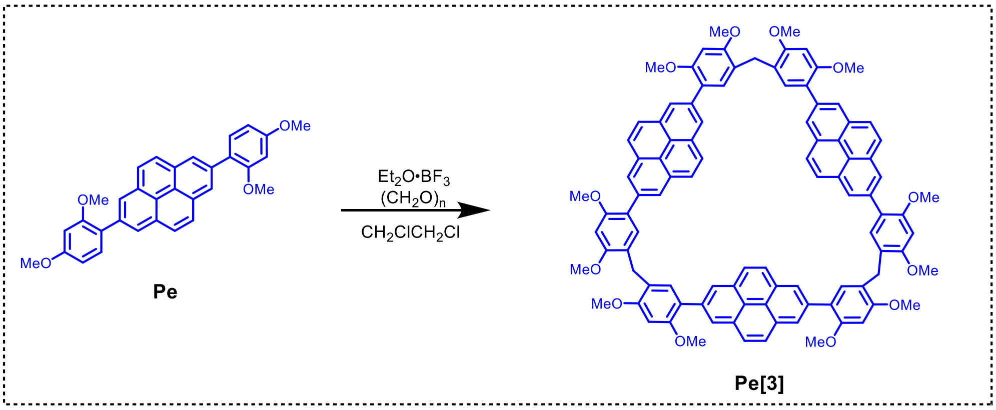

Pe[3]. To the solution of Pe (0.47 g, 1.0 mmol) and paraformaldehyde (0.075 g, 2.5 mmol) in $ClCH_2CH_2Cl$ (200 mL) was added $BF_3 \cdot O_2Et$ (0.010 mL, 0.080 mmol) and stirred at 25 °C for 30 minutes. After quenching by 100 mL saturated $NaHCO_3$ solution, the organic phase was separated and washed with brine and water. The organic layer was evaporated and purified by column chromatography on silica gel (eluent petroleum ether: dichloromethane 5: 1 and gradually changed to petroleum ether: dichloromethane 1: 2.5) to afford white Pe[3] (0.40 g, 81%). ^1H NMR (500 MHz, $CDCl_3$) δ 8.23 (s, 12H), 8.01 (s, 12H), 7.25 (s, 6H), 6.66 (s, 6H), 4.08 (s, 6H), 3.95 (s, 18H), 3.84 (s, 18H); ^{13}C NMR (125 MHz, $CDCl_3$) δ 158.10, 156.08, 136.34, 133.19, 130.98, 127.67, 126.27, 123.54, 123.17, 121.85, 96.35, 56.26, 56.08, 27.69; HRMS (m/z): $[M+H]^+$ calcd. for $C_{99}H_{79}O_{12}^+$, 1460.5600; found, 1460.5604.

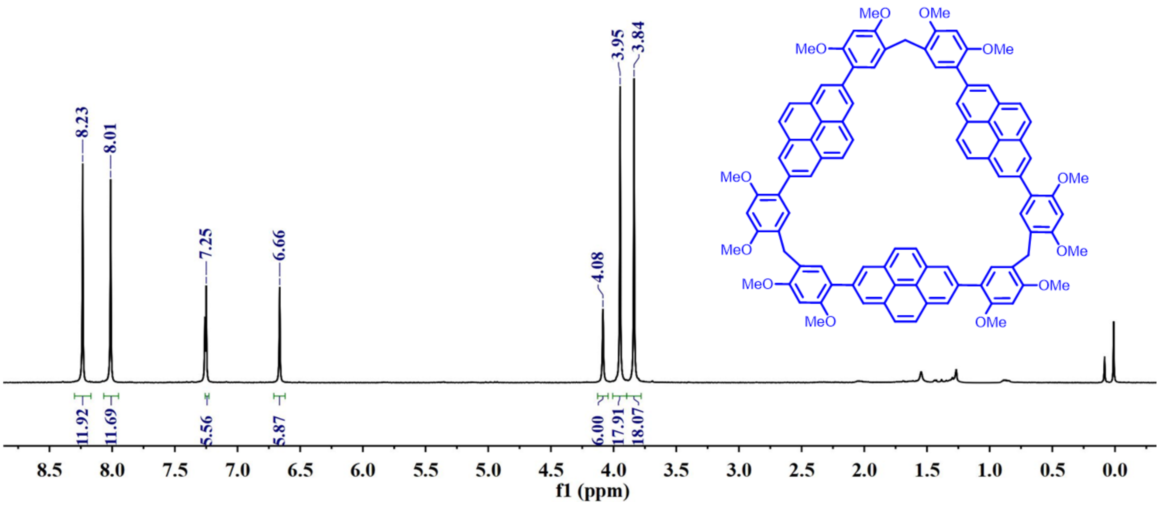

Supplementary Figure 1. ^1H NMR spectrum (500 MHz, $CDCl_3$, 298 K) of Pe[3].

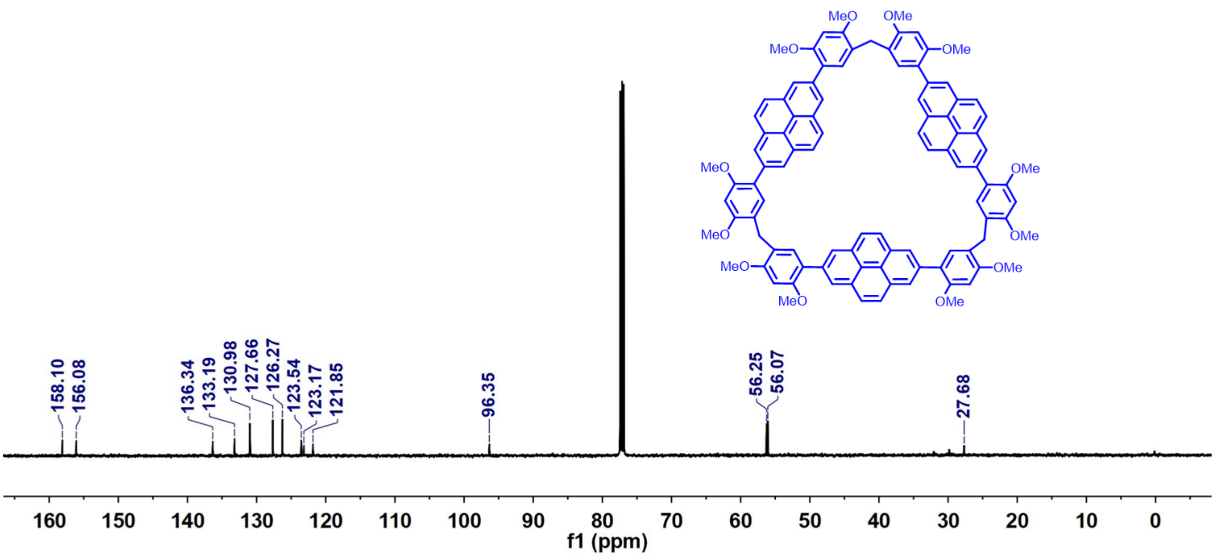

Supplementary Figure 2. ^{13}C NMR spectrum (125 MHz, $CDCl_3$, 298 K) of Pe[3].

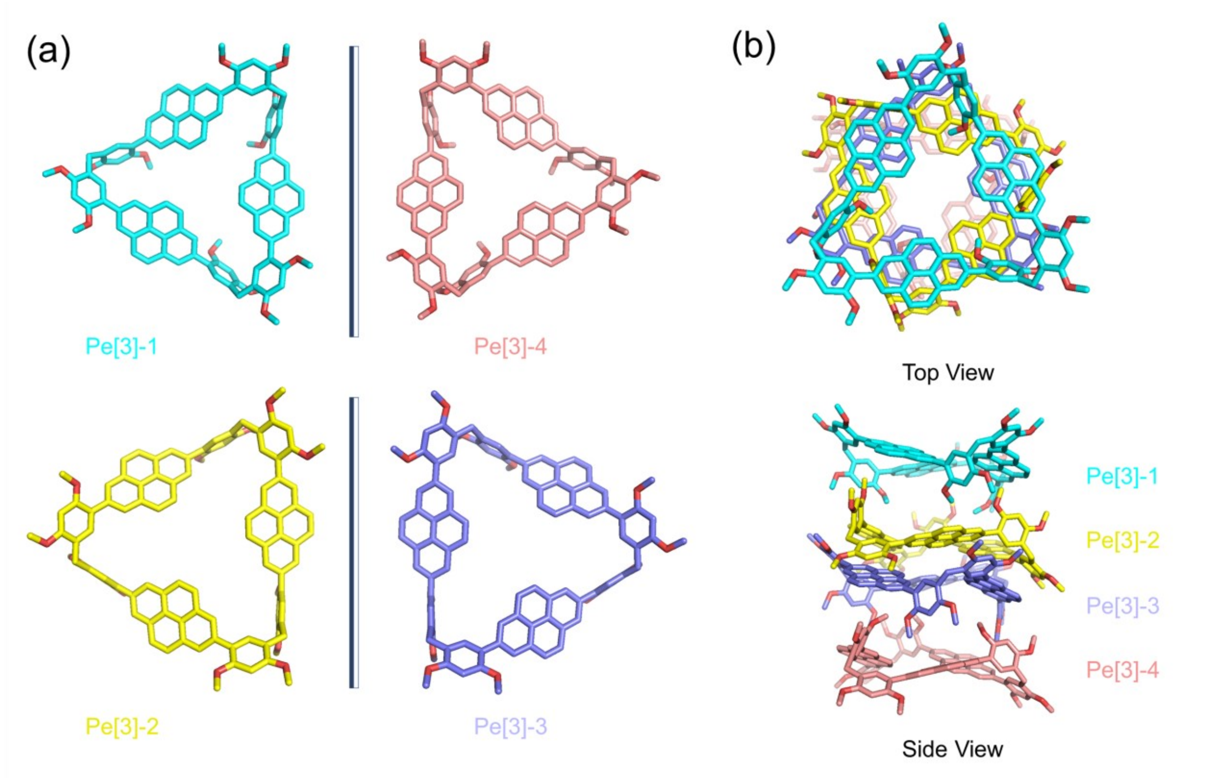

Supplementary Figure 3. (a) Two pairs of enantiomers in the crystal superstructure of Pe[3]. (b) The enantiomers stack in a columnar structure in one cell. Hydrogen atoms are removed for the sake of clarity.

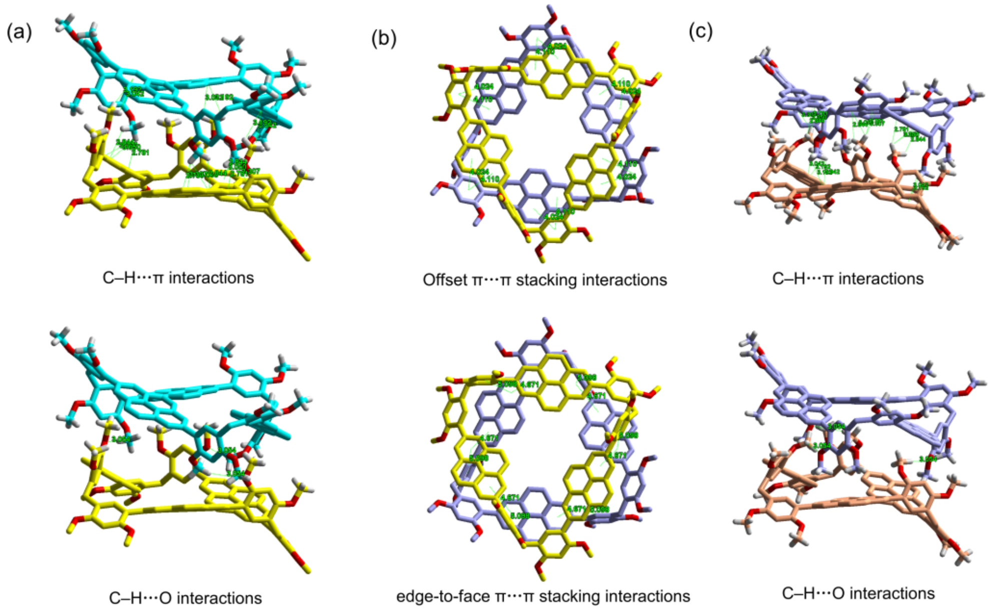

Supplementary Figure 4. (a) Six sets of C–H···π [2.78–3.31 Å] and triple weak C–H···O [3.05 Å] interactions between Pe[3]-1 and Pe[3]-2. (b) Dozen sets of offset π···π interactions [4.02, 4.11 Å] and edge-to-face [4.67, 5.10 Å] π···π interactions between Pe[3]-2 and Pe[3]-3. (c) The interactions between Pe[3]-3 and Pe[3]-4 are the same with Pe[3]-1 and Pe[3]-2.

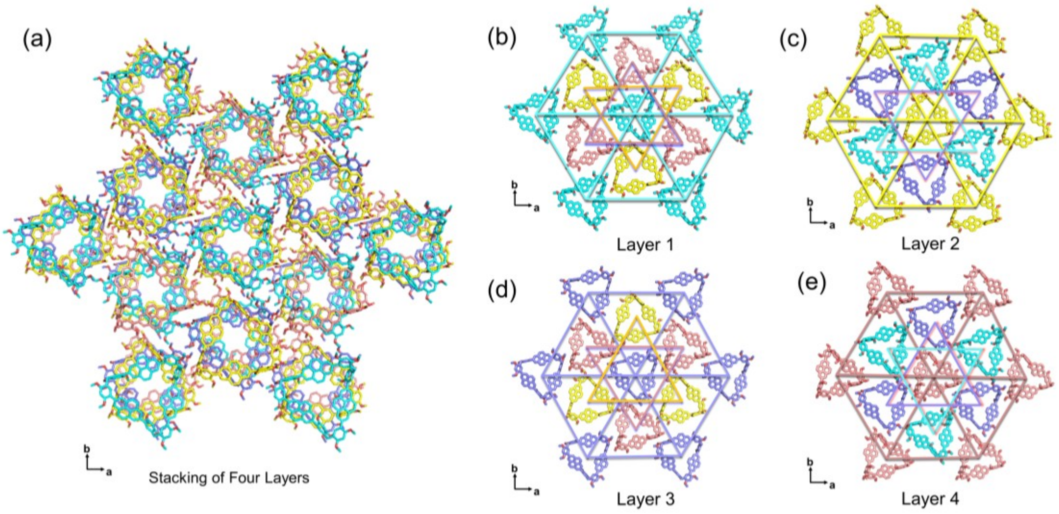

Supplementary Figure 5. (a) Packing of the repeat unit of two pairs of enantiomers in the *ab*-plane form a four-layer honeycomb 2D superstructure. (b–e) Schematic representation of the molecular arrangements of Pe[3] for each layer. Hydrogen atoms are removed for clarity and different colors represent the colors represent the symmetry equivalence.

It can be seen that there is existence of three conformations of macrocycle in each layer. For instance, in the layer 1, seven Pe[3]-1 molecules are arranged in six triangle fashion to form a 1D

regular hexagon in which around Pe[3]-1 can be seen as vertex. Three Pe[3]-2 and Pe[3]-4 are well-ordered tiled the interior of the hexagon and surrounded the central Pe[3]-1 to form a “Star-of-David” geometry. The mainly driven forces between adjacent macrocycles of each layer in the *ab* plane are C–H···π and C–H···O interactions.

2.2 Crystal engineering of CT co-crystals

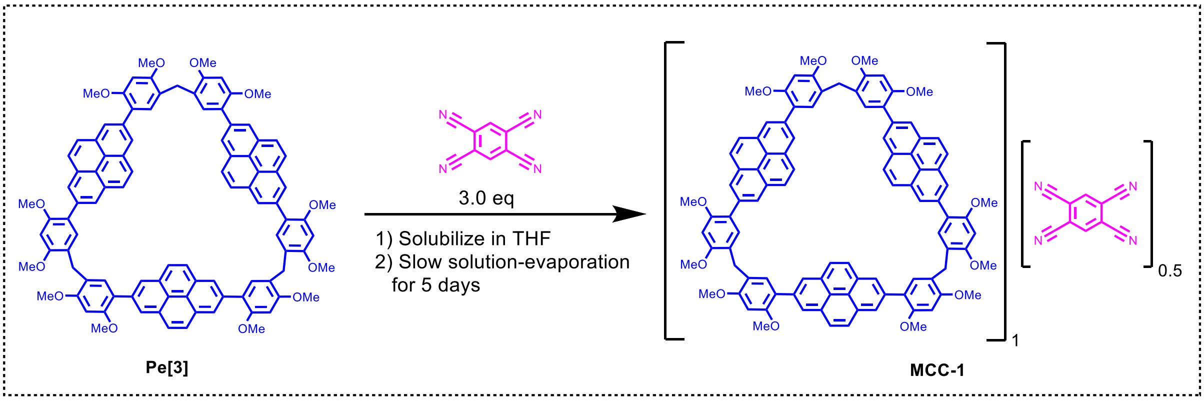

Supplementary Figure 6. Co-crystallization of Pe[3] with TCNB in THF to afford MCC-1.

Method. Pe[3] (10 mg) was first of all mixed with TCNB (3.7 mg) in THF (5 mL) and dissolved by ultrasonic treatment. The solution was filtered with a 0.22-μm syringe filter to remove insoluble impurities. High quality yellow rod-like co-crystals were obtained by slow evaporation of the solution at room temperature after 5 days. The crystals were isolated for single crystal X-ray diffraction. The molar ratio of the Pe[3] and TCNB in the crystal structure was 2:1.

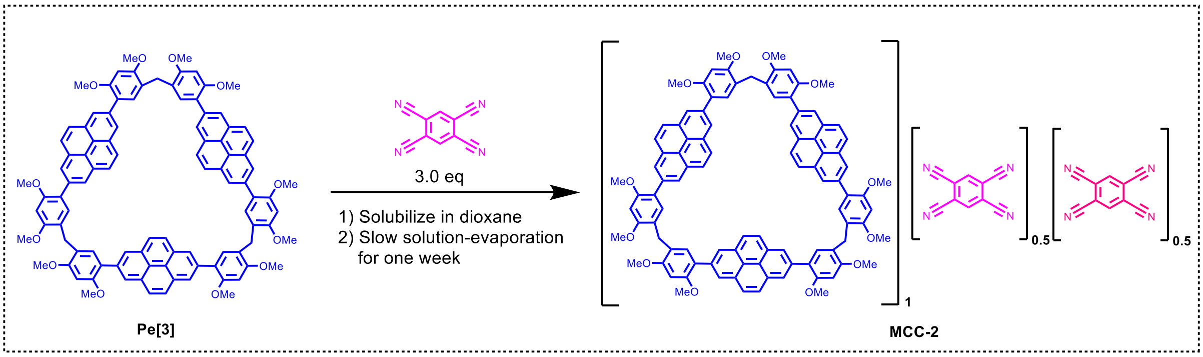

Supplementary Figure 7. Co-crystallization of Pe[3] with TCNB in dioxane to afford MCC-2.

Method. Pe[3] (10 mg) was first of all mixed with TCNB (3.7 mg) in dioxane (5 mL) and dissolved by ultrasonic treatment. The solution was filtered with a 0.22-μm syringe filter to remove insoluble impurities. High quality orange plank-shaped co-crystals were obtained by slow evaporation of the

solution at room temperature after one week. The crystals were isolated for single crystal X-ray diffraction. The molar ratio of the Pe[3] and TCNB in the crystal structure was 1:1.

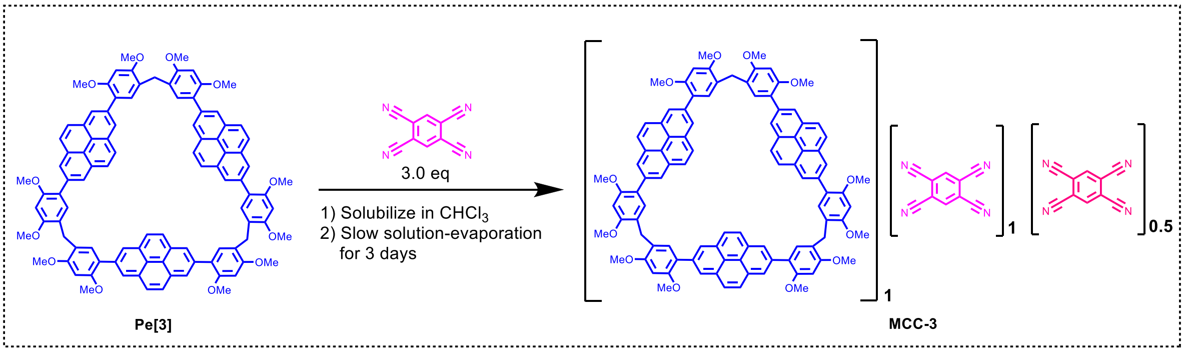

Supplementary Figure 8. Co-crystallization of Pe[3] with TCNB in $CHCl_3$ to obtain MCC-2.

Method. Pe[3] (10 mg) was first of all mixed with TCNB (3.7 mg) in $CHCl_3$ (5 mL) and dissolved by ultrasonic treatment. The solution was filtered with a 0.22-μm syringe filter to remove insoluble impurities. High quality orange plank-shaped co-crystals were obtained by slow evaporation of the solution at room temperature after one week. The crystals were isolated for single crystal X-ray diffraction. The molar ratio of the Pe[3] and TCNB in the crystal structure was 2:3.

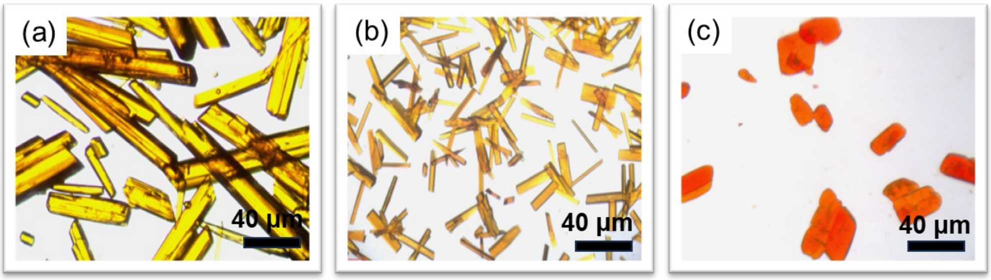

Supplementary Figure 9. Optical microscopy images of (a) MCC-1, (b) MCC-2 and (c) MCC-3. Scale bar: 40 μm.

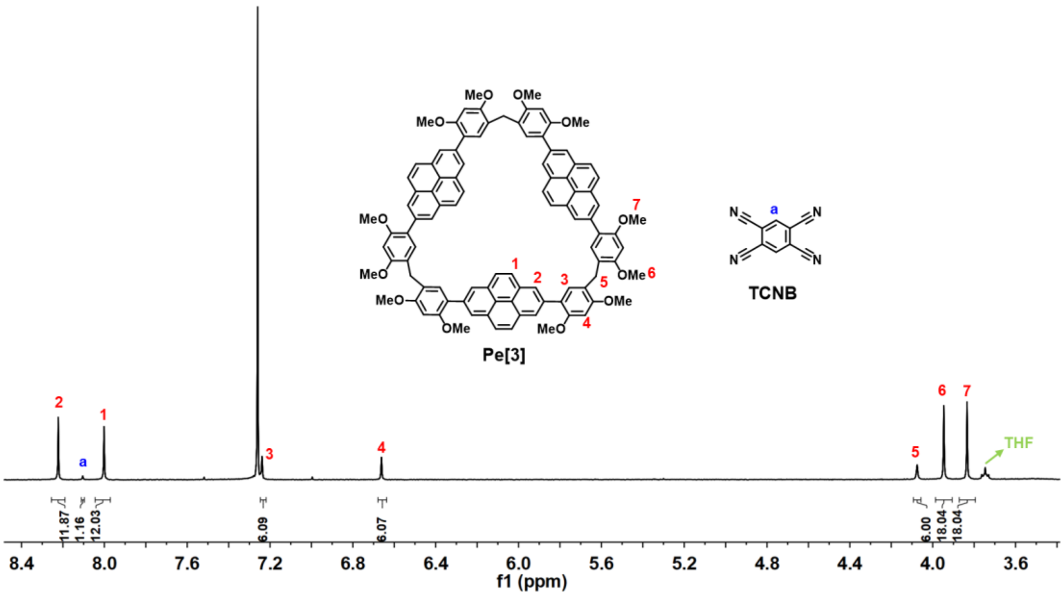

Supplementary Figure 10. ^{1}H NMR spectrum (400 MHz, $CDCl_3$, 298 K) of MCC-1, indicating a strict stoichiometric ratio at 2:1.

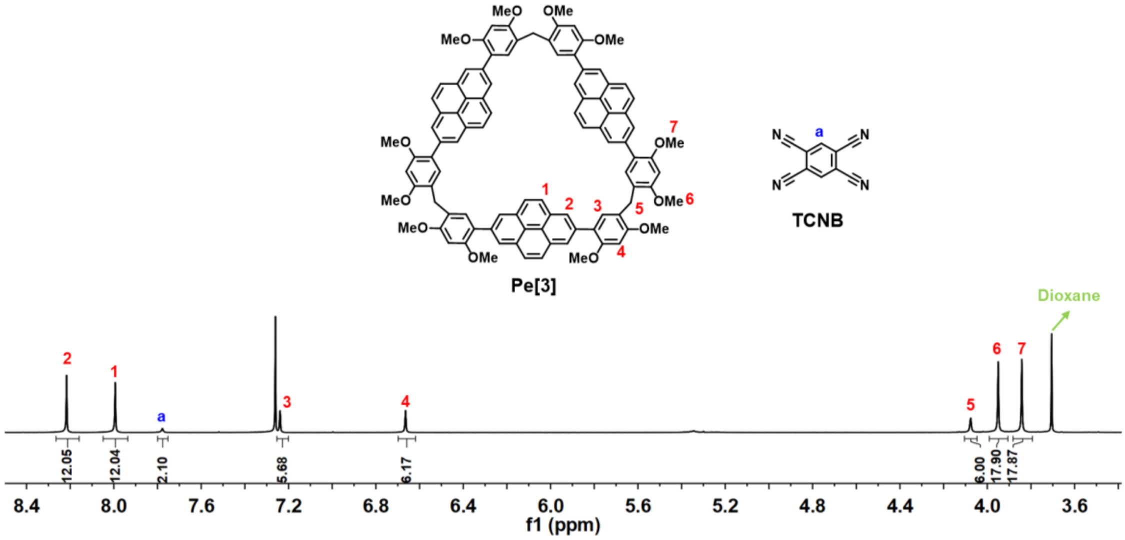

Supplementary Figure 11. ^{1}H NMR spectrum (400 MHz, $CDCl_3$, 298 K) of MCC-2, indicating a strict stoichiometric ratio at 1:1.

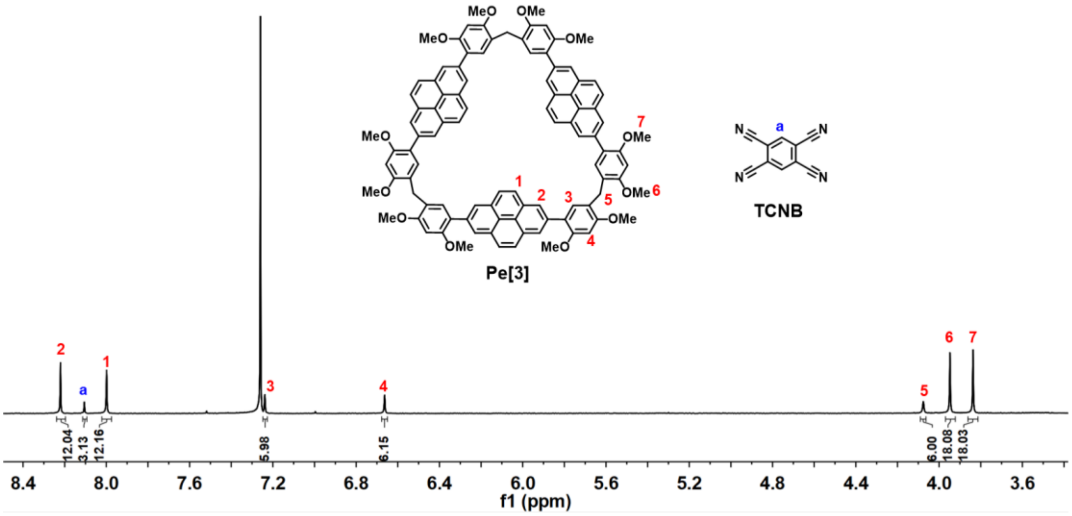

Supplementary Figure 12. ^{1}H NMR spectrum (400 MHz, $CDCl_3$, 298 K) of MCC-3, indicating a strict stoichiometric ratio at 2:3.

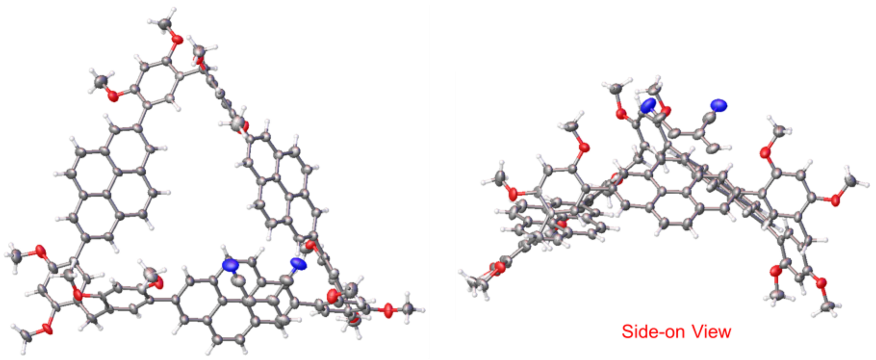

Supplementary Figure 13. ORTEP drawing of MCC-1 with one Pe[3] and one TCNB (with occupancy factor of 0.5) in the asymmetric unit from plane view and side-on view (the thermal ellipsoids are displayed at a 30 % probability). The solvents are omitted for clarity.

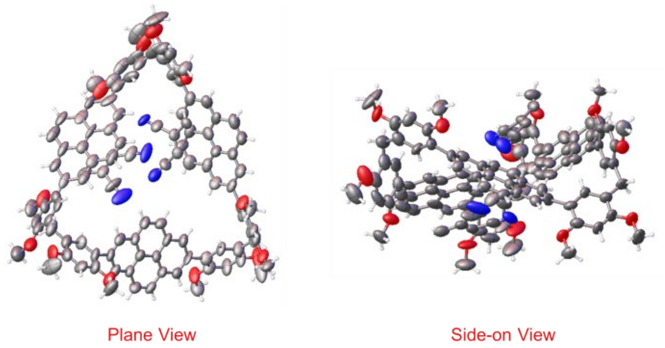

Supplementary Figure 14. ORTEP drawing of MCC-2 with one Pe[3] and two TCNB (with each occupancy factor of 0.5) molecules in the asymmetric unit from plane view and side-on view (the thermal ellipsoids are displayed at a 30 % probability). The solvents are omitted for clarity.

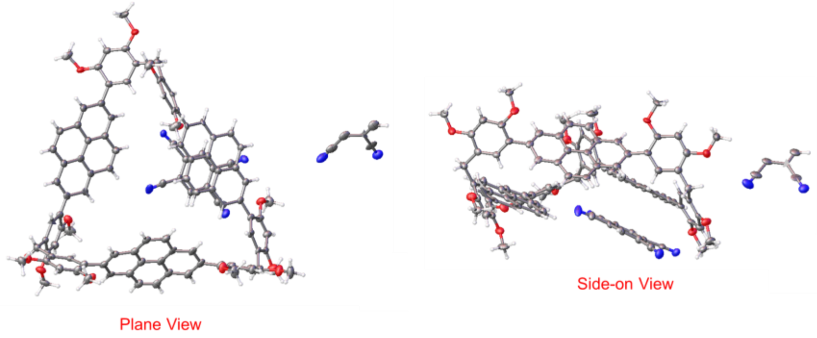

Supplementary Figure 15. ORTEP drawing of MCC-3 with one Pe[3] and 1.5 TCNB molecules in the asymmetric unit from plane view and side-on view (the thermal ellipsoids are displayed at a 30 % probability). The solvents are omitted for clarity.

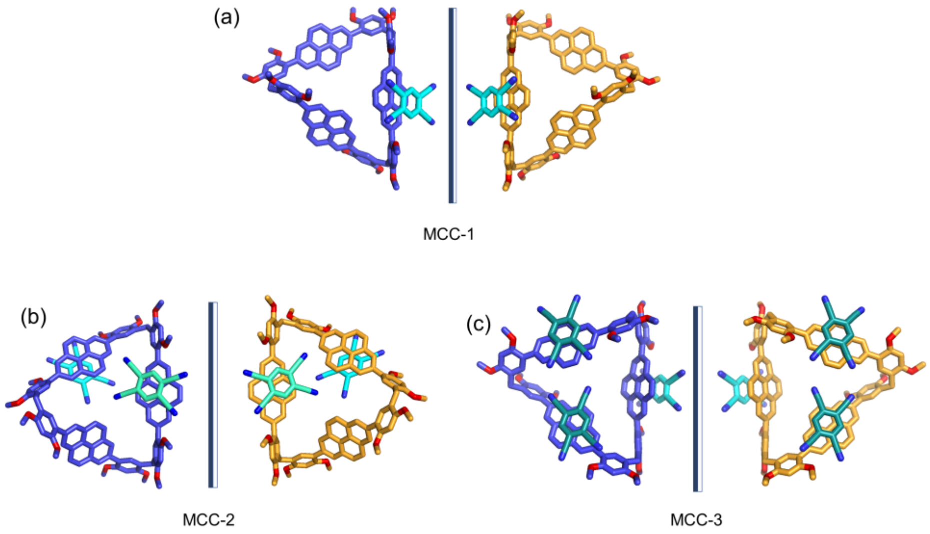

Supplementary Figure 16. The existent one pair of enantiomers in the solid-state structure of MCC-1, MCC-2 and MCC-3. Hydrogen atoms and solvents are omitted for clarity.

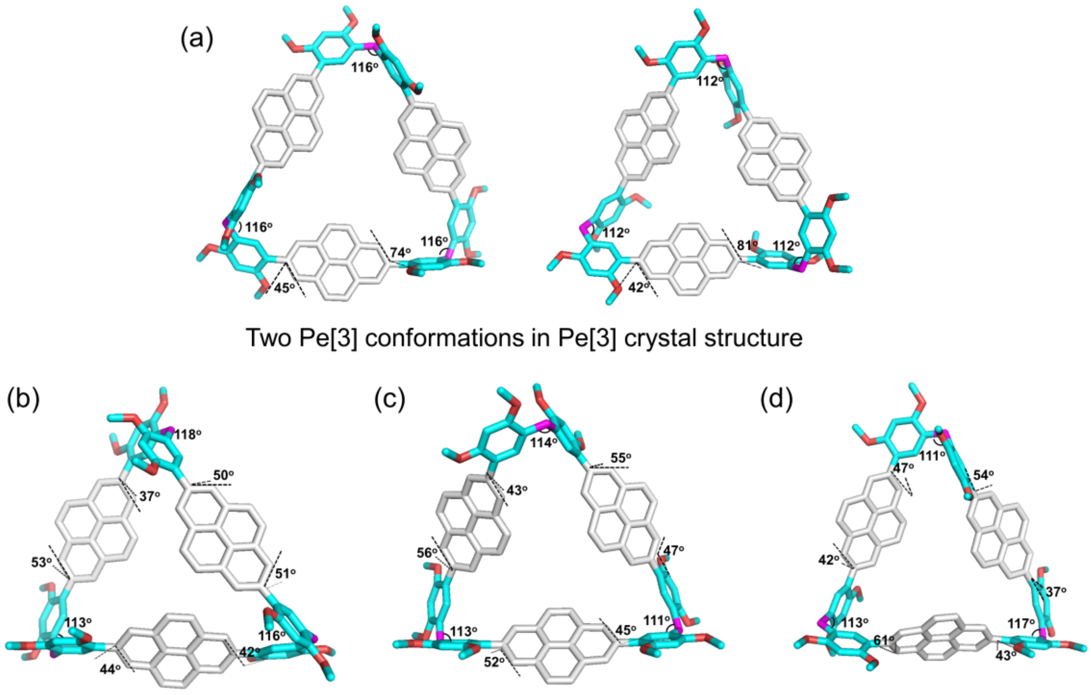

Supplementary Figure 17. Five different conformations of Pe[3] in (a) Pe[3] crystal and (b) MCC-1, (c) MCC-2 and (d) MCC-3 crystal structures, showing that the macrocycle of Pe[3] is flexible and adaptive.

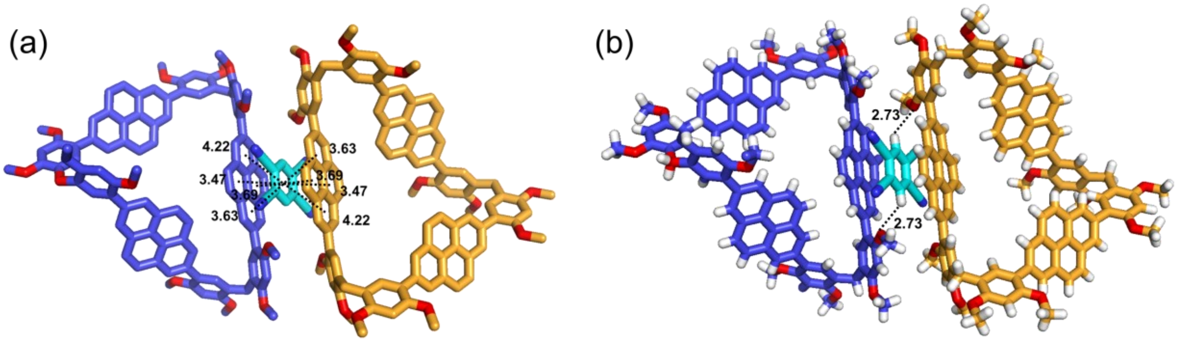

Supplementary Figure 18. (a) π···π [3.47–4.22 Å] and (b) C–H···O [2.73 Å] interactions between Pe[3] and TCNB in the solid-state structure of MCC-1.

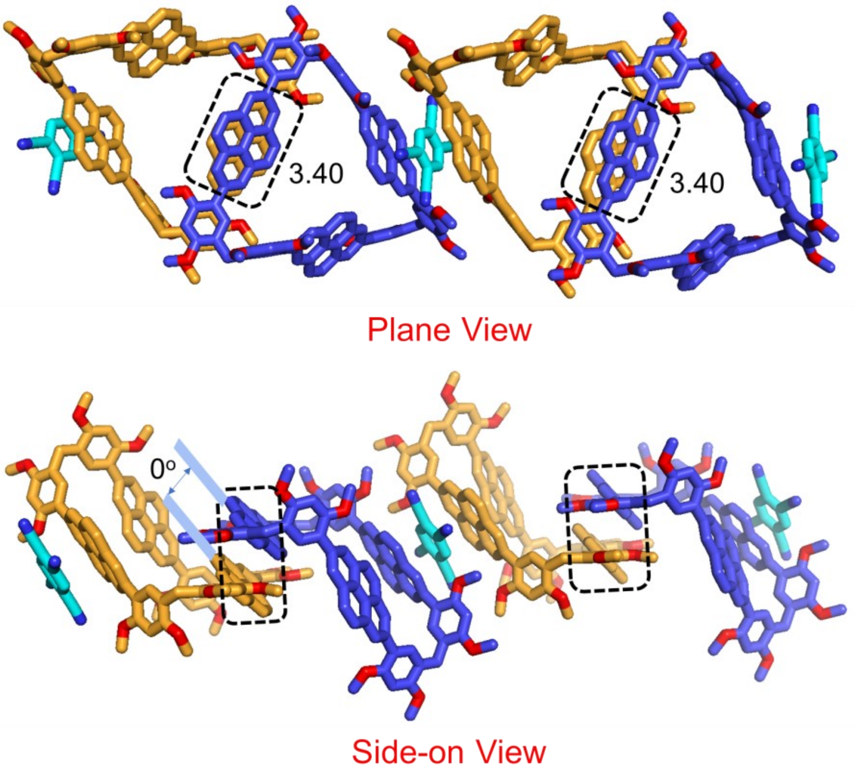

Supplementary Figure 19. Perfect parallel face-to-face π⋯π stacking interactions with average plane–plane distances of 3.40 Å between adjacent Pe[3] in MCC-1. Different colors represent the symmetry equivalence and hydrogen atoms are omitted for clarity.

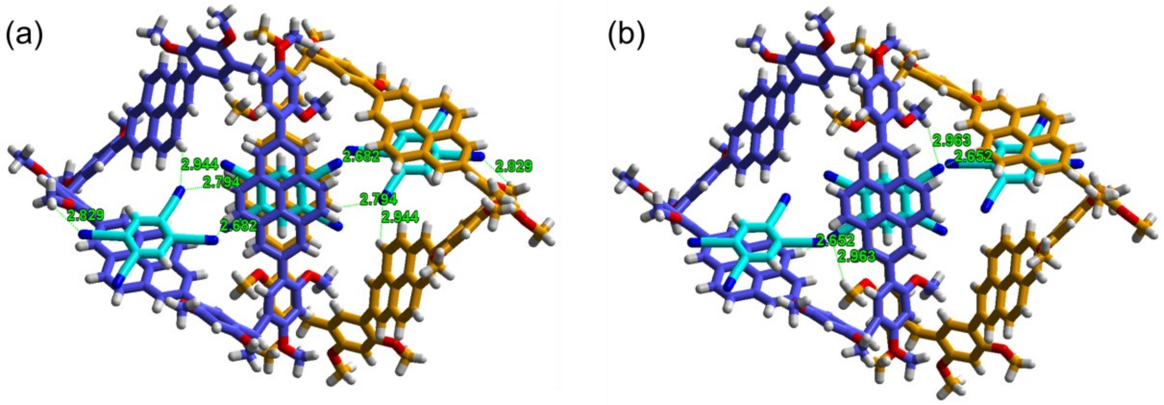

Supplementary Figure 20. (a) C–H⋯N interactions [2.68 Å–2.94 Å] between Pe[3] and TCNB-1. (b) C–H⋯N interactions [2.65 Å–2.96 Å] between Pe[3] and TCNB-2.

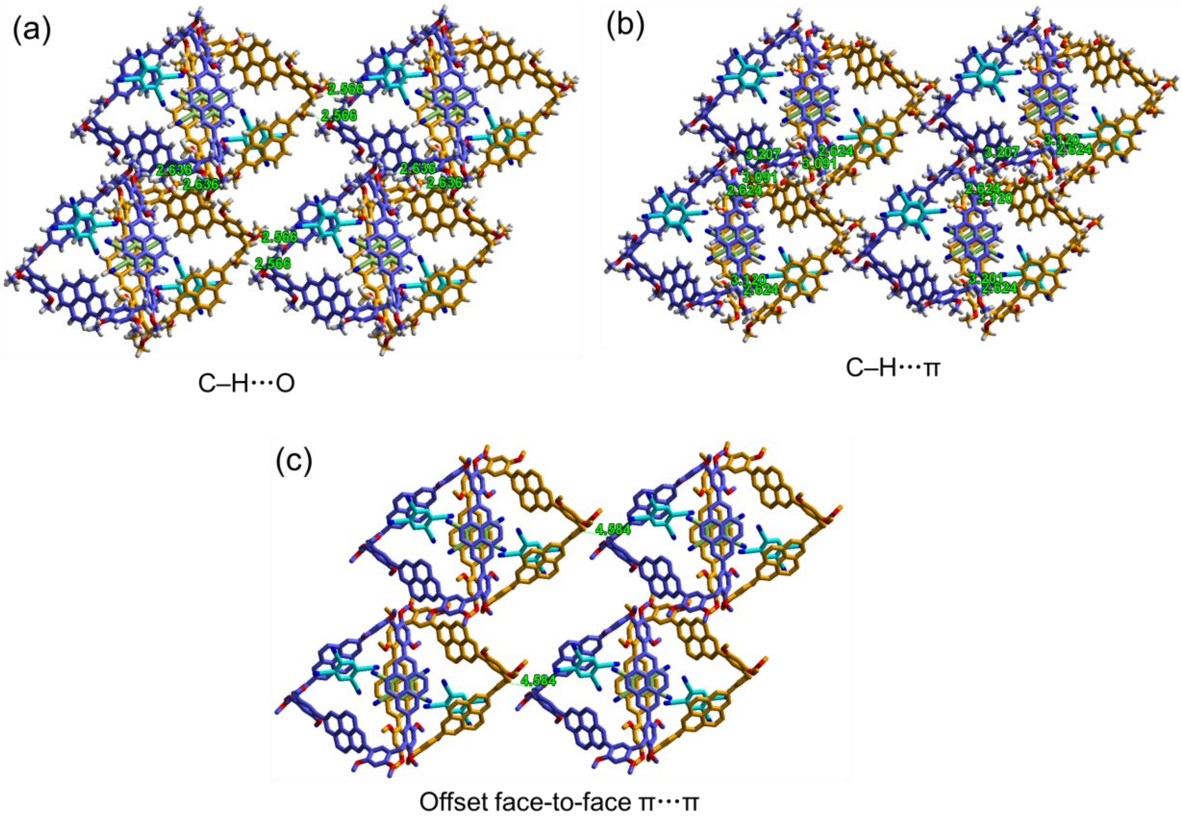

Supplementary Figure 21. The formation of a tetrameric basic unit by (a) the C–H···O [2.57–2.64 Å] interactions, (b) C–H···π [2.62–3.21 Å] interactions and (c) offset face-to-face π···π interactions between adjacent Pe[3] in the MCC-2 crystal structure. Different colors represent the symmetry equivalence.

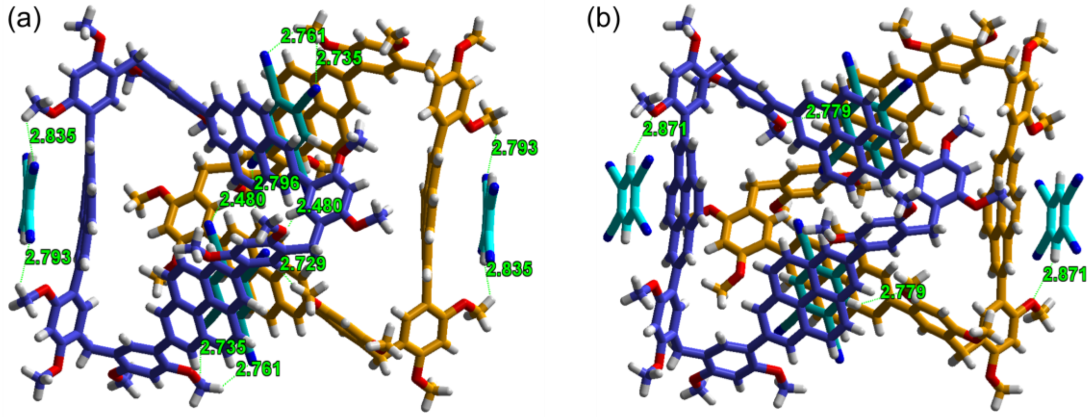

Supplementary Figure 22. (a) C–H···O [2.78–2.87 Å] interactions and (b) C–H···N [2.48–2.84 Å] interactions between Pe[3] with TCNB-3 and TCNB-4 in MCC-3 crystal structure.

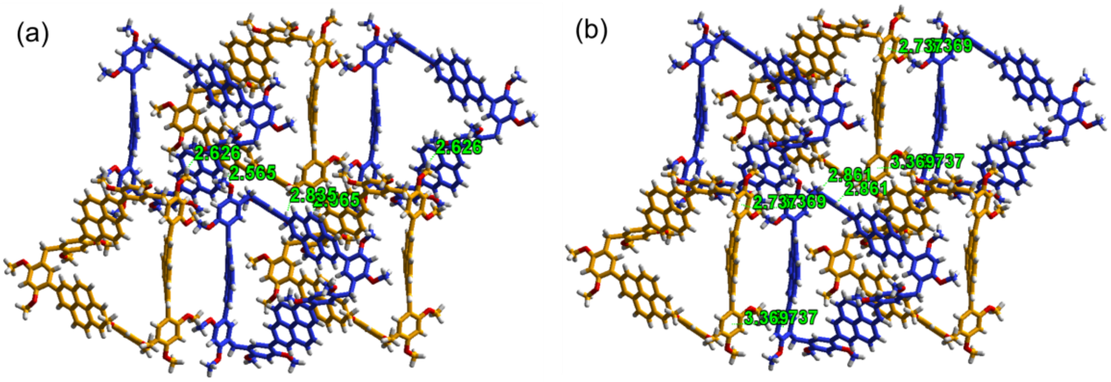

Supplementary Figure 23. (a) C–H···O [2.62–2.84 Å] interactions and (b) C–H···π [2.74–3.37 Å] interactions between adjacent Pe[3] molecules in MCC-3 structure. The TCNB molecules are omitted for clarity. Different colors represent the symmetry equivalence.

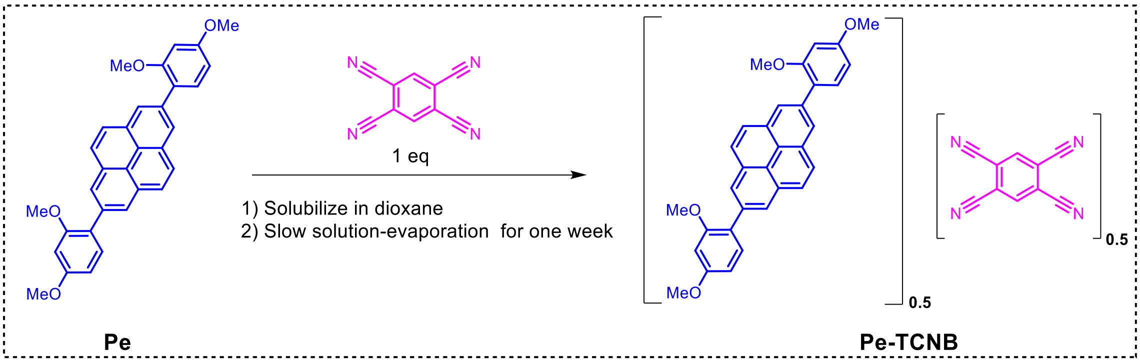

Supplementary Figure 24. Co-crystallization of Pe with TCNB in dioxane to obtain Pe-TCNB.

Method. **Pe** (10 mg) was first of all mixed with TCNB (10 mg) in dioxane (5 mL) and dissolved by ultrasonic treatment. The solution was filtered with a 0.22-μm syringe filter to remove insoluble impurities. High quality orange plank-shaped co-crystals were obtained by slow evaporation of the solution at room temperature after one week. The crystals were isolated for single crystal X-ray diffraction. The molar ratio of the Pe and TCNB in the crystal structure was 1 : 1.

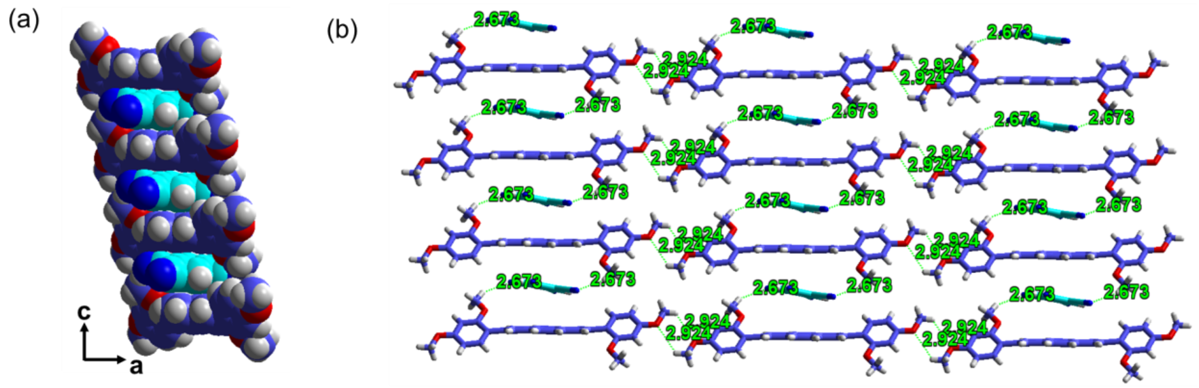

Supplementary Figure 25. (a) A columnlike stacking structure between Pe and TCNB in Pe-TCNB along *b* axis. (b) C–H···N [2.67 Å] interactions between Pe and TCNB, and C–H···O [2.92 Å] interactions between adjacent Pe molecules in Pe-TCNB structure. The solvents are omitted for clarity.

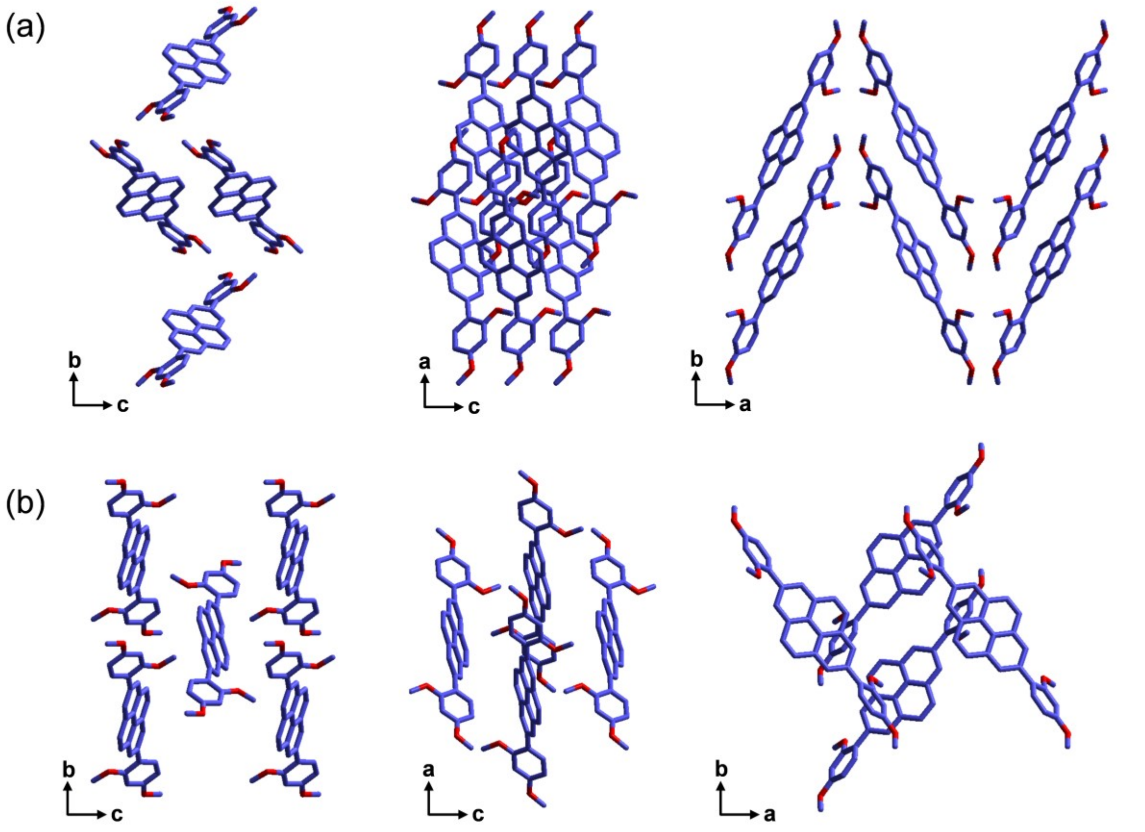

Supplementary Figure 26. The obtained solid-state packing structure of Pe when mix Pe[3] and TCNB in (a) THF and (b) $CHCl_3$, which showed the different molecular stacking in the two solvents. Hydrogen atoms are omitted for clarity.

2.3 Crystallographic data

Supplementary Table 1. Experimental single crystal X-ray data for MCC-1 structure.

Formula	MCC-1	
Crystallization Solvent	THF	
Formula	$C_{148}H_{166}N_2O_{23}$	
Formula weight	2340.82	
Temperature / K	120	
Crystal system	Triclinic	
Space group	*P*-1	
Unit cell dimensions	a = 16.073(3) Å	α = 108.627(5)°
	b = 17.902(3) Å	β = 92.658(6)°
	c = 23.748(4) Å	γ = 102.860(6)
Volume / $Å^3$	6262(2)	
Z	2	
ρ_{calc} g/cm^3	1.242	
μ / mm^{-1}	0.083	
Crystal size / mm^3	0.2 × 0.16 × 0.11	
Radiation	Mo-*K*α (λ = 0.71073 Å)	
F(000)	2330.0	
2Θ range for data collection /°	3.864 to 36.484	
Index ranges	-13 ≤ h ≤ 13, -15 ≤ k ≤ 15, -20 ≤ l ≤ 20	
Reflections collected	47811	
R_{int}	0.0984	
Goodness-of-fit on F^2	1.839	
Final R_1 indexes [I>= 2σ(I)]	0.1386	
Final R_1 indexes [all data]	0.1684	
Final wR(F_2) indexes [all data]	0.4307	
Largest diff. peak/hole / e$Å^{-3}$	0.94/-0.55	
**CCDC number**	**2159214**	

Supplementary Table 2. Experimental single crystal X-ray data for MCC-2 structure.

Formula	MCC-2	
Crystallization Solvent	Dioxane	
Formula	$C_{226}H_{176}N_8O_{28}$	
Formula weight	3451.74	
Temperature / K	150	
Crystal system	Triclinic	
Space group	*P*-1	
Unit cell dimensions	a = 19.465(2) Å	α = 93.593(3)°
	b = 19.978(2) Å	β = 116.099(3)°
	c = 20.219(2) Å	γ = 99.007(3)°
Volume / Å^3	6896.8(14)	
Z	1	
ρ_{calc} g/cm^3	0.831	
μ / mm^{-1}	0.055	
Crystal size / mm^3	0.07 × 0.05 × 0.03	
Radiation	Mo-*K*α (λ = 0.71073 Å)	
F(000)	1812.0	
2Θ range for data collection /°	3.968 to 49.426	
Index ranges	-22 ≤ h ≤ 22, -23 ≤ k ≤ 23, -23 ≤ l ≤ 23	
Reflections collected	69344	
R_{int}	0.1349	
Goodness-of-fit on F^2	1.009	
Final R_1 indexes [I>= 2σ(I)]	0.1280	
Final R_1 indexes [all data]	0.2750	
Final wR(F_2) indexes [all data]	0.4213	
Largest diff. peak/hole / eÅ^{-3}	0.41/-0.35	
**CCDC number**	**2159210**	

Supplementary Table 3. Experimental single crystal X-ray data for MCC-3 structure.

Formula	MCC-3	
Crystallization Solvent	$CHCl_3$	
Formula	$C_{121}H_{88}Cl_{21}N_6O_{12}$	
Formula weight	2562.42	
Temperature / K	120	
Crystal system	Triclinic	
Space group	*P*-1	
Unit cell dimensions	a = 17.933(3) Å	α = 72.498(5)°
	b = 19.019(3) Å	β = 70.940(4)°
	c = 23.176(4) Å	γ = 75.284(5)°
Volume / $Å^3$	7017.8(18)	
Z	2	
ρ_{calc} g/cm^3	1.213	
μ / mm^{-1}	0.461	
Crystal size / mm^3	0.2 × 0.16 × 0.12	
Radiation	Mo-*K*α (λ = 0.71073 Å)	
F(000)	2618.0	
2Θ range for data collection /°	3.828 to 41.812	
Index ranges	-17 ≤ h ≤ 17, -19 ≤ k ≤ 19, -23 ≤ l ≤ 23	
Reflections collected	99675	
R_{int}	0.1063	
Goodness-of-fit on F^2	1.058	
Final R_1 indexes [I>= 2σ(I)]	0.1292	
Final R_1 indexes [all data]	0.1448	
Final wR(F_2) indexes [all data]	0.3453	
Largest diff. peak/hole / eÅ^{-3}	2.45/-1.08	
**CCDC number**	**2159192**	

Supplementary Table 4. Experimental single crystal X-ray data for Pe-TCNB structure in dioxane.

Formula	Pe-TCNB	
Crystallization Solvent	Dioxane	
Formula	$C_{46}H_{36}N_4O_6$	
Formula weight	740.79	
Temperature / K	296.15	
Crystal system	Triclinic	
Space group	P-1	
Unit cell dimensions	a = 7.5708(9) Å	α = 67.249(2)°
	b = 11.5989(13) Å	β = 89.497(2)°
	c = 11.9686(14) Å	γ = 89.497(2)°
Volume / $Å^3$	948.82(19)	
Z	1	
ρ_{calc} g/cm^3	1.296	
μ / mm^{-1}	0.087	
Crystal size / mm^3	0.21 × 0.2 × 0.18	
Radiation	Mo-*K*α (λ = 0.71073 Å)	
F(000)	388.0	
2Θ range for data collection /°	3.818 to 55.422	
Index ranges	-9 ≤ h ≤ 9, -8 ≤ k ≤ 15, -9 ≤ l ≤ 15	
Reflections collected	5799	
R_{int}	0.0126	
Goodness-of-fit on F^2	1.031	
Final R_1 indexes [I>= 2σ(I)]	0.0563	
Final R_1 indexes [all data]	0.0902	
Final wR(F_2) indexes [all data]	0.1757	
Largest diff. peak/hole / eÅ^{-3}	0.86/-0.35	
**CCDC number**	**2235489**	

Supplementary Table 5. Experimental single crystal X-ray data for Pe structure in THF.

Formula	Pe	
Crystallization Solvent	THF	
Formula	$C_{32}H_{26}O_4$	
Formula weight	474.53	
Temperature / K	296	
Crystal system	monoclinic	
Space group	$P2_1/c$	
Unit cell dimensions	a = 8.733(4) Å	α = 90°
	b = 19.954(9) Å	β = 102.94(3)°
	c = 7.088(3) Å	γ = 90°
Volume / $Å^3$	1203.8(9)	
Z	2	
ρ_{calc} g/cm^3	1.309	
μ / mm^{-1}	0.085	
Crystal size / mm^3	0. 11 × 0.12 × 0.13	
Radiation	Mo-*K*α (λ = 0.71073 Å)	
F(000)	500.0	
2Θ range for data collection /°	4.886 to 55.406	
Index ranges	-11 ≤ h ≤ 11, -21 ≤ k ≤ 26, -9 ≤ l ≤ 9	
Reflections collected	11470	
R_{int}	0.0621	
Goodness-of-fit on F^2	1.037	
Final R_1 indexes [I>= 2σ(I)]	0.0491	
Final R_1 indexes [all data]	0.0636	
Final wR(F_2) indexes [all data]	0.1488	
Largest diff. peak/hole / eÅ^{-3}	0.28/-0.19	
**CCDC number**	**2285147**	

Supplementary Table 6. Experimental single crystal X-ray data for Pe structure in $CHCl_3$.

Formula	Pe
Crystallization Solvent	$CHCl_3$
Formula	$C_{32}H_{26}O_4$
Formula weight	474.53
Temperature / K	296
Crystal system	monoclinic
Space group	$P2_1/n$
Unit cell dimensions	a = 8.7173(8) Å α = 90°
	b = 11.8963(11) Å β = 94.858(1)°
	c = 11.7292(11) Å γ = 90°
Volume / Å^3	1211.99(19)
Z	2
ρ_{calc} g/cm^3	1.300
μ / mm^{-1}	0.085
Crystal size / mm^3	0. 1 × 0.11 × 0.12
Radiation	Mo-*K*α (λ = 0.71073 Å)
F(000)	1704.0
2Θ range for data collection /°	4.886 to 55.406
Index ranges	-11 ≤ h ≤ 10, -14 ≤ k ≤ 14, -13 ≤ l ≤ 15
Reflections collected	7193
R_{int}	0.0235
Goodness-of-fit on F^2	1.062
Final R_1 indexes [I>= 2σ(I)]	0.0482
Final R_1 indexes [all data]	0.0612
Final wR(F_2) indexes [all data]	0.1366
Largest diff. peak/hole / eÅ^{-3}	0.26/-0.40
**CCDC number**	**2285121**

2.4 Photophysical characterizations

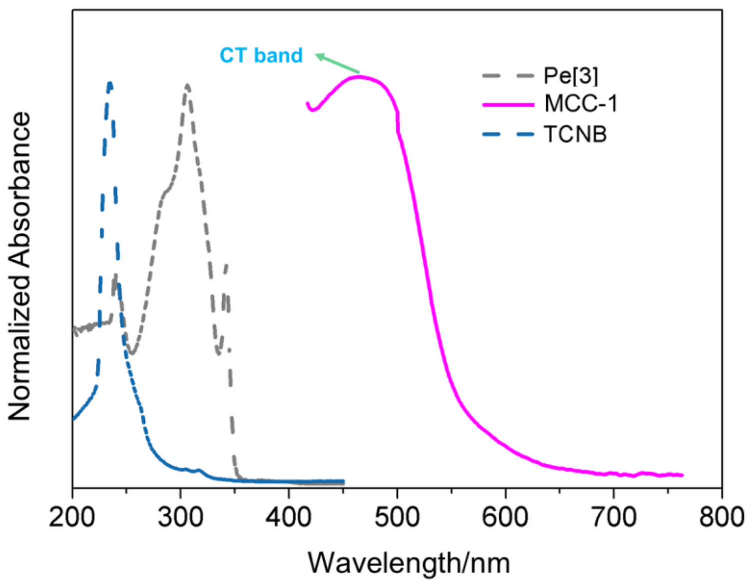

Supplementary Figure 27. Solid-state UV-Vis absorption spectra of Pe[3], TCNB and MCC-1, showing a CT band at 465 nm with red-shift of 159 and 230 nm compared with Pe[3] and TCNB, respectively.

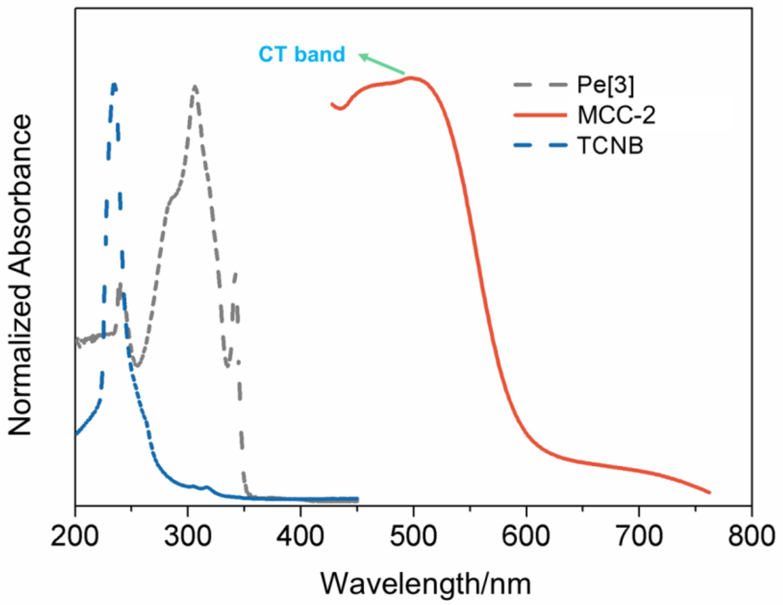

Supplementary Figure 28. Solid-state UV-Vis absorption spectra of Pe[3], TCNB and MCC-2, showing a CT band at 497 nm with red-shift of 191 and 262 nm compared with Pe[3] and TCNB, respectively.

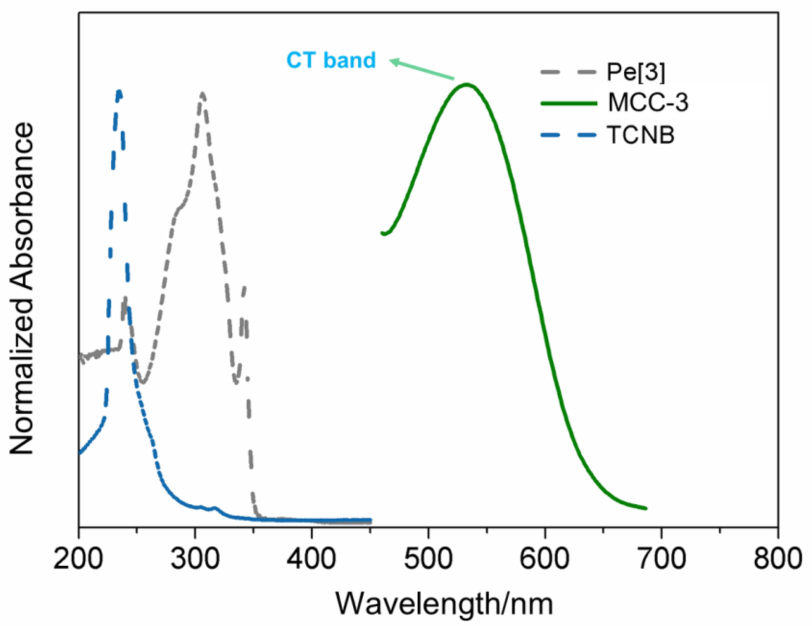

Supplementary Figure 29. Solid-state UV-Vis absorption spectra of Pe[3], TCNB and MCC-3, showing a CT band at 532 nm with red-shift of 226 and 297 nm compared with Pe[3] and TCNB, respectively.

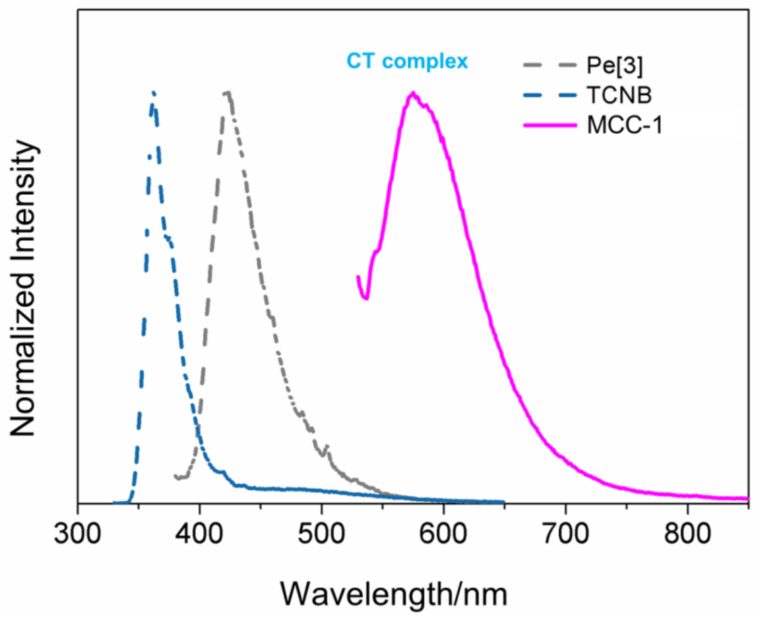

Supplementary Figure 30. Solid-state fluorescence spectra of Pe[3], TCNB and MCC-1, showing a new emission peak at 575 nm.

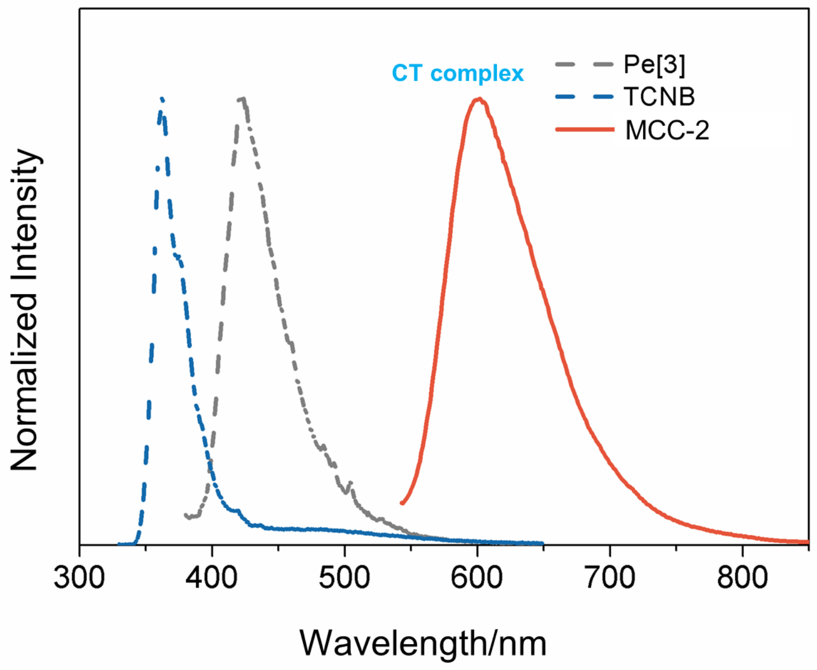

Supplementary Figure 31. Solid-state fluorescence spectra of Pe[3], TCNB and MCC-2, showing a new emission peak at 602 nm.

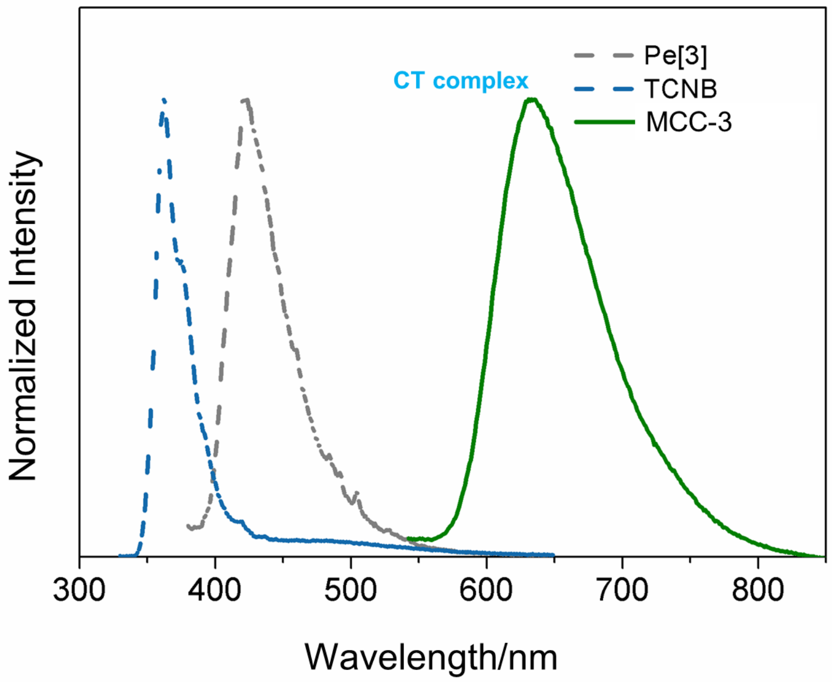

Supplementary Figure 32. Solid-state fluorescence spectra of Pe[3], TCNB and MCC-3, showing a new emission peak at 635 nm.

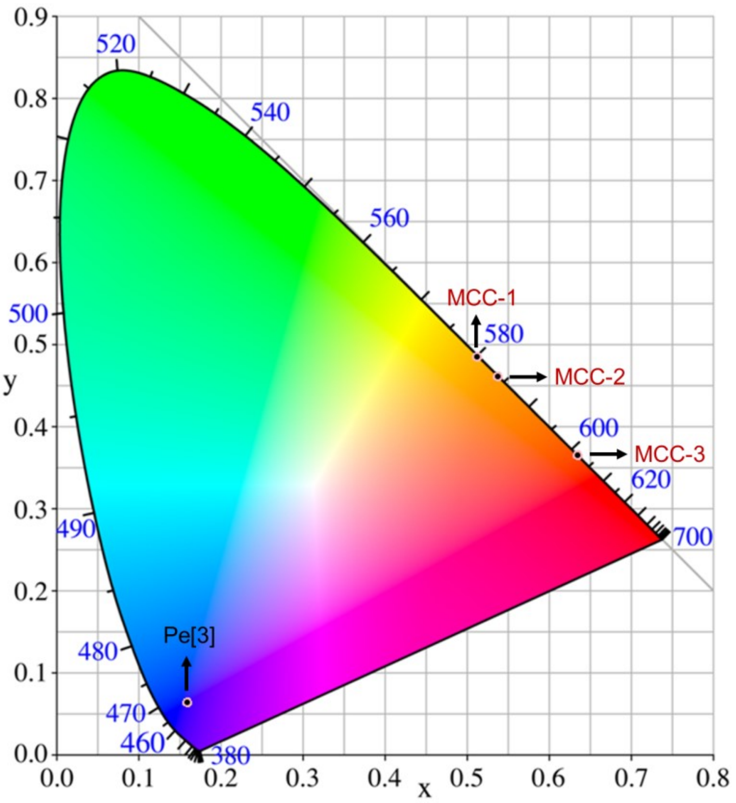

Supplementary Figure 33. CIE coordinates of Pe[3] (0.16, 0.06), MCC-1 (0.51, 0.48), MCC-2 (0.54, 0.46) and MCC-3 (0.63, 0.37).

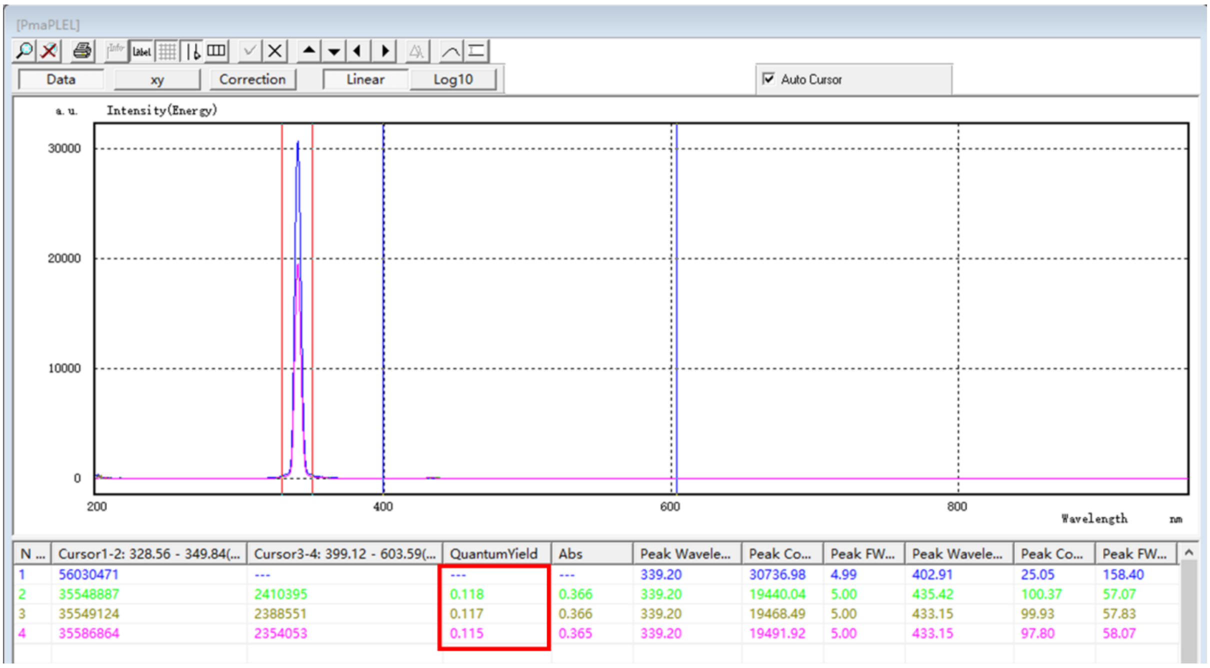

Supplementary Figure 34. Quantum yield of Pe[3].

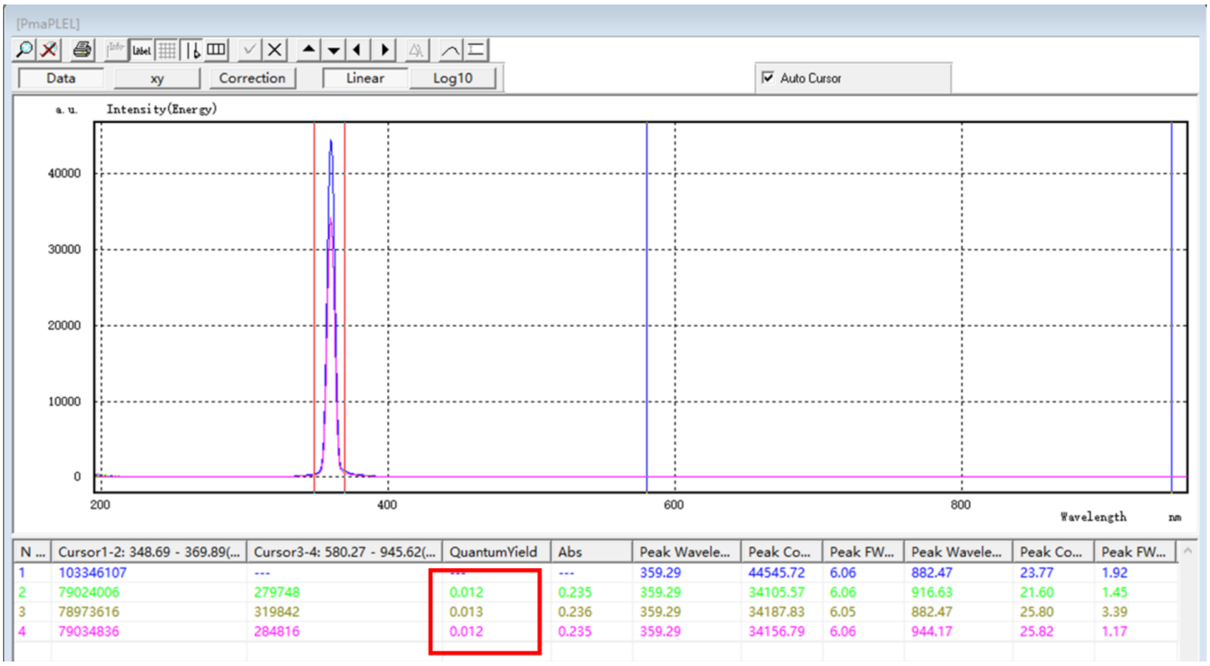

Supplementary Figure 35. Quantum yield of MCC-1.

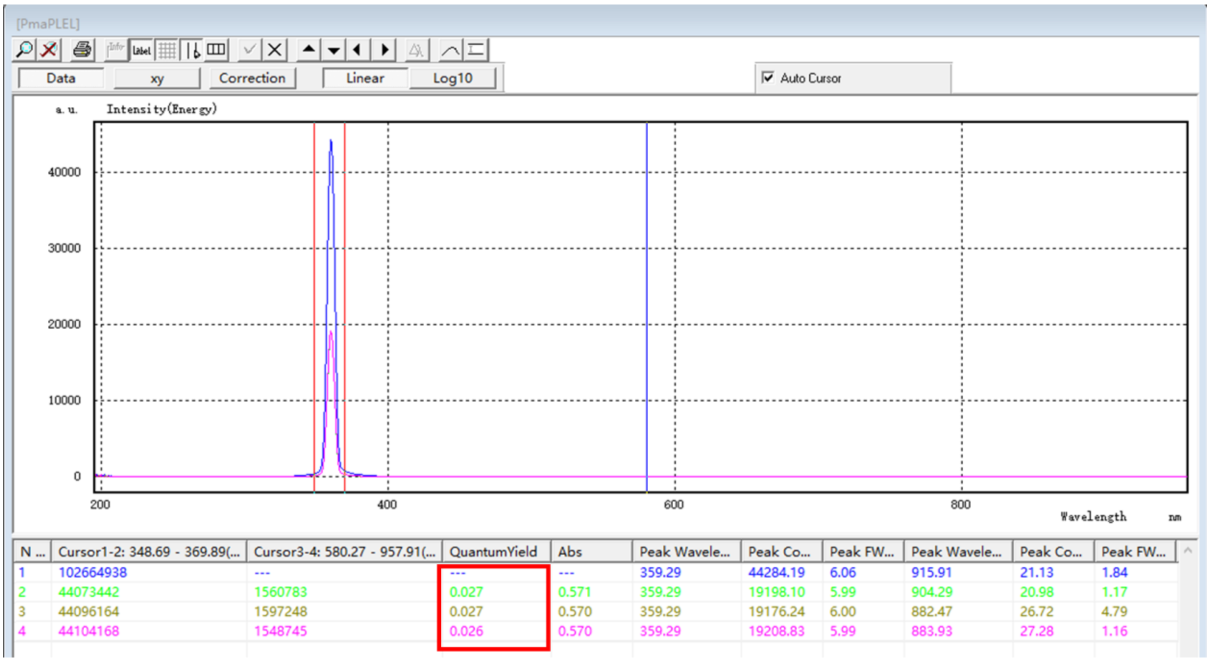

Supplementary Figure 36. Quantum yield of MCC-2.

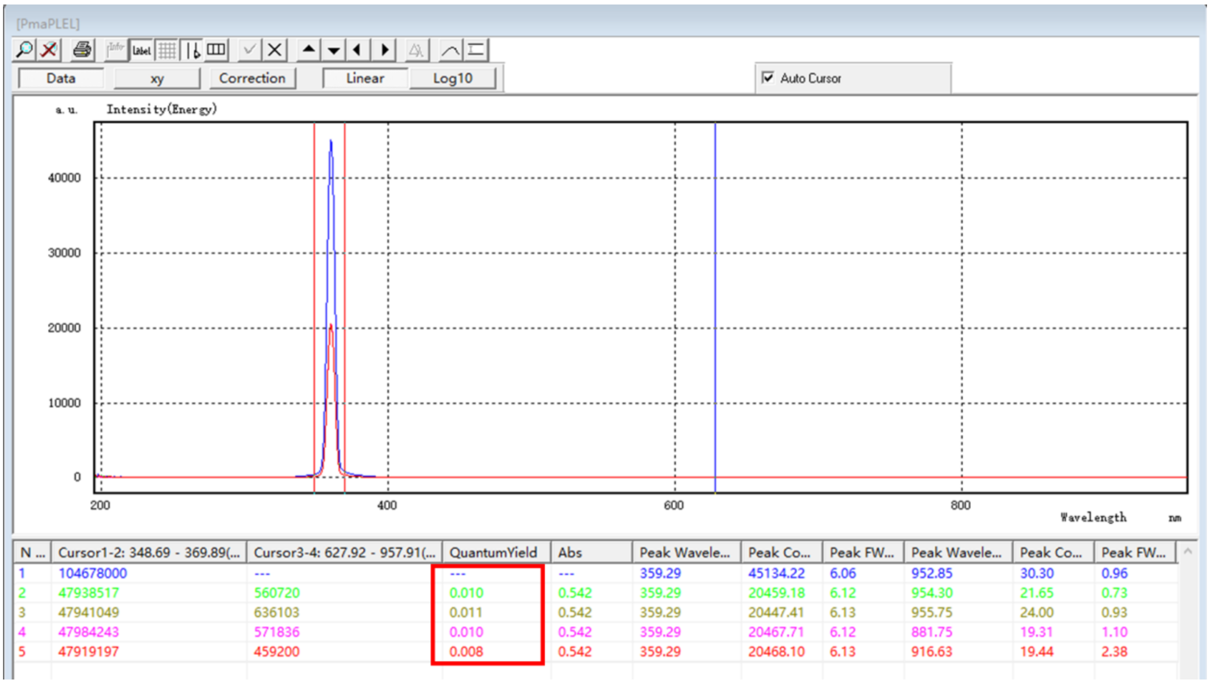

Supplementary Figure 37. Quantum yield of MCC-3.

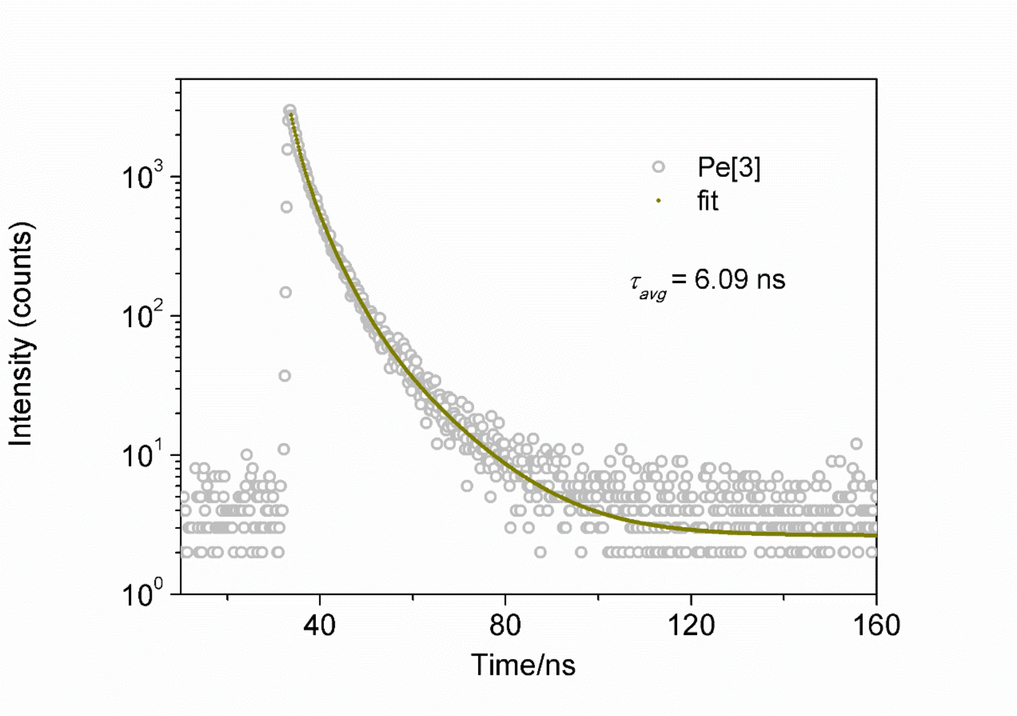

Supplementary Figure 38. Time-resolved fluorescence decay curve of Pe[3] at 424 nm in the solid state.

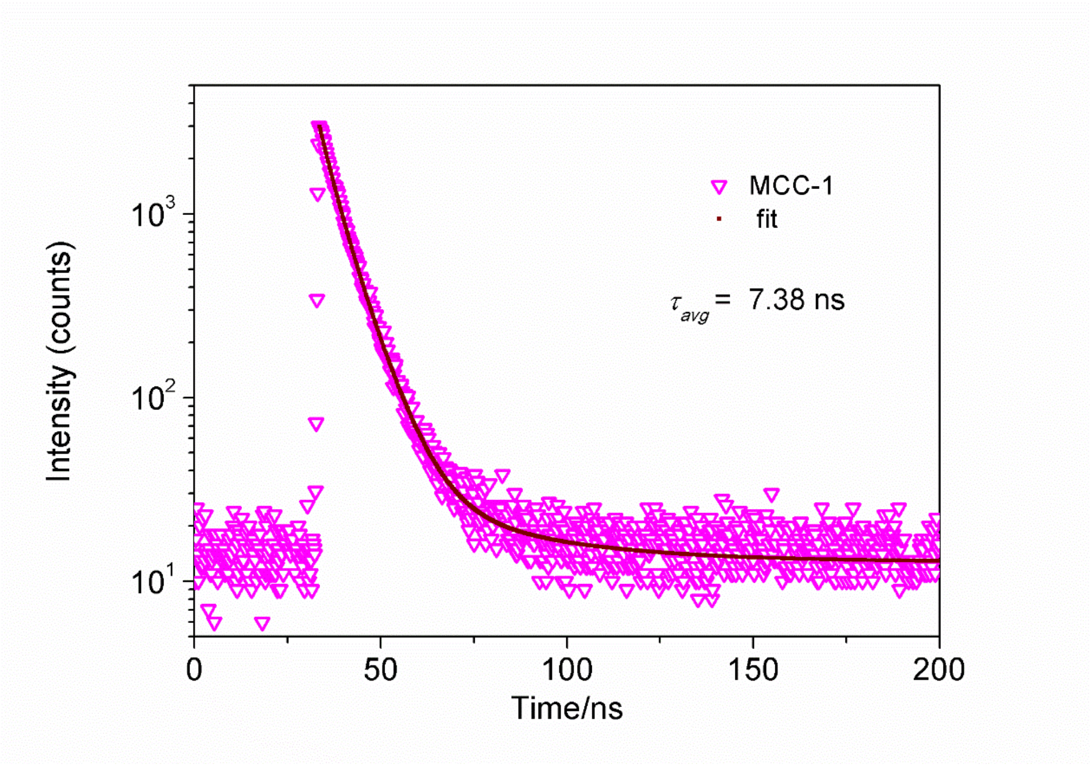

Supplementary Figure 39. Time-resolved fluorescence decay curve of MCC-1 at 575 nm in the solid state.

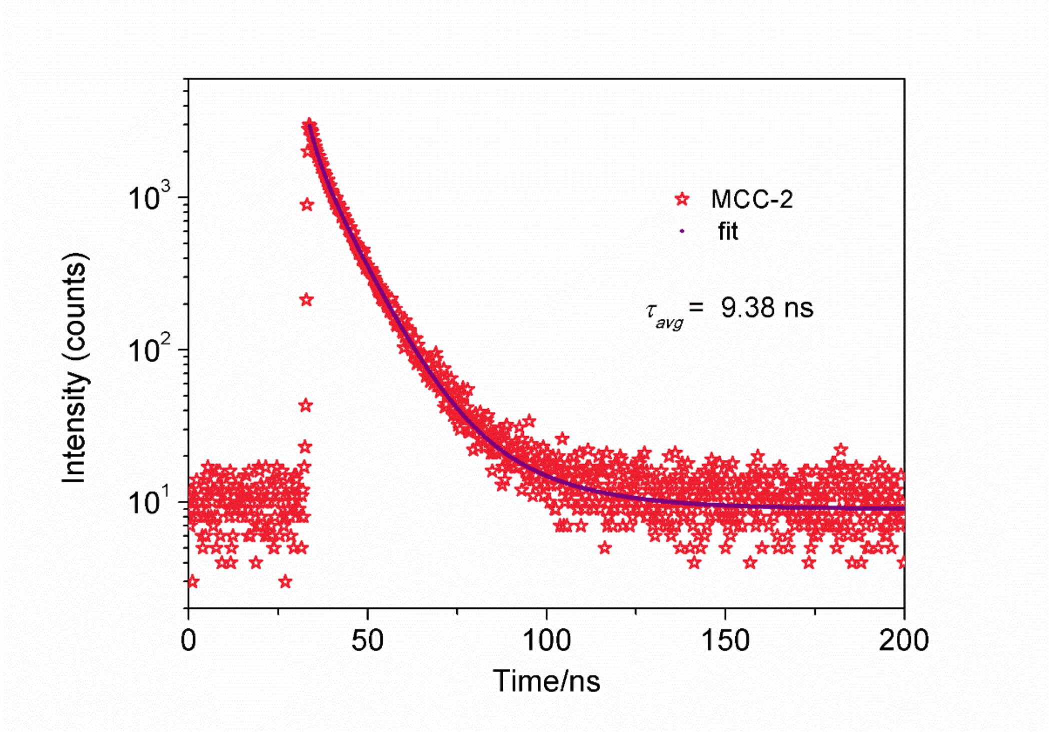

Supplementary Figure 40. Time-resolved fluorescence decay curve of MCC-2 at 602 nm in the solid state.

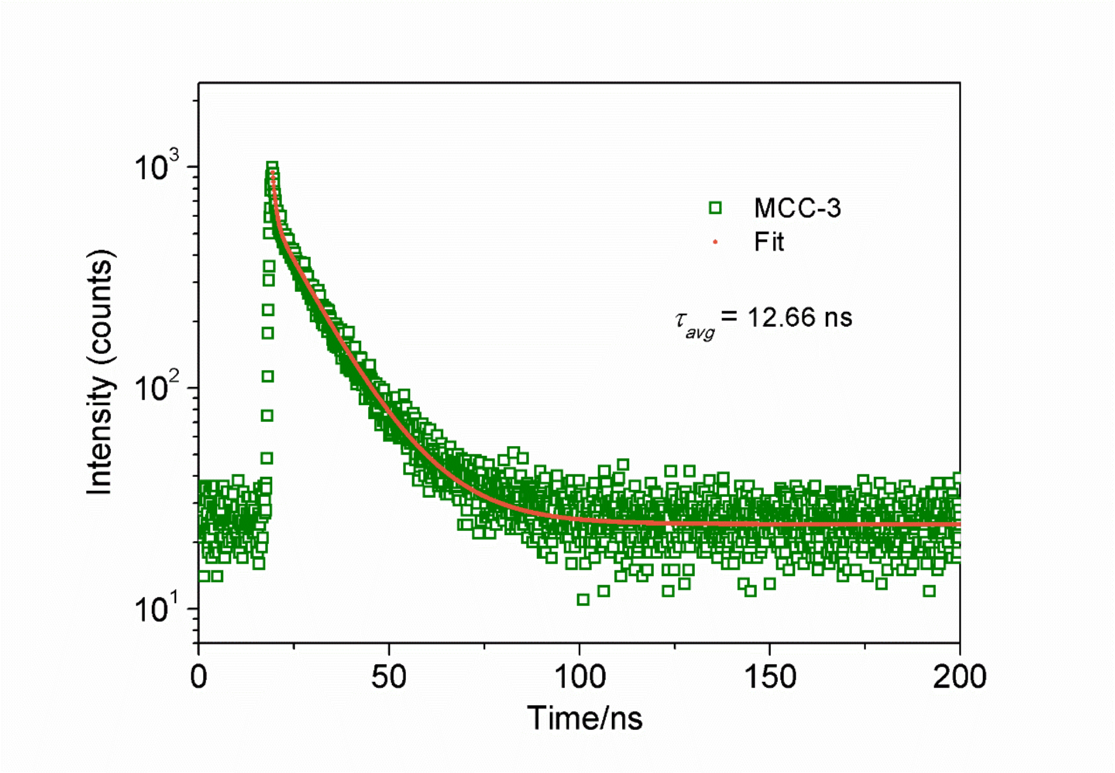

Supplementary Figure 41. Time-resolved fluorescence decay curve of MCC-3 at 635 nm in the solid state.

2.5 DFT calculation

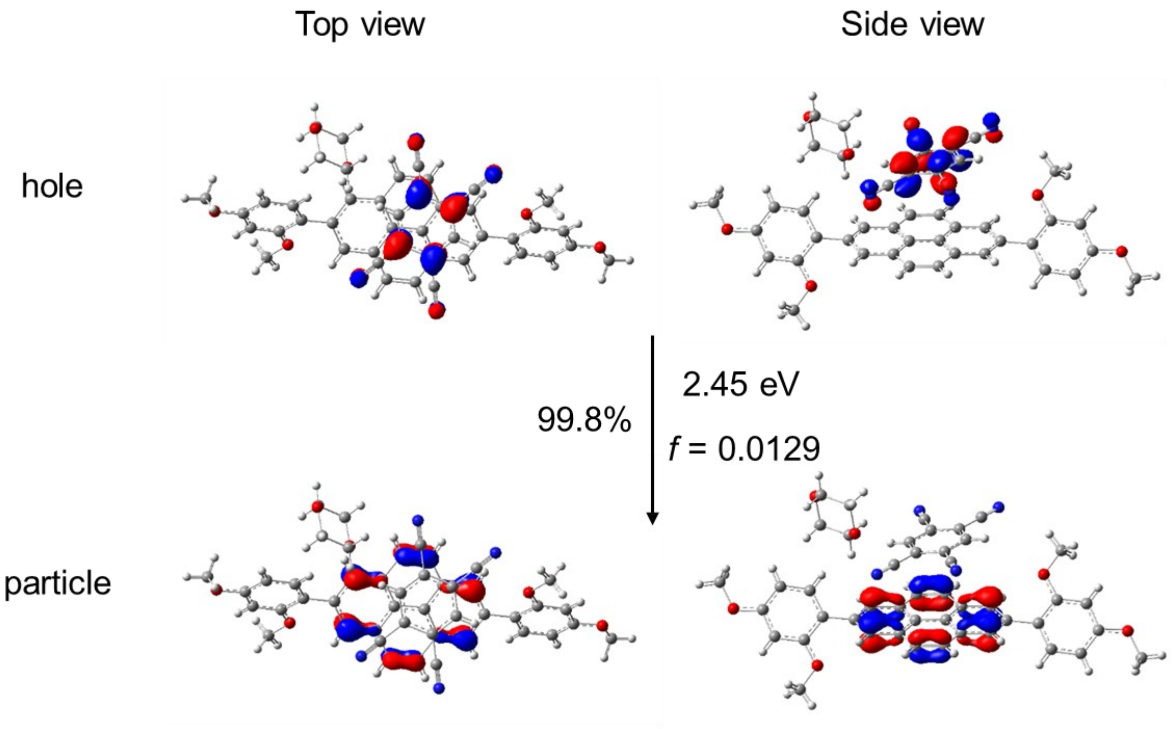

Supplementary Figure 42. The dominant natural transition orbital (NTO) pairs for the first excited singlet state (the absorption peak is at 506 nm, and oscillator strength value is 0.0129). The "hole" is on the top, and the "particle" is on the bottom. The DFT and TDDFT calculation were carried out at CAM-B3LYP/6-311G* level using the Gaussian 16 suite

To characterize the excited state of the co-crystals, the electronic transition densities were also calculated by the natural transition orbitals (NTOs) analysis. We selected the co-crystal structure of Pe-TCNB instead of MCCs as the calculation model because the structures of MCCs are solvent-rich and complex, which is too difficult to calculate the excitation process. As shown in Supplementary Figure 42, the holes were delocalized over TCNB, and the particles were localized over pyrene moiety on Pe, implying CT emission. From a quantitative perspective, the model is in good agreement with the experimental results (Supplementary Figure 43).

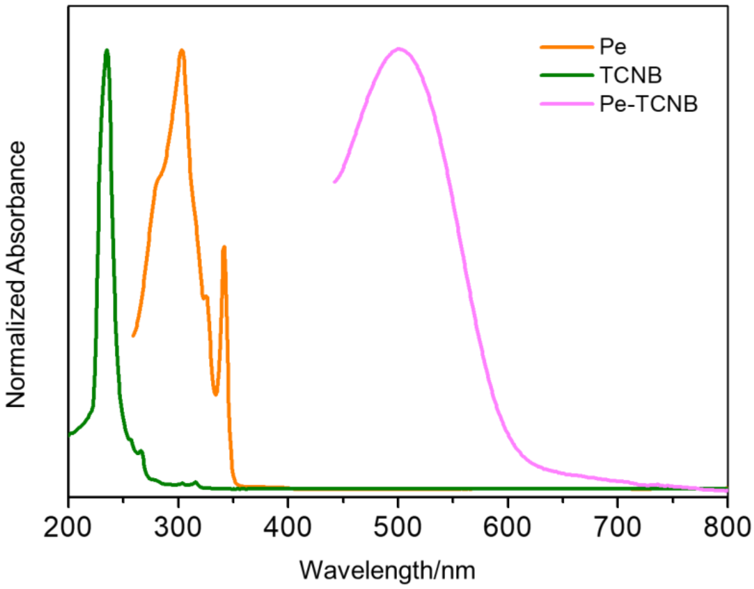

Supplementary Figure 43. Solid-state UV-Vis absorption spectra of Pe, TCNB and Pe-TCNB, showing a CT band at 504 nm.

2.6 Mechanism of MCCs formation at different stoichiometries

Supplementary Table 7. The solubility of Pe[3] and TCNB and their D-A stoichiometric ratios of MCCs in different solvents (mean ± SD, n = 3).

Solvents	Solubility of Pe[3] mmol L^{-1}	Solubility of TCNB mmol L^{-1}	Stoichiometric ratios of Pe[3]:TCNB
$CHCl_3$	9.9 ± 0.48	6.6 ± 0.32	2 : 3
o-Xylene	9.1 ± 0.59	7.7 ± 1.17	2 : 3
CH_2Cl_2	2.6 ± 0.41	41 ± 2.6	1 : 1
Benzene	2.8 ± 0.45	49 ± 2.3	1 : 1
2,3-Dihydrofuran	3.2 ± 0.41	59 ± 3.9	1 : 1
1,4-Dioxane	3.9 ± 0.53	85 ± 8.8	1 : 1
$ClCH_2CH_2Cl$	5.9 ± 0.64	277 ± 11	2 : 1
THF	4.4 ± 0.43	366 ± 11	2 : 1
1,3-Dioxane	4.6 ± 0.64	436 ± 5	2 : 1
DMSO	2.5 ± 0.28	1207 ± 45	1 : 0
DMF	2.3 ± 0.27	1329 ± 20	1 : 0

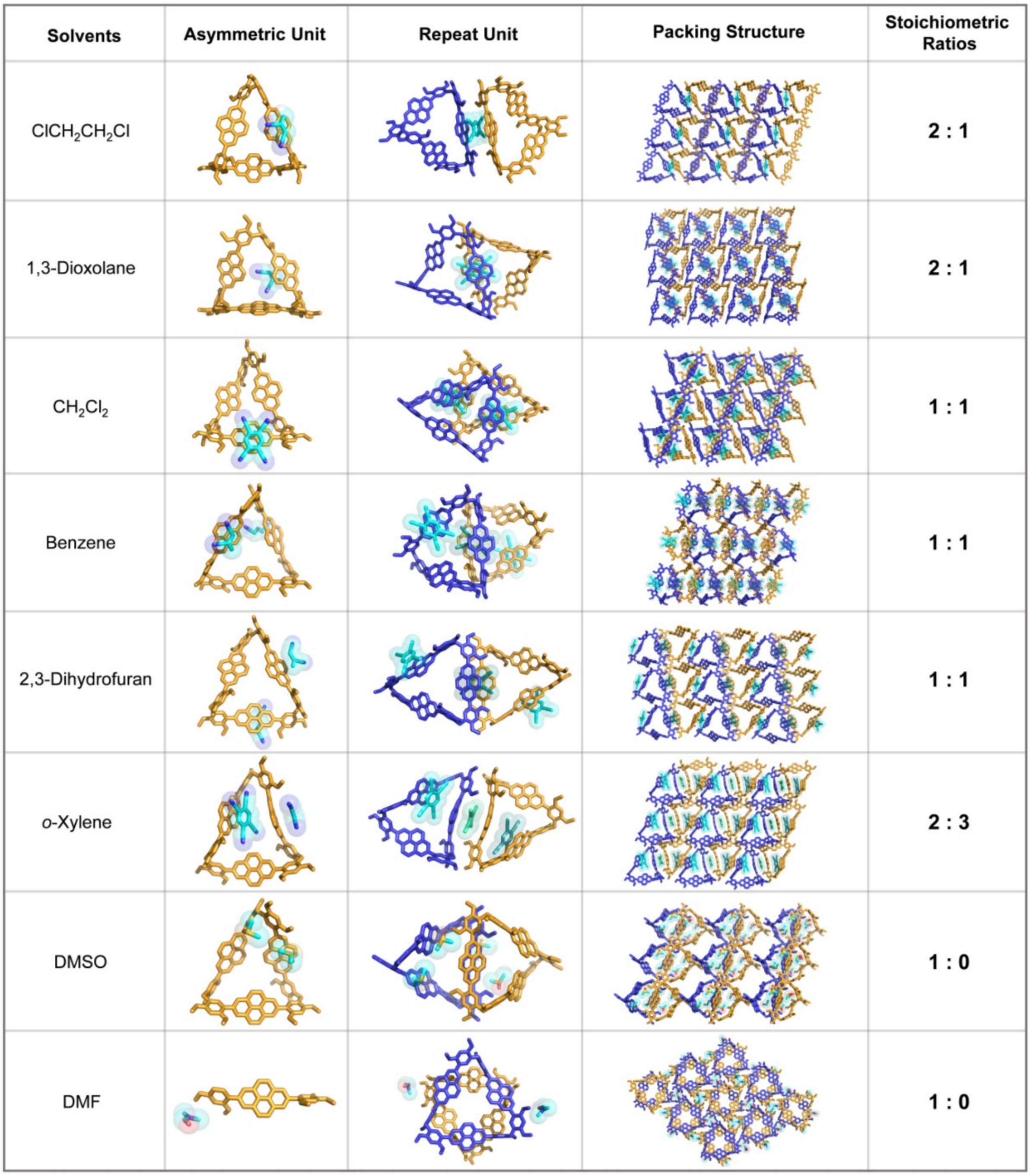

Supplementary Figure 44. The structures of MCCs with different D-A stoichiometric ratios (2:1, 1:1, 2:3 and 1:0) crystallized in different solvents. The results reveal that each packing structure differentiates from others, indicating the structural diversity of MCCs.

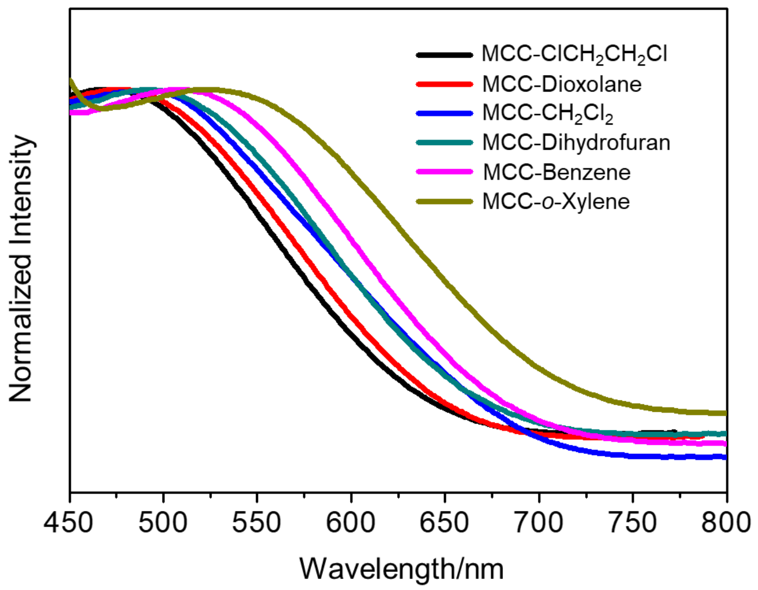

Supplementary Figure 45. Solid-state UV-Vis absorption spectra of MCC-$ClCH_2CH_2Cl$ (475 nm), MCC-Dioxolane (488 nm), MCC-CH_2Cl_2 (490 nm), MCC-Dihydrofuran (496 nm), MCC-Benzene (505 nm) and MCC-*o*-Xylene (537 nm).

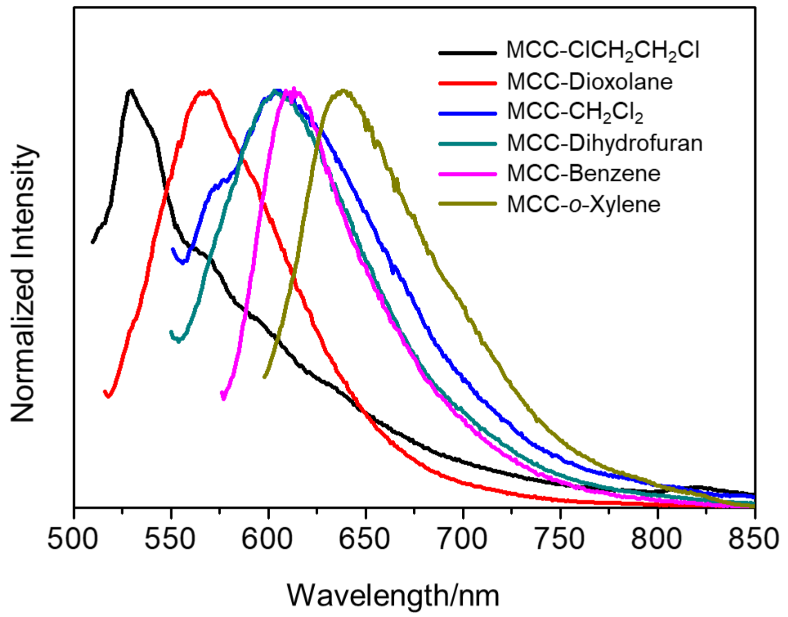

Supplementary Figure 46. Solid-state fluorescence spectra of MCC-$ClCH_2CH_2Cl$ (E_m: 527 nm), MCC-Dioxolane (E_m: 570 nm), MCC-CH_2Cl_2 (E_m: 605 nm), MCC-Dihydrofuran (E_m: 602 nm), MCC-Benzene (E_m: 613 nm) and MCC-*o*-Xylene (E_m: 637 nm).

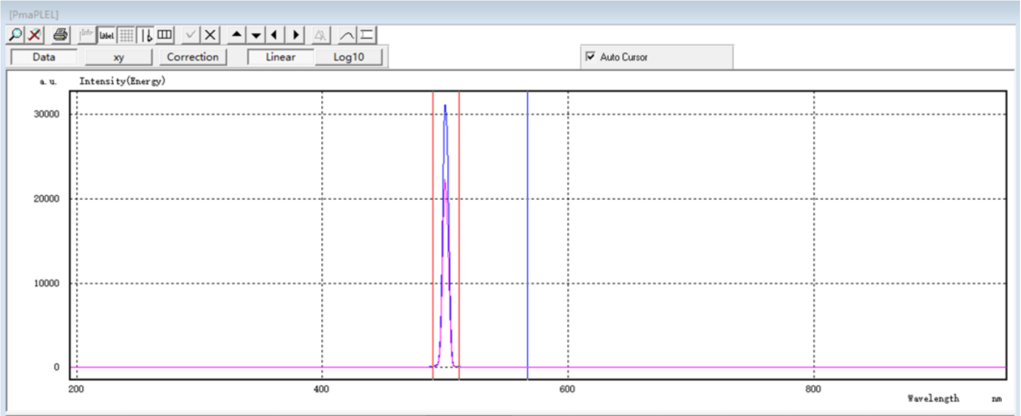

N o.	Cursor1-2: 489.77 - 510.78(...	Cursor3-4: 566.93 - 957.48(...	QuantumYield	Abs	Peak Wavele...	Peak Co...	Peak FW...	Peak Wavele...	Peak Co...	Peak FW...
MCC-ClCH2CH2Cl	86388057	---	---	---	500.28	31161.91	5.20	942.28	15.09	0.60
1	61118605	107348	0.004	0.293	499.53	22251.42	5.16	945.90	10.99	1.17
2	61152655	86228	0.003	0.292	499.53	22247.69	5.16	938.66	11.76	0.85
3	61161383	136757	0.005	0.292	499.53	22245.07	5.16	905.28	8.90	1.15

Supplementary Figure 47. Quantum yield of MCC-$ClCH_2CH_2Cl$.

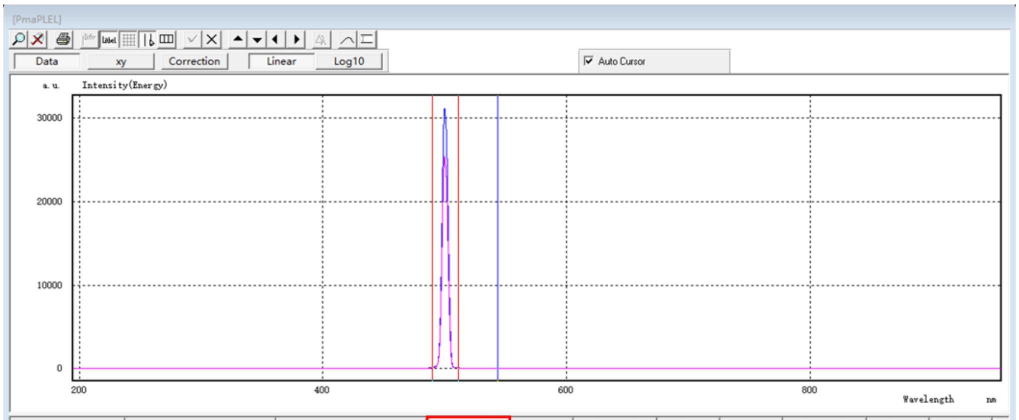

N o.	Cursor1-2: 489.77 - 510.78(...	Cursor3-4: 543.75 - 957.48(...	QuantumYield	Abs	Peak Wavele...	Peak Co...	Peak FW...	Peak Wavele...	Peak Co...	Peak FW...
MCC-Dioxolane	86388057	---	---	---	500.28	31161.91	5.20	942.28	15.09	0.60
1	69736119	252937	0.015	0.193	499.53	25354.30	5.15	938.66	9.45	1.39
2	69776185	244341	0.015	0.192	499.53	25381.20	5.14	949.52	10.26	0.45
3	69799264	204494	0.012	0.192	499.53	25381.70	5.14	950.97	10.93	0.96

Supplementary Figure 48. Quantum yield of MCC-Dioxolane.

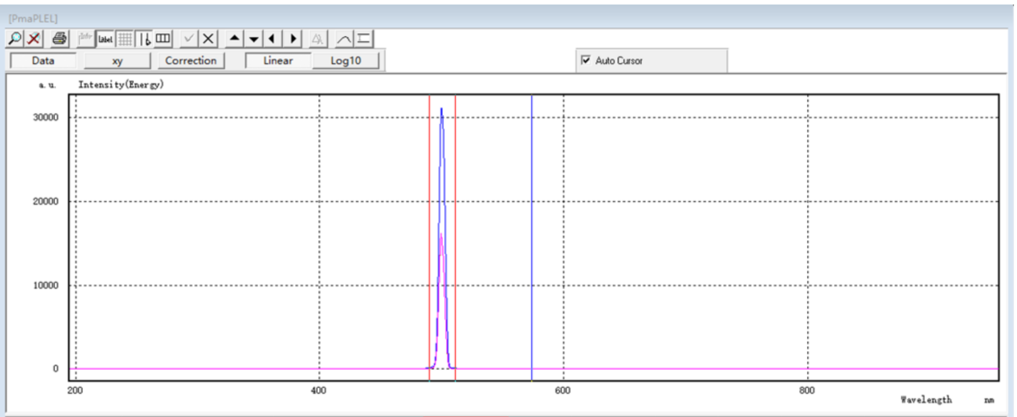

N o.	Cursor1-2: 489.77 - 510.78(...	Cursor3-4: 573.65 - 957.48(...	QuantumYield	Abs	Peak Wavele...	Peak Co...	Peak FW...	Peak Wavele...	Peak Co...	Peak FW...
MCC-CH2Cl2	86388057	---	---	---	500.28	31161.91	5.20	942.28	15.09	0.60
1	44014296	552174	0.013	0.491	499.53	16133.01	5.11	956.76	13.06	0.64
2	44036768	507288	0.012	0.490	499.53	16166.48	5.10	941.56	10.84	1.05
3	44064947	427003	0.010	0.490	499.53	16144.88	5.11	904.55	9.53	1.96

Supplementary Figure 49. Quantum yield of MCC-CH_2Cl_2.

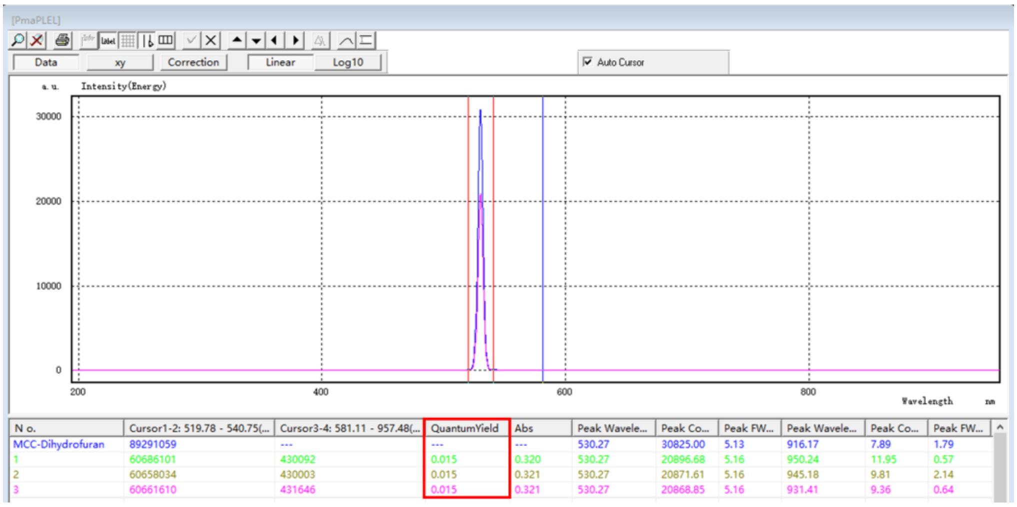

N o.	Cursor1-2: 519.78 - 540.75(...	Cursor3-4: 581.11 - 957.48(...	QuantumYield	Abs	Peak Wavele...	Peak Co...	Peak FW...	Peak Wavele...	Peak Co...	Peak FW...
MCC-Dihydrofuran	89291059	---	---	---	530.27	30825.00	5.13	916.17	7.89	1.79
1	60686101	430092	0.015	0.320	530.27	20896.68	5.16	950.24	11.95	0.57
2	60658034	430003	0.015	0.321	530.27	20871.61	5.16	945.18	9.81	2.14
3	60661610	431646	0.015	0.321	530.27	20868.85	5.16	931.41	9.36	0.64

Supplementary Figure 50. Quantum yield of MCC-Dihydrofuran.

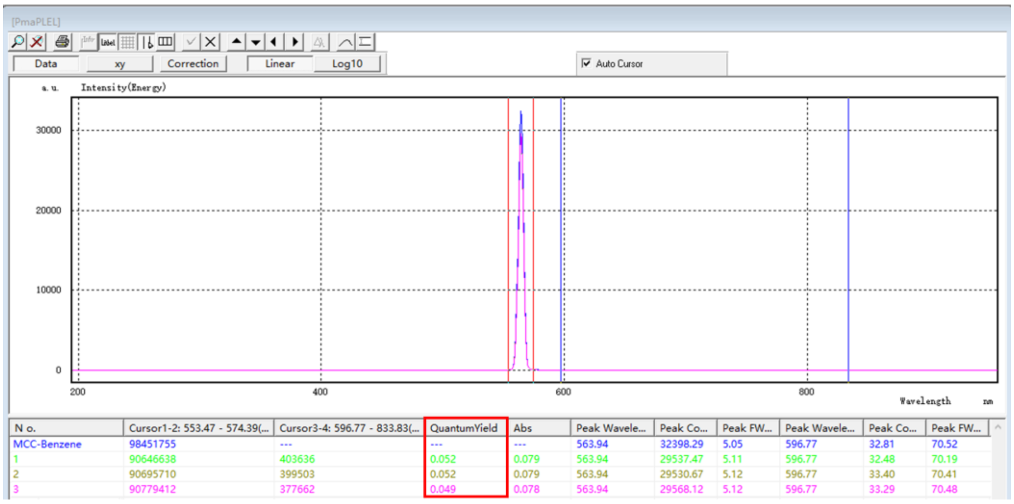

N o.	Cursor1-2: 553.47 - 574.39(...	Cursor3-4: 596.77 - 833.83(...	QuantumYield	Abs	Peak Wavele...	Peak Co...	Peak FW...	Peak Wavele...	Peak Co...	Peak FW...
MCC-Benzene	98451755	---	---	---	563.94	32398.29	5.05	596.77	32.81	70.52
1	90646638	403636	0.052	0.079	563.94	29537.47	5.11	596.77	32.48	70.19
2	90695710	399503	0.052	0.079	563.94	29530.67	5.12	596.77	33.40	70.41
3	90779412	377662	0.049	0.078	563.94	29568.12	5.12	596.77	33.29	70.48

Supplementary Figure 51. Quantum yield of MCC-Benzene.

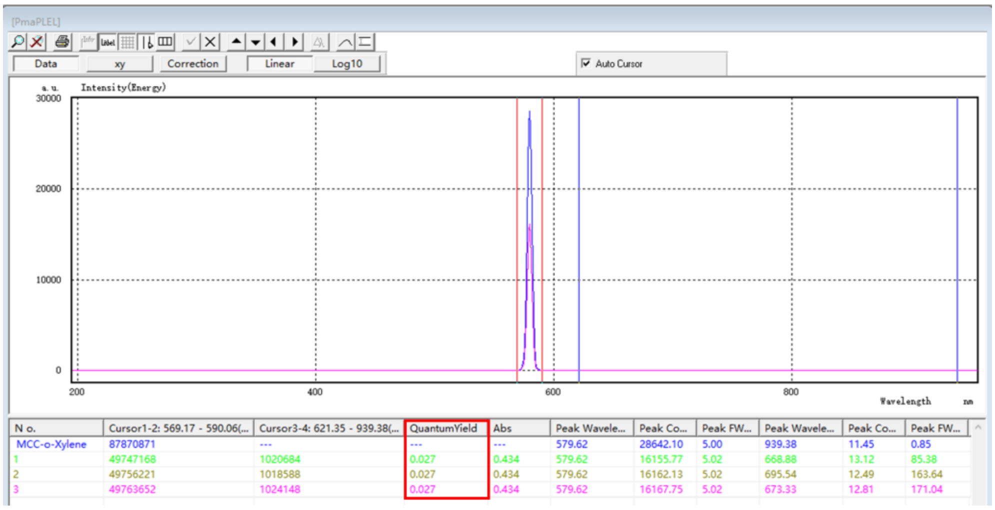

N o.	Cursor1-2: 569.17 - 590.06(...	Cursor3-4: 621.35 - 939.38(...	QuantumYield	Abs	Peak Wavele...	Peak Co...	Peak FW...	Peak Wavele...	Peak Co...	Peak FW...
MCC-o-Xylene	87870871	---	---	---	579.62	28642.10	5.00	939.38	11.45	0.85
1	49747168	1020684	0.027	0.434	579.62	16155.77	5.02	668.88	13.12	85.38
2	49756221	1018588	0.027	0.434	579.62	16162.13	5.02	695.54	12.49	163.64
3	49763652	1024148	0.027	0.434	579.62	16167.75	5.02	673.33	12.81	171.04

Supplementary Figure 52. Quantum yield of MCC-*o*-Xylene.

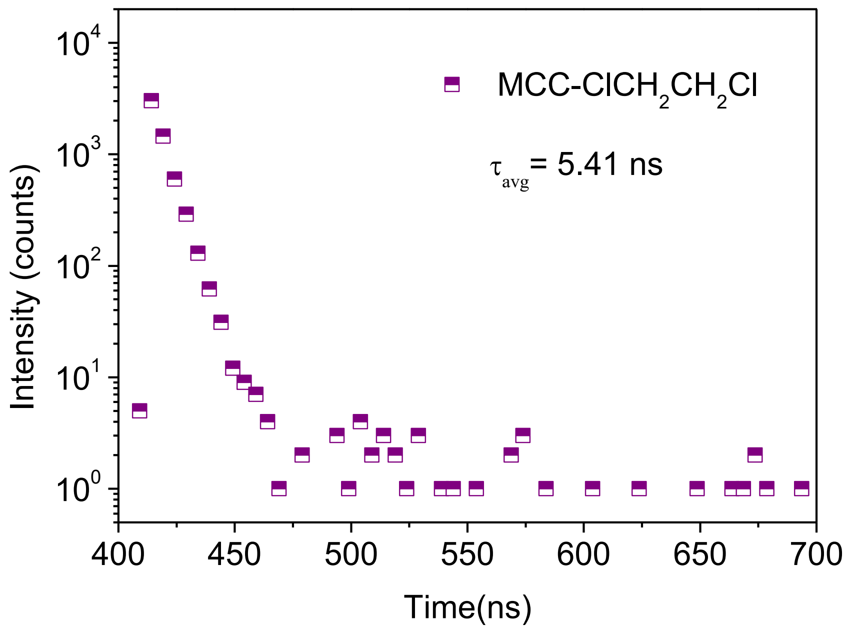

Supplementary Figure 53. Time-resolved fluorescence decay curve of MCC-$ClCH_2CH_2Cl$ at 527 nm in the solid state.

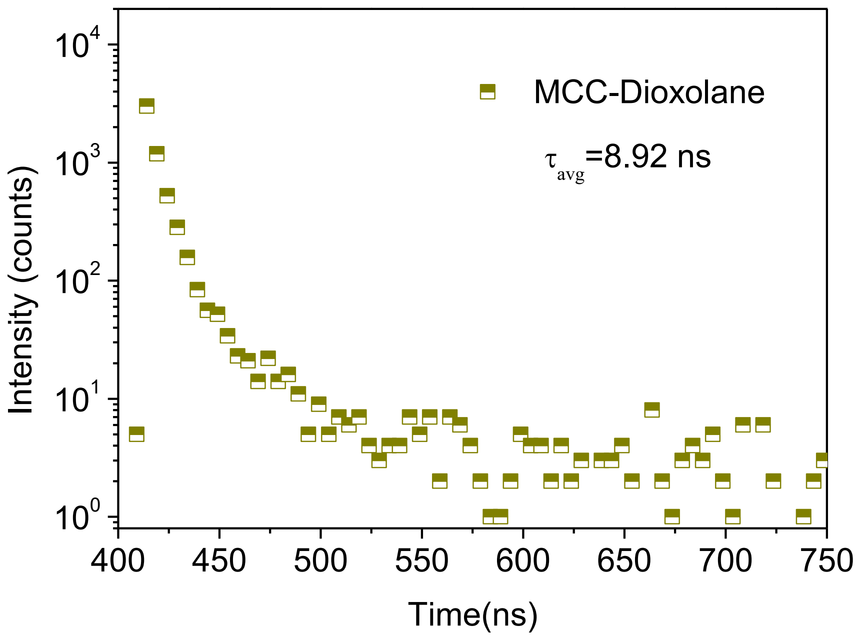

Supplementary Figure 54. Time-resolved fluorescence decay curve of MCC-Dioxolane at 570 nm in the solid state.

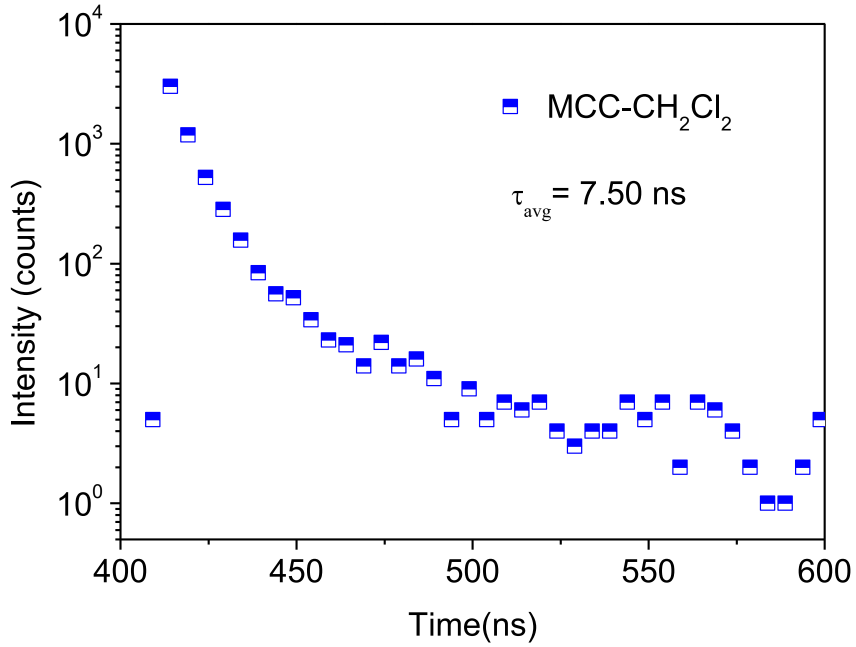

Supplementary Figure 55. Time-resolved fluorescence decay curve of MCC-CH_2Cl_2 at 605 nm in the solid state.

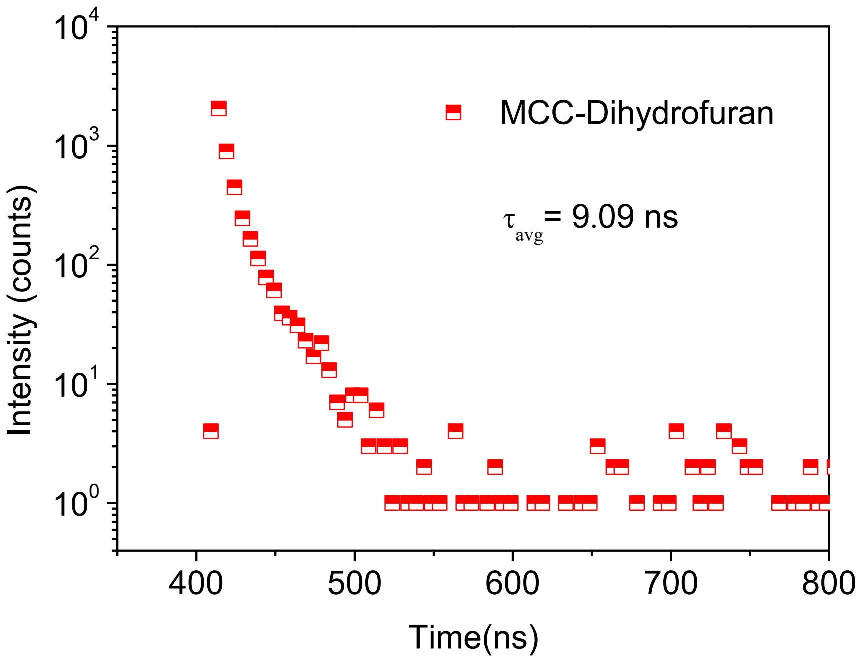

Supplementary Figure 56. Time-resolved fluorescence decay curve of MCC-Dihydrofuran at 602 nm in the solid state.

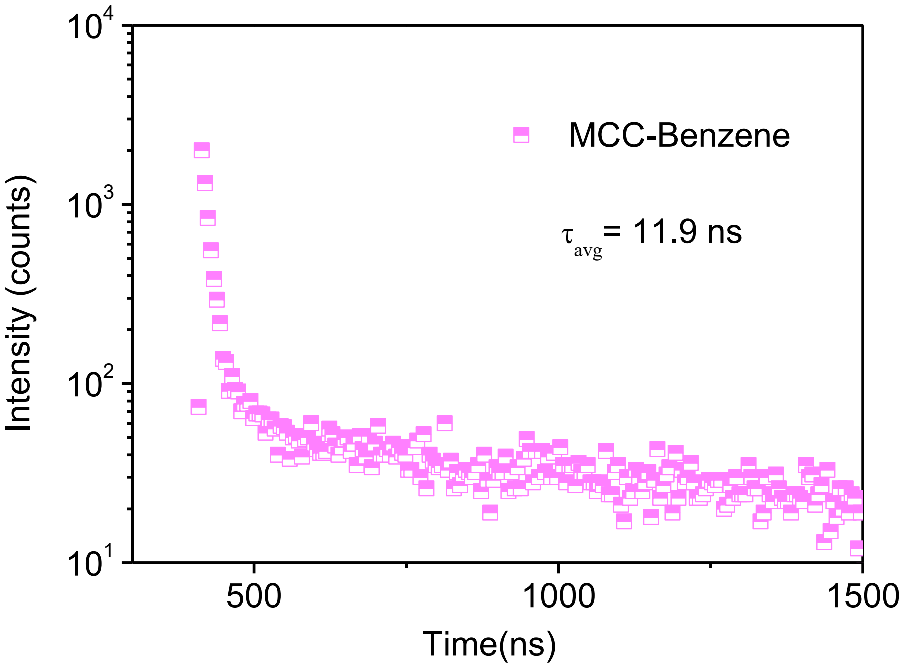

Supplementary Figure 57. Time-resolved fluorescence decay curve of MCC-Benzene at 613 nm in the solid state.

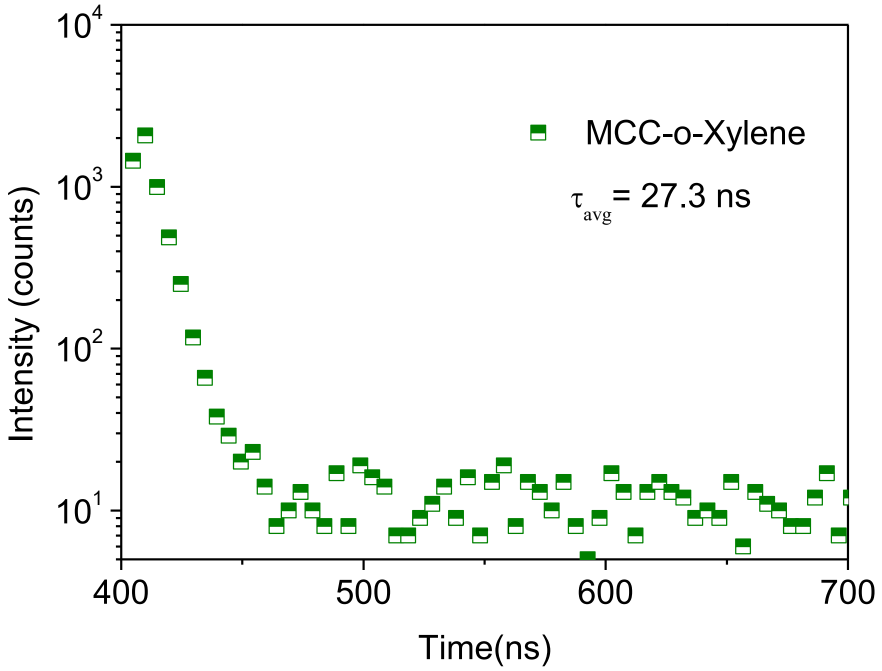

Supplementary Figure 58. Time-resolved fluorescence decay curve of MCC-*o*-Xylene at 637 nm in the solid state.

As shown in Supplementary Figures 45 and 46, the solid-state ultraviolet-visible (UV-vis) absorption and fluorescence spectra shown that the MCCs significantly red-shifted with the increase of CT participation ratio of macrocyclic skeleton (from 2:1 to 1:1 to 2:3). The solid-state PLQYs of MCC-$ClCH_2CH_2Cl$, MCC-Dioxolane, MCC-CH_2Cl_2, MCC-Dihydrofuran, MCC-Benzene and MCC-*o*-Xylene were 0.4, 1.4, 1.2, 1.5, 5.1 and 2.7%, respectively (Supplementary Figures 47–52). The average lifetimes of the 6 MCCs displayed an increasing tendency with the increase of CT participation ratio of macrocyclic skeleton (Supplementary Figures 53–58).

Supplementary Table 8. Experimental single crystal X-ray data for MCC-$ClCH_2CH_2Cl$ structure.

Formula	MCC-$ClCH_2CH_2Cl$
Crystallization Solvent	$ClCH_2CH_2Cl$
Formula	$C_{107}H_{81}Cl_3N_2O_{12}$
Formula weight	1693.08
Temperature / K	193
Crystal system	Triclinic
Space group	*P*-1
Unit cell dimensions	a = 13.9524(8) Å α = 102.009(3) °
	b = 17.1461(9) Å β = 92.482(3) °
	c = 22.2786(11) Å γ = 108.301(3) °
Volume / Å^3	4915.4(5)
Z	2
ρ_{calc} g/cm^3	1.144
μ / mm^{-1}	0.152
Crystal size / mm^3	0. 1 × 0.2 × 0.3
Radiation	Ga-*K*α (λ = 1.34138 Å)
F(000)	1768.0
2Θ range for data collection /°	1.882 to 50.932
Index ranges	-16 ≤ h ≤ 16, -20 ≤ k ≤ 20, -26 ≤ l ≤ 26
Reflections collected	57172
R_{int}	0.0732
Goodness-of-fit on F^2	1.056
Final R_1 indexes [I>= 2σ(I)]	0.0891
Final R_1 indexes [all data]	0.1475
Final wR(F_2) indexes [all data]	0.2872
Largest diff. peak/hole / eÅ^{-3}	0.69/-0.70
**CCDC number**	**2284505**

Supplementary Table 9. Experimental single crystal X-ray data for MCC-Dioxolane structure.

Formula	MCC-Dioxolane
Crystallization Solvent	1,3-Dioxolane
Formula	$C_{104}H_{79}N_2O_{12}$
Formula weight	1548.69
Temperature / K	193
Crystal system	triclinic
Space group	*P*-1
Unit cell dimensions	a = 18.7760(13) Å α = 89.489(4)°
	b = 18.9217(12) Å β = 64.269(3)°
	c = 19.6929(13) Å γ = 63.396(3)°
Volume / Å^3	5483.8(7)
Z	2
ρ_{calc} g/cm^3	0.938
μ / mm^{-1}	0.061
Crystal size / mm^3	0. 11 × 0.15 × 0.23
Radiation	Ga-*K*α (λ = 1.34138 Å)
F(000)	1626.0
2Θ range for data collection /°	2.360 to 50.880
Index ranges	-22 ≤ h ≤ 22, -22 ≤ k ≤ 22, -23 ≤ l ≤ 23
Reflections collected	67480
R_{int}	0.0588
Goodness-of-fit on F^2	1.076
Final R_1 indexes [I>= 2σ(I)]	0.1007
Final R_1 indexes [all data]	0.1343
Final wR(F_2) indexes [all data]	0.3326
Largest diff. peak/hole / eÅ^{-3}	0.44/-0.47
**CCDC number**	**2284851**

Supplementary Table 10. Experimental single crystal X-ray data for MCC-CH_2Cl_2 structure.

Formula	MCC-CH_2Cl_2	
Crystallization Solvent	CH_2Cl_2	
Formula	$C_{109}H_{80}N_4O_{12}$	
Formula weight	1637.77	
Temperature / K	193	
Crystal system	Triclinic	
Space group	*P*-1	
Unit cell dimensions	a = 14.8449(6) Å	α = 71.611(2)°
	b = 18.0738(8)Å	β = 72.804(2)°
	c = 22.7299(9) Å	γ = 72.559(2)°
Volume / $Å^3$	5383.1(4)	
Z	2	
ρ_{calc} g/cm^3	1.010	
μ / mm^{-1}	0.066	
Crystal size / mm^3	0. 1 × 0.2 × 0.3	
Radiation	Ga-*K*α (λ = 1.34138 Å)	
F(000)	1716.0	
2Θ range for data collection /°	1.936 to 50.890	
Index ranges	-17 ≤ h ≤ 17, -21 ≤ k ≤ 17, -27 ≤ l ≤ 27	
Reflections collected	59217	
R_{int}	0.0613	
Goodness-of-fit on F^2	1.041	
Final R_1 indexes [I>= 2σ(I)]	0.0873	
Final R_1 indexes [all data]	0.1356	
Final wR(F_2) indexes [all data]	0.3038	
Largest diff. peak/hole / eÅ^{-3}	0.31/-0.26	
**CCDC number**	**2284533**	

Supplementary Table 11. Experimental single crystal X-ray data for MCC-Benzene structure.

Formula	MCC-Benzene	
Crystallization Solvent	Benzene	
Formula	$C_{169}H_{140}N_4O_{12}$	
Formula weight	2418.84	
Temperature / K	193	
Crystal system	Triclinic	
Space group	*P*-1	
Unit cell dimensions	a = 19.5831(9) Å	α = 91.932(2)°
	b = 20.0799(10) Å	β = 117.409(2)°
	c = 20.5891(10) Å	γ = 99.289(2)°
Volume / Å^3	7039.0(6)	
Z	2	
ρ_{calc} g/cm^3	1.141	
μ / mm^{-1}	0.071	
Crystal size / mm^3	0. 1 × 0.2 × 0.3	
Radiation	Ga-*K*α (λ = 1.34138 Å)	
F(000)	2556.0	
2Θ range for data collection /°	2.070 to 50.824	
Index ranges	-23 ≤ h ≤ 23, -24 ≤ k ≤ 24, -24 ≤ l ≤ 24	
Reflections collected	85210	
R_{int}	0.0413	
Goodness-of-fit on F^2	1.041	
Final R_1 indexes [I>= 2σ(I)]	0.0652	
Final R_1 indexes [all data]	0.0929	
Final wR(F_2) indexes [all data]	0.2003	
Largest diff. peak/hole / eÅ^{-3}	0.55/-0.50	
**CCDC number**	**2284548**	

Supplementary Table 12. Experimental single crystal X-ray data for MCC-Dihydrofuran structure.

Formula	MCC-Dihydrofuran
Crystallization Solvent	2,3-Dihydrofuran
Formula	$C_{109}H_{80}N_4O_{12}$
Formula weight	1637.77
Temperature / K	193
Crystal system	Triclinic
Space group	*P*-1
Unit cell dimensions	a = 16.5277(5) Å α = 73.019(1)°
	b = 18.1761(5) Å β = 87.205(2)°
	c = 24.7793(8) Å γ = 81.333(2)°
Volume / Å^3	7038.0(4)
Z	2
ρ_{calc} g/cm^3	0.773
μ / mm^{-1}	0.050
Crystal size / mm^3	0. 1 × 0.2 × 0.3
Radiation	Ga-*K*α (λ = 1.34138 Å)
F(000)	1716.0
2Θ range for data collection /°	1.718 to 50.820
Index ranges	-19 ≤ h ≤ 19, -21 ≤ k ≤ 21, -29 ≤ l ≤ 29
Reflections collected	79708
R_{int}	0.0628
Goodness-of-fit on F^2	1.016
Final R_1 indexes [I>= 2σ(I)]	0.0904
Final R_1 indexes [all data]	0.1389
Final wR(F_2) indexes [all data]	0.3300
Largest diff. peak/hole / eÅ^{-3}	0.29/-0.29
**CCDC number**	**2284568**

Supplementary Table 13. Experimental single crystal X-ray data for MCC-*o*-Xylene structure.

Formula	MCC-*o*-Xylene	
Crystallization Solvent	*o*-Xylene	
Formula	$C_{154}H_{131}N_6O_{12}$	
Formula weight	2257.64	
Temperature / K	193	
Crystal system	Triclinic	
Space group	*P*-1	
Unit cell dimensions	a = 15.6338(10) Å	α = 79.798(3)°
	b = 18.6013(11)Å	β = 72.410(4)°
	c = 23.9689(16) Å	γ = 69.806(3)°
Volume / Å^3	6215.3(7)	
Z	2	
ρ_{calc} g/cm^3	1.206	
μ / mm^{-1}	0.076	
Crystal size / mm^3	0. 1 × 0.2 × 0.3	
Radiation	Ga-*K*α (λ = 1.34138 Å)	
F(000)	2386.0	
2Θ range for data collection /°	1.788 to 50.998	
Index ranges	-18 ≤ h ≤ 18, -22 ≤ k ≤ 22, -28 ≤ l ≤ 28	
Reflections collected	72653	
R_{int}	0.0631	
Goodness-of-fit on F^2	0.989	
Final R_1 indexes [I>= 2σ(I)]	0.1180	
Final R_1 indexes [all data]	0.1636	
Final wR(F_2) indexes [all data]	0.3973	
Largest diff. peak/hole / eÅ^{-3}	0.58/-0.41	
**CCDC number**	**2284821**	

Supplementary Table 14. Experimental single crystal X-ray data for Pe[3]-DMSO structure.

Formula	Pe[3]-DMSO	
Crystallization Solvent	DMSO	
Formula	$C_{103}H_{90}O_{14}S_2$	
Formula weight	1615.86	
Temperature / K	193	
Crystal system	triclinic	
Space group	*P*-1	
Unit cell dimensions	a = 13.9873(8) Å	α = 73.354(2)°
	b = 17.1586(11) Å	β = 77.262(2)°
	c = 25.3581(16) Å	γ = 71.157(2)°
Volume / $Å^3$	5463.2(6)	
Z	2	
ρ_{calc} g/cm^3	0.982	
μ / mm^{-1}	0.101	
Crystal size / mm^3	0. 1 × 0.2 × 0.3	
Radiation	Mo-Kα (λ = 0.71073 Å)	
F(000)	1704.0	
2Θ range for data collection /°	3.744 to 55.056	
Index ranges	$-18 \le h \le 18$, $-22 \le k \le 22$, $-24 \le l \le 32$	
Reflections collected	51281	
R_{int}	0.0469	
Goodness-of-fit on F^2	1.037	
Final R_1 indexes [I>= 2σ(I)]	0.0829	
Final R_1 indexes [all data]	0.1237	
Final wR(F_2) indexes [all data]	0.2842	
Largest diff. peak/hole / eÅ^{-3}	0.66/-0.46	
**CCDC number**	**2285102**	

Supplementary Table 15. Experimental single crystal X-ray data for Pe[3]-DMF structure.

Formula	Pe[3]-DMF
Crystallization Solvent	DMF
Formula	$C_{108}H_{99}N_3O_{15}$
Formula weight	1678.90
Temperature / K	193
Crystal system	trigonal
Space group	*P*-3
Unit cell dimensions	a = 38.5418(18) Å α = 90°
	b = 38.5418(18) Å β = 90°
	c = 11.7183(6) Å γ = 120°
Volume / $Å^3$	15075.1(16)
Z	6
ρ_{calc} g/cm^3	1.110
μ / mm^{-1}	0.074
Crystal size / mm^3	0. 1 × 0.2 × 0.3
Radiation	Mo-Kα (λ = 0.71073 Å)
F(000)	5328.0
2Θ range for data collection /°	3.684 to 55.208
Index ranges	-50 ≤ h ≤ 49, -50 ≤ k ≤ 50, -12 ≤ l ≤ 15
Reflections collected	47532
R_{int}	0.0565
Goodness-of-fit on F^2	1.039
Final R_1 indexes [I>= 2σ(I)]	0.0614
Final R_1 indexes [all data]	0.0816
Final wR(F_2) indexes [all data]	0.1833
Largest diff. peak/hole / eÅ^{-3}	0.66/-0.27
**CCDC number**	**2284673**

3. Supplementary Reference

1. Xu, K. et al. A Modular Synthetic Strategy for Functional Macrocycles. *Angew. Chem. Int. Ed.* **59**, 7214–7218 (2020).
2. Sheldrick, G. M. Crystal structure refinement with SHELXL. *Acta Crystallogr. C*. **71**, 3–8 (2015).
3. Dolomanov, O. V., Bourhis, L. J., Gildea, R. J., Howard, J. A. K. & Puschmann, H. *OLEX2:* a complete structure solution, refinement and analysis program. *J. Appl. Cryst.* **42**, 339–341 (2009).
4. M. Frisch, G. Trucks, H. Schlegel, G. Scuseria, M. Robb, J. Cheeseman, G. Scalmani, V. Barone, G. Petersson, H. Nakatsuji, Gaussian 16, revision B. 01; Gaussian: Wallingford, CT, 2016.
5. 55. Lu, T. & Chen, F. Multiwfn: A multifunctional wavefunction analyzer. *J. Comput. Chem.* **33**, 580–592 (2012).
