# Supplementary material for: Structurally diverse macrocycle co-crystals for solid-state luminescence modulation
Source: Nat Commun. 2024 Mar 21;15:2535. doi: 10.1038/s41467-024-46788-6 (PMC10957888; doi:10.1038/s41467-024-46788-6)
Supplement: Supplementary file 3 — Description of Additional Supplementary Files [file 41467_2024_46788_MOESM3_ESM.pdf]

Description of Additional Supplementary Files

File Name: Supplementary Data 1
Description: The atomic coordinates of the optimized computational models in the manuscript.
