# Supplementary material for: Structurally diverse macrocycle co-crystals for solid-state luminescence modulation
Source: Nat Commun. 2024 Mar 21;15:2535. doi: 10.1038/s41467-024-46788-6 (PMC10957888; doi:10.1038/s41467-024-46788-6)

checkCIF/PLATON report

Structure factors have been supplied for datablock(s) a_sq

THIS REPORT IS FOR GUIDANCE ONLY. IF USED AS PART OF A REVIEW PROCEDURE FOR PUBLICATION, IT SHOULD NOT REPLACE THE EXPERTISE OF AN EXPERIENCED CRYSTALLOGRAPHIC REFEREE.

No syntax errors found.   CIF dictionary   Interpreting this report

Datablock: a_sq

Bond precision: C-C = 0.0150 A   Wavelength=0.71073

Cell:	a=17.933(3)	b=19.019(3)	c=23.176(4)
	alpha=72.498(5)	beta=70.940(4)	gamma=75.284(5)
Temperature:	120 K		

	Calculated	Reported
Volume	7018(2)	7017.8(18)
Space group	P -1	P -1
Hall group	-P 1	-P 1
Moiety formula	C99 H78 O12, 1.5(C10 H2 N4), 7(C H Cl3) [+ solvent]	7(C H Cl3), C99 H78 O12, 1.5(C10 H2 N4)
Sum formula	C121 H88 Cl21 N6 O12 [+ solvent]	C121 H88 Cl21 N6 O12
Mr	2562.43	2562.42
Dx,g cm-3	1.213	1.213
Z	2	2
Mu (mm-1)	0.461	0.461
F000	2618.0	2618.0
F000'	2625.11	
h,k,lmax	18,19,23	17,19,23
Nref	14881	14817
Tmin,Tmax	0.915,0.946	0.535,0.745
Tmin'	0.912	

Correction method= # Reported T Limits: Tmin=0.535 Tmax=0.745
AbsCorr = MULTI-SCAN

Data completeness= 0.996   Theta(max)= 20.906

R(reflections)= 0.1292(12158)   wR2(reflections)= 0.3453(14817)

S = 1.058   Npar= 1453

The following ALERTS were generated. Each ALERT has the format
test-name_ALERT_alert-type_alert-level.
Click on the hyperlinks for more details of the test.

Alert level A

THETM01_ALERT_3_A The value of sine(theta_max)/wavelength is less than 0.550
Calculated sin(theta_max)/wavelength = 0.5021

Author Response: The crystals were weakly diffracting at high angle which is not uncomm for structures of this type.

Alert level B

PLAT097_ALERT_2_B Large Reported Max. (Positive) Residual Density 2.45 eA-3

Author Response: This could be a minor effect of the disorder.

PLAT260_ALERT_2_B Large Average Ueq of Residue Including Cl19 0.416 Check

Author Response: Disordered solvent molecules.

PLAT340_ALERT_3_B Low Bond Precision on C-C Bonds .... 0.01503 Ang.

Author Response: This is attributed to disorder present in the structure.

PLAT971_ALERT_2_B Check Calcd Resid. Dens. 1.80Ang From Cl21 2.53 eA-3

Author Response: Due to the severe disorder of the CHCl3 solvent.

Alert level C

DIFMX02_ALERT_1_C The maximum difference density is > 0.1*ZMAX*0.75
The relevant atom site should be identified.
PLAT082_ALERT_2_C High R1 Value .... 0.13 Report
PLAT084_ALERT_3_C High wR2 Value (i.e. > 0.25) .... 0.35 Report
PLAT094_ALERT_2_C Ratio of Maximum / Minimum Residual Density .... 2.27 Report
PLAT220_ALERT_2_C NonSolvent Resd 1 C Ueq(max)/Ueq(min) Range 3.5 Ratio
PLAT244_ALERT_4_C Low 'Solvent' Ueq as Compared to Neighbors of C120 Check

```
PLAT244_ALERT_4_C Low    'Solvent' Ueq as Compared to Neighbors of       C122 Check
PLAT244_ALERT_4_C Low    'Solvent' Ueq as Compared to Neighbors of       C123 Check
PLAT250_ALERT_2_C Large U3/U1 Ratio for Average U(i,j) Tensor ....        3.0 Note
PLAT260_ALERT_2_C Large Average Ueq of Residue Including       Cl7      0.152 Check
```

Author Response: Disordered solvent molecules.

```
PLAT260_ALERT_2_C Large Average Ueq of Residue Including       Cl8      0.133 Check
```

Author Response: Disordered solvent molecules.

```
PLAT260_ALERT_2_C Large Average Ueq of Residue Including       Cl9      0.149 Check
```

Author Response: Disordered solvent molecules.

```
PLAT260_ALERT_2_C Large Average Ueq of Residue Including      Cl10      0.115 Check
```

Author Response: Disordered solvent molecules.

```
PLAT336_ALERT_2_C Long Bond Distance for ..... C125     -Cl18           1.860 Ang.
PLAT336_ALERT_2_C Long Bond Distance for ..... C124     -Cl15           1.910 Ang.
PLAT411_ALERT_2_C Short Inter H...H Contact  H78      ..H126     .       2.04 Ang.
                                              x,1+y,z  =      1_565 Check
PLAT906_ALERT_3_C Large K Value in the Analysis of Variance ......     11.345 Check
PLAT906_ALERT_3_C Large K Value in the Analysis of Variance ......      2.419 Check
PLAT910_ALERT_3_C Missing # of FCF Reflection(s) Below Theta(Min).         10 Note
PLAT911_ALERT_3_C Missing FCF Refl Between Thmin & STh/L=    0.502         52 Report
PLAT918_ALERT_3_C Reflection(s) with I(obs) much Smaller I(calc) .          1 Check
PLAT921_ALERT_1_C R1  in the CIF and FCF Differ by ..............     0.0012 Check
PLAT922_ALERT_1_C wR2 in the CIF and FCF Differ by ..............     0.0036 Check
PLAT923_ALERT_1_C S    Values in the CIF and FCF Differ by .......      0.013 Check
PLAT971_ALERT_2_C Check Calcd Resid. Dens.  1.18Ang From Cl21            2.37 eA-3
```

Author Response: Due to the severe disorder of the CHCl3 solvent.

```
PLAT971_ALERT_2_C Check Calcd Resid. Dens.  1.43Ang From C126            1.70 eA-3
```

Author Response: Due to the severe disorder of the CHCl3 solvent.

```
PLAT977_ALERT_2_C Check Negative Difference Density on H126      .      -0.39 eA-3
```

Alert level G

```
PLAT002_ALERT_2_G Number of Distance or Angle Restraints on AtSite          6 Note
PLAT003_ALERT_2_G Number of Uiso or Uij Restrained non-H Atoms ...        160 Report
PLAT042_ALERT_1_G Calc. and Reported MoietyFormula Strings  Differ     Please Check
PLAT072_ALERT_2_G SHELXL First  Parameter in WGHT  Unusually Large       0.15 Report
PLAT083_ALERT_2_G SHELXL Second Parameter in WGHT  Unusually Large      94.88 Why ?
PLAT152_ALERT_1_G The Supplied and Calc. Volume s.u. Differ by ...          2 Units
```

```
PLAT172_ALERT_4_G The CIF-Embedded .res File Contains DFIX Records          4 Report
PLAT176_ALERT_4_G The CIF-Embedded .res File Contains SADI Records          2 Report
PLAT178_ALERT_4_G The CIF-Embedded .res File Contains SIMU Records          1 Report
PLAT187_ALERT_4_G The CIF-Embedded .res File Contains RIGU Records          1 Report
PLAT431_ALERT_2_G Short Inter HL..A Contact  Cl14      ..O10      .       3.02 Ang.
                                                   x,y,z  =      1_555 Check
PLAT606_ALERT_4_G Solvent Accessible VOID(S) in Structure ........          ! Info
PLAT720_ALERT_4_G Number of Unusual/Non-Standard Labels ..........          6 Note
PLAT790_ALERT_4_G Centre of Gravity not Within Unit Cell: Resd.  #          5 Note
              C H Cl3
PLAT790_ALERT_4_G Centre of Gravity not Within Unit Cell: Resd.  #          6 Note
              C H Cl3
PLAT790_ALERT_4_G Centre of Gravity not Within Unit Cell: Resd.  #          9 Note
              C H Cl3
PLAT790_ALERT_4_G Centre of Gravity not Within Unit Cell: Resd.  #         10 Note
              C H Cl3
PLAT802_ALERT_4_G CIF Input Record(s) with more than 80 Characters          1 Info
PLAT860_ALERT_3_G Number of Least-Squares Restraints .............       2258 Note
PLAT869_ALERT_4_G ALERTS Related to the Use of SQUEEZE  Suppressed          ! Info
PLAT909_ALERT_3_G Percentage of I>2sig(I) Data at Theta(Max) Still        58% Note
PLAT978_ALERT_2_G Number C-C Bonds with Positive Residual Density.          0 Info
```

```
  1 ALERT level A = Most likely a serious problem - resolve or explain
  4 ALERT level B = A potentially serious problem, consider carefully
 27 ALERT level C = Check. Ensure it is not caused by an omission or oversight
 22 ALERT level G = General information/check it is not something unexpected

  6 ALERT type 1 CIF construction/syntax error, inconsistent or missing data
 23 ALERT type 2 Indicator that the structure model may be wrong or deficient
 10 ALERT type 3 Indicator that the structure quality may be low
 15 ALERT type 4 Improvement, methodology, query or suggestion
  0 ALERT type 5 Informative message, check
```

It is advisable to attempt to resolve as many as possible of the alerts in all categories. Often the minor alerts point to easily fixed oversights, errors and omissions in your CIF or refinement strategy, so attention to these fine details can be worthwhile. In order to resolve some of the more serious problems it may be necessary to carry out additional measurements or structure refinements. However, the purpose of your study may justify the reported deviations and the more serious of these should normally be commented upon in the discussion or experimental section of a paper or in the "special_details" fields of the CIF. checkCIF was carefully designed to identify outliers and unusual parameters, but every test has its limitations and alerts that are not important in a particular case may appear. Conversely, the absence of alerts does not guarantee there are no aspects of the results needing attention. It is up to the individual to critically assess their own results and, if necessary, seek expert advice.

Publication of your CIF in IUCr journals

A basic structural check has been run on your CIF. These basic checks will be run on all CIFs submitted for publication in IUCr journals (*Acta Crystallographica*, *Journal of Applied Crystallography*, *Journal of Synchrotron Radiation*); however, if you intend to submit to *Acta Crystallographica Section C* or *E* or *IUCrData*, you should make sure that full publication checks are run on the final version of your CIF prior to submission.

Publication of your CIF in other journals

Please refer to the *Notes for Authors* of the relevant journal for any special instructions relating to CIF submission.

PLATON version of 28/11/2022; check.def file version of 28/11/2022

Datablock a_sq - ellipsoid plot

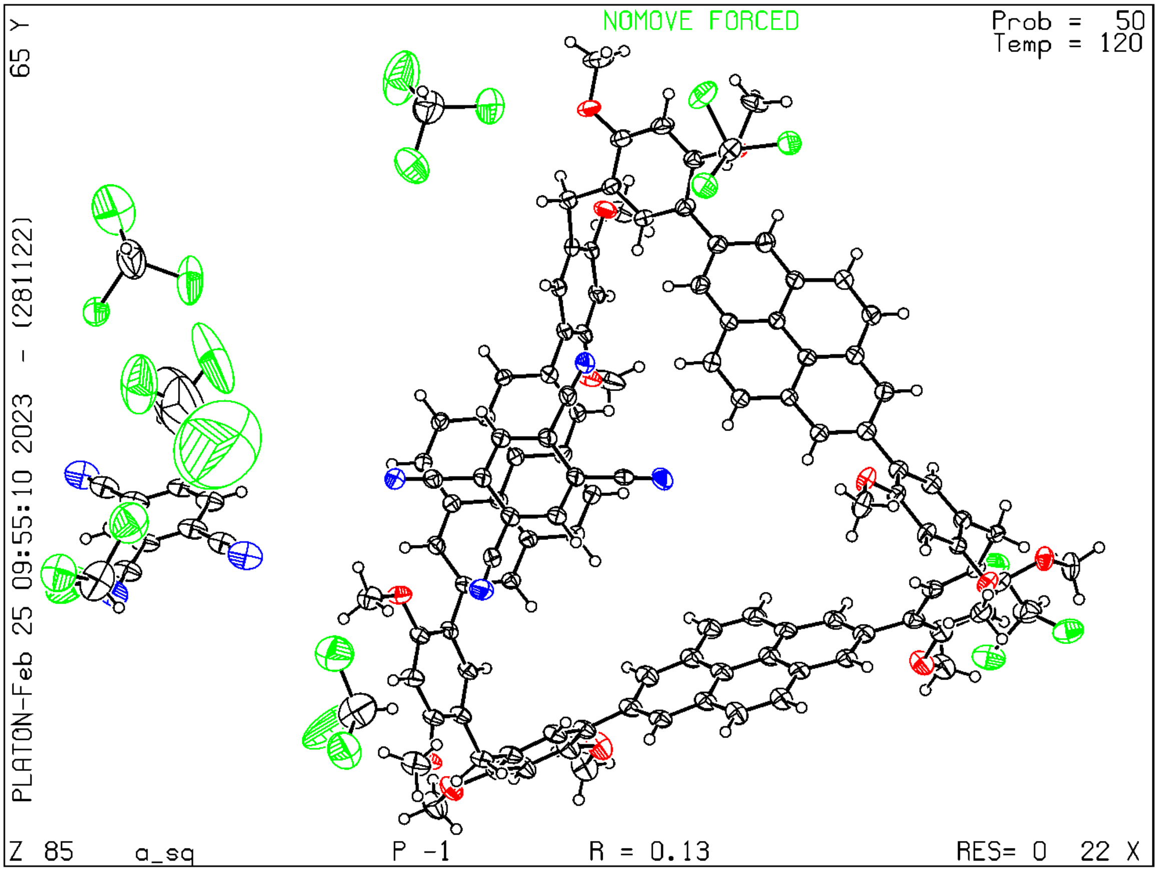

Supplement: Supplementary file 5 — Source Data [file 41467_2024_46788_MOESM5_ESM.zip › Single-crystal structures/MCC-3-checkcif.pdf]
