# Supplementary material for: Structurally diverse macrocycle co-crystals for solid-state luminescence modulation
Source: Nat Commun. 2024 Mar 21;15:2535. doi: 10.1038/s41467-024-46788-6 (PMC10957888; doi:10.1038/s41467-024-46788-6)

checkCIF/PLATON report

Structure factors have been supplied for datablock(s) 1

THIS REPORT IS FOR GUIDANCE ONLY. IF USED AS PART OF A REVIEW PROCEDURE FOR PUBLICATION, IT SHOULD NOT REPLACE THE EXPERTISE OF AN EXPERIENCED CRYSTALLOGRAPHIC REFEREE.

No syntax errors found. CIF dictionary Interpreting this report

Datablock: 1

Bond precision:	C-C = 0.0049 A		Wavelength=0.71073
Cell:	a=19.5831(9)	b=20.0799(10)	c=20.5891(10)
	alpha=91.932(2)	beta=117.409(2)	gamma=99.289(2)
Temperature:	193 K		

	Calculated	Reported
Volume	7039.0(6)	7039.0(6)
Space group	P -1	P -1
Hall group	-P 1	-P 1
Moiety formula	C99 H78 O12, C10 H2 N4, 10(C6 H6) [+ solvent]	C99 H78 O12, 2(C5 H N2), 10(C6 H6), 0.48[1C4O2]
Sum formula	C169 H140 N4 O12 [+ solvent]	C169 H140 N4 O12
Mr	2418.85	2418.84
Dx,g cm-3	1.141	1.141
Z	2	2
Mu (mm-1)	0.071	0.071
F000	2556.0	2556.0
F000'	2557.08	
h,k,lmax	23,24,24	23,24,24
Nref	25961	25811
Tmin,Tmax	0.983,0.993	0.664,0.751
Tmin'	0.979	

Correction method= # Reported T Limits: Tmin=0.664 Tmax=0.751
AbsCorr = NONE

Data completeness= 0.994 Theta(max)= 25.412

R(reflections)= 0.0649(17709) wR2(reflections)= 0.1993(25811)

S = 1.031 Npar= 1606

The following ALERTS were generated. Each ALERT has the format
test-name_ALERT_alert-type_alert-level.
Click on the hyperlinks for more details of the test.

Alert level C

```
PLAT243_ALERT_4_C High   'Solvent' Ueq as Compared to Neighbors of         C1 Check
PLAT243_ALERT_4_C High   'Solvent' Ueq as Compared to Neighbors of        C20 Check
PLAT243_ALERT_4_C High   'Solvent' Ueq as Compared to Neighbors of       C147 Check
PLAT243_ALERT_4_C High   'Solvent' Ueq as Compared to Neighbors of       C153 Check
PLAT243_ALERT_4_C High   'Solvent' Ueq as Compared to Neighbors of       C161 Check
PLAT244_ALERT_4_C Low    'Solvent' Ueq as Compared to Neighbors of         C3 Check
PLAT244_ALERT_4_C Low    'Solvent' Ueq as Compared to Neighbors of         C4 Check
PLAT244_ALERT_4_C Low    'Solvent' Ueq as Compared to Neighbors of        C22 Check
PLAT244_ALERT_4_C Low    'Solvent' Ueq as Compared to Neighbors of       C150 Check
PLAT244_ALERT_4_C Low    'Solvent' Ueq as Compared to Neighbors of       C154 Check
PLAT244_ALERT_4_C Low    'Solvent' Ueq as Compared to Neighbors of       C169 Check
PLAT250_ALERT_2_C Large U3/U1 Ratio for Average U(i,j) Tensor ....        2.1 Note
PLAT250_ALERT_2_C Large U3/U1 Ratio for Average U(i,j) Tensor ....        3.7 Note
PLAT250_ALERT_2_C Large U3/U1 Ratio for Average U(i,j) Tensor ....        2.7 Note
PLAT250_ALERT_2_C Large U3/U1 Ratio for Average U(i,j) Tensor ....        2.2 Note
PLAT250_ALERT_2_C Large U3/U1 Ratio for Average U(i,j) Tensor ....        2.3 Note
PLAT250_ALERT_2_C Large U3/U1 Ratio for Average U(i,j) Tensor ....        2.1 Note
PLAT260_ALERT_2_C Large Average Ueq of Residue Including        C1      0.192 Check
PLAT260_ALERT_2_C Large Average Ueq of Residue Including       C13      0.108 Check
PLAT260_ALERT_2_C Large Average Ueq of Residue Including       C25      0.110 Check
PLAT260_ALERT_2_C Large Average Ueq of Residue Including      C144      0.165 Check
PLAT260_ALERT_2_C Large Average Ueq of Residue Including      C152      0.175 Check
PLAT260_ALERT_2_C Large Average Ueq of Residue Including      C158      0.114 Check
PLAT260_ALERT_2_C Large Average Ueq of Residue Including      C164      0.156 Check
PLAT331_ALERT_2_C Small Aver Phenyl C-C Dist C8       --C151      .       1.36 Ang.
PLAT331_ALERT_2_C Small Aver Phenyl C-C Dist C19      --C24       .       1.37 Ang.
PLAT331_ALERT_2_C Small Aver Phenyl C-C Dist C25      --C30       .       1.36 Ang.
PLAT331_ALERT_2_C Small Aver Phenyl C-C Dist C173     --C178      .       1.36 Ang.
PLAT340_ALERT_3_C Low Bond Precision on  C-C Bonds ...............    0.00495 Ang.
PLAT906_ALERT_3_C Large K Value in the Analysis of Variance ......      3.400 Check
PLAT911_ALERT_3_C Missing FCF Refl Between Thmin & STh/L=    0.600         70 Report
```

Alert level G

```
FORMU01_ALERT_1_G  There is a discrepancy between the atom counts in the
            _chemical_formula_sum and _chemical_formula_moiety. This is
            usually due to the moiety formula being in the wrong format.
            Atom count from _chemical_formula_sum:   C169 H140 N4 O12
            Atom count from _chemical_formula_moiety:C170.92 H140 N4 O12.96
PLAT002_ALERT_2_G Number of Distance or Angle Restraints on AtSite          8 Note
PLAT042_ALERT_1_G Calc. and Reported MoietyFormula Strings  Differ     Please Check
PLAT154_ALERT_1_G The s.u.'s on the Cell Angles are Equal ..(Note)      0.002 Degree
PLAT172_ALERT_4_G The CIF-Embedded .res File Contains DFIX Records          3 Report
PLAT606_ALERT_4_G Solvent Accessible VOID(S) in Structure ........          ! Info
PLAT720_ALERT_4_G Number of Unusual/Non-Standard Labels .........           2 Note
```

```
PLAT790_ALERT_4_G Centre of Gravity not Within Unit Cell: Resd.  #          5 Note
              C6 H6
PLAT790_ALERT_4_G Centre of Gravity not Within Unit Cell: Resd.  #          6 Note
              C6 H6
PLAT790_ALERT_4_G Centre of Gravity not Within Unit Cell: Resd.  #          9 Note
              C6 H6
PLAT790_ALERT_4_G Centre of Gravity not Within Unit Cell: Resd.  #         13 Note
              C6 H6
PLAT860_ALERT_3_G Number of Least-Squares Restraints .............          8 Note
PLAT868_ALERT_4_G ALERTS Due to the Use of _smtbx_masks Suppressed          ! Info
PLAT912_ALERT_4_G Missing # of FCF Reflections Above STh/L=  0.600         80 Note
PLAT913_ALERT_3_G Missing # of Very Strong Reflections in FCF ....          1 Note
PLAT933_ALERT_2_G Number of HKL-OMIT Records in Embedded .res File          4 Note
PLAT941_ALERT_3_G Average HKL Measurement Multiplicity ...........        3.3 Low
PLAT978_ALERT_2_G Number C-C Bonds with Positive Residual Density.         10 Info
PLAT992_ALERT_5_G Repd & Actual _reflns_number_gt Values Differ by          4 Check
```

```
   0 ALERT level A = Most likely a serious problem - resolve or explain
   0 ALERT level B = A potentially serious problem, consider carefully
  31 ALERT level C = Check. Ensure it is not caused by an omission or oversight
  19 ALERT level G = General information/check it is not something unexpected

   3 ALERT type 1 CIF construction/syntax error, inconsistent or missing data
  20 ALERT type 2 Indicator that the structure model may be wrong or deficient
   6 ALERT type 3 Indicator that the structure quality may be low
  20 ALERT type 4 Improvement, methodology, query or suggestion
   1 ALERT type 5 Informative message, check
```

It is advisable to attempt to resolve as many as possible of the alerts in all categories. Often the minor alerts point to easily fixed oversights, errors and omissions in your CIF or refinement strategy, so attention to these fine details can be worthwhile. In order to resolve some of the more serious problems it may be necessary to carry out additional measurements or structure refinements. However, the purpose of your study may justify the reported deviations and the more serious of these should normally be commented upon in the discussion or experimental section of a paper or in the "special_details" fields of the CIF. checkCIF was carefully designed to identify outliers and unusual parameters, but every test has its limitations and alerts that are not important in a particular case may appear. Conversely, the absence of alerts does not guarantee there are no aspects of the results needing attention. It is up to the individual to critically assess their own results and, if necessary, seek expert advice.

Publication of your CIF in IUCr journals

A basic structural check has been run on your CIF. These basic checks will be run on all CIFs submitted for publication in IUCr journals (*Acta Crystallographica*, *Journal of Applied Crystallography*, *Journal of Synchrotron Radiation*); however, if you intend to submit to *Acta Crystallographica Section C* or *E* or *IUCrData*, you should make sure that full publication checks are run on the final version of your CIF prior to submission.

Publication of your CIF in other journals

Please refer to the *Notes for Authors* of the relevant journal for any special instructions relating to CIF submission.

PLATON version of 06/07/2023; check.def file version of 30/06/2023

Datablock 1 - ellipsoid plot

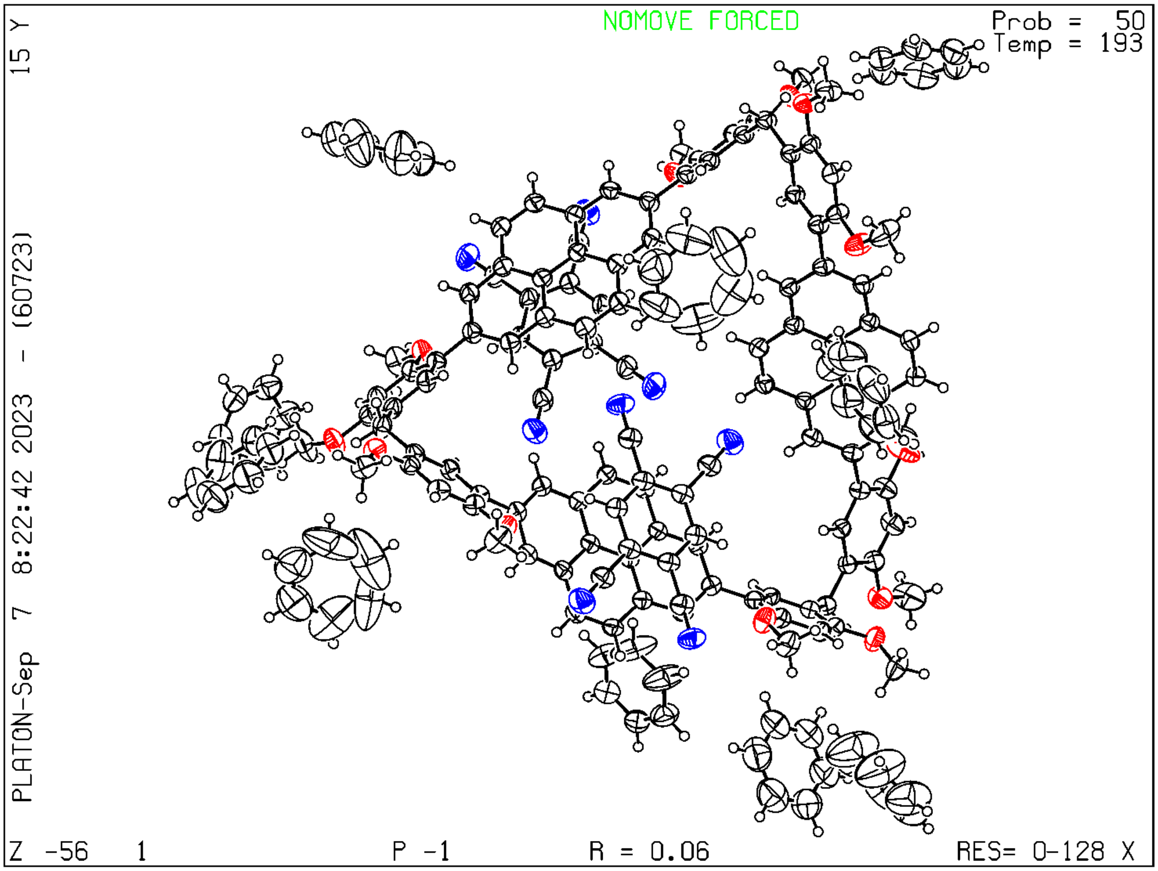

Supplement: Supplementary file 5 — Source Data [file 41467_2024_46788_MOESM5_ESM.zip › Single-crystal structures/MCC-Benzene-checkcif.pdf]
