# Supplementary material for: Structurally diverse macrocycle co-crystals for solid-state luminescence modulation
Source: Nat Commun. 2024 Mar 21;15:2535. doi: 10.1038/s41467-024-46788-6 (PMC10957888; doi:10.1038/s41467-024-46788-6)

checkCIF/PLATON report

Structure factors have been supplied for datablock(s) 2

THIS REPORT IS FOR GUIDANCE ONLY. IF USED AS PART OF A REVIEW PROCEDURE FOR PUBLICATION, IT SHOULD NOT REPLACE THE EXPERTISE OF AN EXPERIENCED CRYSTALLOGRAPHIC REFEREE.

No syntax errors found. CIF dictionary Interpreting this report

Datablock: 2

Bond precision: C-C = 0.0070 A Wavelength=0.71073

Cell:	a=13.9524(8)	b=17.1461(9)	c=22.2786(11)
	alpha=102.009(3)	beta=92.482(3)	gamma=108.301(3)
Temperature:	193 K		

	Calculated	Reported
Volume	4915.4(5)	4915.4(5)
Space group	P -1	P -1
Hall group	-P 1	-P 1
Moiety formula	C99 H77 O12, 0.5(C10 H2 N4), 1.5(C2 H4 Cl2) [+ solvent]	1.5(C2 H4 Cl2), C99 H74 O12, C5 H N2, 0.36[1C3]
Sum formula	C107 H84 Cl3 N2 O12 [+ solvent]	C107 H84 Cl3 N2 O12
Mr	1696.12	1696.11
Dx,g cm-3	1.146	1.146
Z	2	2
Mu (mm-1)	0.152	0.152
F000	1774.0	1774.0
F000’	1775.66	
h,k,lmax	16,20,26	16,20,26
Nref	18244	18034
Tmin,Tmax	0.964,0.985	0.611,0.751
Tmin’	0.955	

Correction method= # Reported T Limits: Tmin=0.611 Tmax=0.751
AbsCorr = NONE

Data completeness= 0.988 Theta(max)= 25.466

R(reflections)= 0.0883(9529) wR2(reflections)= 0.2835(18034)

S = 1.049 Npar= 1128

The following ALERTS were generated. Each ALERT has the format
test-name_ALERT_alert-type_alert-level.
Click on the hyperlinks for more details of the test.

Alert level C

```
PLAT084_ALERT_3_C High wR2 Value (i.e. > 0.25) ...................       0.28 Report
PLAT220_ALERT_2_C NonSolvent   Resd 1  C   Ueq(max)/Ueq(min) Range        5.5 Ratio
PLAT222_ALERT_3_C NonSolvent Resd 1  H   Uiso(max)/Uiso(min) Range        4.9 Ratio
PLAT242_ALERT_2_C Low    'MainMol' Ueq as Compared to Neighbors of        O4 Check
PLAT242_ALERT_2_C Low    'MainMol' Ueq as Compared to Neighbors of        O8 Check
PLAT244_ALERT_4_C Low    'Solvent' Ueq as Compared to Neighbors of      C108 Check
PLAT250_ALERT_2_C Large U3/U1 Ratio for Average U(i,j) Tensor ....        2.1 Note
PLAT260_ALERT_2_C Large Average Ueq of Residue Including       Cl1      0.289 Check
PLAT260_ALERT_2_C Large Average Ueq of Residue Including       Cl3      0.113 Check
PLAT336_ALERT_2_C Long Bond Distance for ..... C108     -Cl1           1.889 Ang.
PLAT340_ALERT_3_C Low Bond Precision on  C-C Bonds ...............    0.00699 Ang.
PLAT911_ALERT_3_C Missing FCF Refl Between Thmin & STh/L=    0.600         49 Report
PLAT918_ALERT_3_C Reflection(s) with I(obs) much Smaller I(calc) .          1 Check
```

Alert level G

```
FORMU01_ALERT_1_G  There is a discrepancy between the atom counts in the
            _chemical_formula_sum and _chemical_formula_moiety. This is
            usually due to the moiety formula being in the wrong format.
            Atom count from _chemical_formula_sum:   C107 H84 Cl3 N2 O12
            Atom count from _chemical_formula_moiety:C108.08 H81 Cl3 N2 O12
PLAT003_ALERT_2_G Number of Uiso or Uij Restrained non-H Atoms ...          1 Report
PLAT042_ALERT_1_G Calc. and Reported MoietyFormula Strings  Differ     Please Check
PLAT072_ALERT_2_G SHELXL First  Parameter in WGHT  Unusually Large       0.13 Report
PLAT154_ALERT_1_G The s.u.'s on the Cell Angles are Equal ..(Note)      0.003 Degree
PLAT186_ALERT_4_G The CIF-Embedded .res File Contains ISOR Records          1 Report
PLAT606_ALERT_4_G Solvent Accessible VOID(S) in Structure ........          ! Info
PLAT790_ALERT_4_G Centre of Gravity not Within Unit Cell: Resd.  #          3 Note
              C2 H4 Cl2
PLAT790_ALERT_4_G Centre of Gravity not Within Unit Cell: Resd.  #          4 Note
              C2 H4 Cl2
PLAT860_ALERT_3_G Number of Least-Squares Restraints .............          6 Note
PLAT868_ALERT_4_G ALERTS Due to the Use of _smtbx_masks Suppressed          ! Info
PLAT912_ALERT_4_G Missing # of FCF Reflections Above STh/L=  0.600        160 Note
PLAT933_ALERT_2_G Number of HKL-OMIT Records in Embedded .res File          2 Note
PLAT941_ALERT_3_G Average HKL Measurement Multiplicity ...........        3.2 Low
PLAT978_ALERT_2_G Number C-C Bonds with Positive Residual Density.          0 Info
```

---

0 **ALERT level A** = Most likely a serious problem - resolve or explain
0 **ALERT level B** = A potentially serious problem, consider carefully
13 **ALERT level C** = Check. Ensure it is not caused by an omission or oversight
15 **ALERT level G** = General information/check it is not something unexpected

3 ALERT type 1 CIF construction/syntax error, inconsistent or missing data

```
11 ALERT type 2 Indicator that the structure model may be wrong or deficient
 7 ALERT type 3 Indicator that the structure quality may be low
 7 ALERT type 4 Improvement, methodology, query or suggestion
 0 ALERT type 5 Informative message, check
```

It is advisable to attempt to resolve as many as possible of the alerts in all categories. Often the minor alerts point to easily fixed oversights, errors and omissions in your CIF or refinement strategy, so attention to these fine details can be worthwhile. In order to resolve some of the more serious problems it may be necessary to carry out additional measurements or structure refinements. However, the purpose of your study may justify the reported deviations and the more serious of these should normally be commented upon in the discussion or experimental section of a paper or in the "special_details" fields of the CIF. checkCIF was carefully designed to identify outliers and unusual parameters, but every test has its limitations and alerts that are not important in a particular case may appear. Conversely, the absence of alerts does not guarantee there are no aspects of the results needing attention. It is up to the individual to critically assess their own results and, if necessary, seek expert advice.

Publication of your CIF in IUCr journals

A basic structural check has been run on your CIF. These basic checks will be run on all CIFs submitted for publication in IUCr journals (*Acta Crystallographica*, *Journal of Applied Crystallography*, *Journal of Synchrotron Radiation*); however, if you intend to submit to *Acta Crystallographica Section C* or *E* or *IUCrData*, you should make sure that full publication checks are run on the final version of your CIF prior to submission.

Publication of your CIF in other journals

Please refer to the *Notes for Authors* of the relevant journal for any special instructions relating to CIF submission.

PLATON version of 06/07/2023; check.def file version of 30/06/2023

Datablock 2 - ellipsoid plot

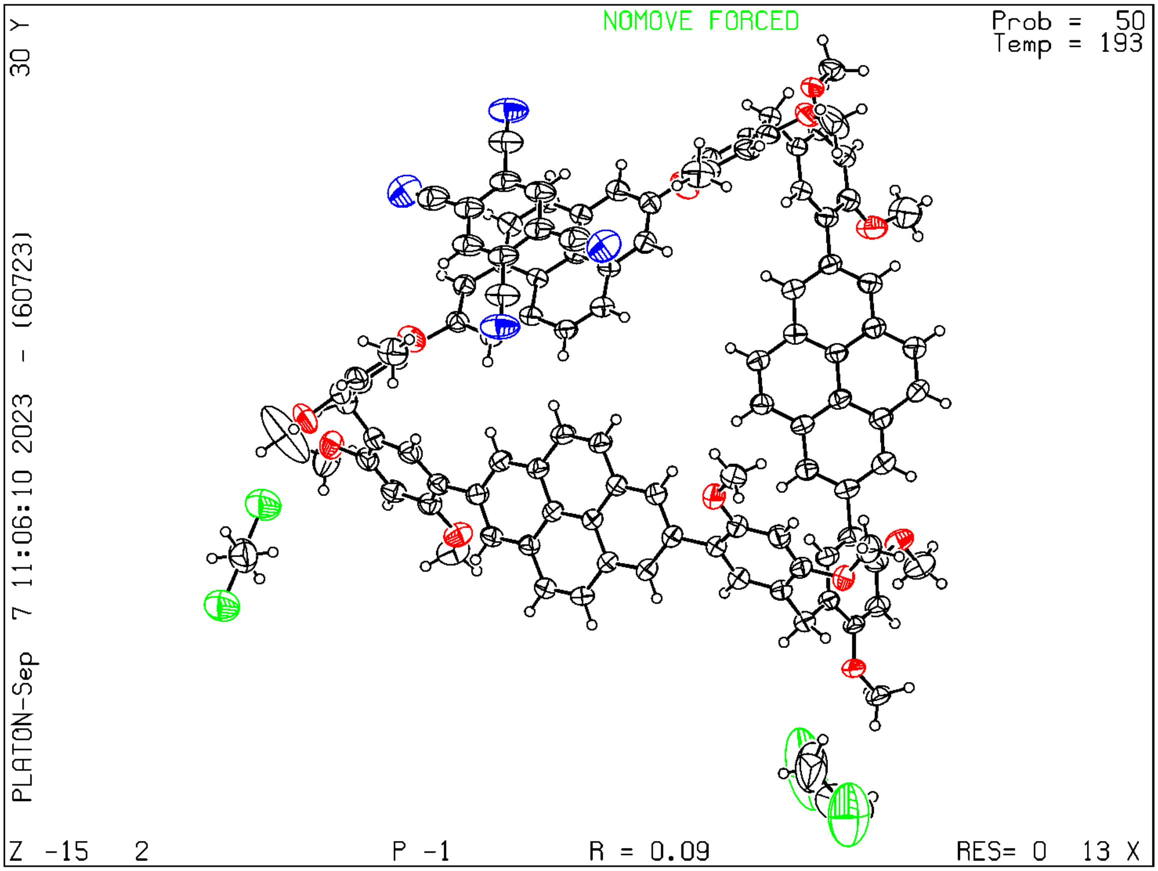

Supplement: Supplementary file 5 — Source Data [file 41467_2024_46788_MOESM5_ESM.zip › Single-crystal structures/MCC-ClCH2CH2Cl-checkcif.pdf]
