# Supplementary material for: Structurally diverse macrocycle co-crystals for solid-state luminescence modulation
Source: Nat Commun. 2024 Mar 21;15:2535. doi: 10.1038/s41467-024-46788-6 (PMC10957888; doi:10.1038/s41467-024-46788-6)

checkCIF/PLATON report

Structure factors have been supplied for datablock(s) lll-1

THIS REPORT IS FOR GUIDANCE ONLY. IF USED AS PART OF A REVIEW PROCEDURE FOR PUBLICATION, IT SHOULD NOT REPLACE THE EXPERTISE OF AN EXPERIENCED CRYSTALLOGRAPHIC REFEREE.

No syntax errors found.   CIF dictionary   Interpreting this report

Datablock: lll-1

Bond precision:	C-C = 0.0107 A		Wavelength=0.71073
Cell:	a=15.6338(10)	b=18.6013(11)	c=23.9689(16)
	alpha=79.798(3)	beta=72.410(4)	gamma=69.806(3)
Temperature:	193 K		

	Calculated	Reported
Volume	6215.3(7)	6215.3(7)
Space group	P -1	P -1
Hall group	-P 1	-P 1
Moiety formula	C99 H78 O12, 1.5(C10 H2 N4), 5(C8 H10)	C99 H78 O12, 3(C5 H N2), 5(C8 H10)
Sum formula	C154 H131 N6 O12	C154 H131 N6 O12
Mr	2257.65	2257.64
Dx,g cm-3	1.206	1.206
Z	2	2
Mu (mm-1)	0.076	0.076
F000	2386.0	2386.0
F000'	2387.01	
h,k,lmax	18,22,29	18,22,28
Nref	23120	22715
Tmin,Tmax	0.982,0.992	0.650,0.751
Tmin'	0.977	

Correction method= # Reported T Limits: Tmin=0.650 Tmax=0.751
AbsCorr = NONE

Data completeness= 0.982   Theta(max)= 25.499

R(reflections)= 0.1201(13746)   wR2(reflections)= 0.3963(22715)

S = 1.031   Npar= 1571

The following ALERTS were generated. Each ALERT has the format
test-name_ALERT_alert-type_alert-level.
Click on the hyperlinks for more details of the test.

Alert level B

```
PLAT084_ALERT_3_B High wR2 Value (i.e. > 0.25) ...................       0.40 Report
```

Author Response: We made several attempts to obtain better quality data set for this crystal. However, due to solvent disorder in phenyl ring and the bad quality of crystals the weighted R factor value could not be lowered. Anyway, there is no doubt about the structure.

```
PLAT340_ALERT_3_B Low Bond Precision on  C-C Bonds ...............    0.01073 Ang.
```

Author Response: This alert is generated because there is a large amount of disorder in the structure.

Alert level C

```
DIFMX02_ALERT_1_C  The maximum difference density is > 0.1*ZMAX*0.75
            The relevant atom site should be identified.
PLAT042_ALERT_1_C Calc. and Reported MoietyFormula Strings  Differ     Please Check
PLAT082_ALERT_2_C High R1 Value .................................       0.12 Report
PLAT097_ALERT_2_C Large Reported Max.  (Positive) Residual Density       0.62 eA-3
PLAT234_ALERT_4_C Large Hirshfeld Difference C21      --C22      .       0.20 Ang.
PLAT234_ALERT_4_C Large Hirshfeld Difference C6       --C7       .       0.16 Ang.
PLAT234_ALERT_4_C Large Hirshfeld Difference C15      --C16      .       0.17 Ang.
PLAT234_ALERT_4_C Large Hirshfeld Difference C109     --C113     .       0.19 Ang.
PLAT243_ALERT_4_C High   'Solvent' Ueq as Compared to Neighbors of        C21 Check
PLAT243_ALERT_4_C High   'Solvent' Ueq as Compared to Neighbors of        C22 Check
PLAT244_ALERT_4_C Low    'Solvent' Ueq as Compared to Neighbors of         C1 Check
PLAT244_ALERT_4_C Low    'Solvent' Ueq as Compared to Neighbors of        C19 Check
PLAT244_ALERT_4_C Low    'Solvent' Ueq as Compared to Neighbors of        C23 Check
PLAT244_ALERT_4_C Low    'Solvent' Ueq as Compared to Neighbors of        C11 Check
PLAT244_ALERT_4_C Low    'Solvent' Ueq as Compared to Neighbors of        C16 Check
PLAT244_ALERT_4_C Low    'Solvent' Ueq as Compared to Neighbors of       C108 Check
PLAT244_ALERT_4_C Low    'Solvent' Ueq as Compared to Neighbors of       C110 Check
PLAT244_ALERT_4_C Low    'Solvent' Ueq as Compared to Neighbors of       C155 Check
PLAT244_ALERT_4_C Low    'Solvent' Ueq as Compared to Neighbors of       C156 Check
PLAT260_ALERT_2_C Large Average Ueq of Residue Including          C1      0.116 Check
PLAT601_ALERT_2_C Unit Cell Contains Solvent Accessible VOIDS of .         34 Ang**3
PLAT906_ALERT_3_C Large K Value in the Analysis of Variance ......     18.265 Check
PLAT906_ALERT_3_C Large K Value in the Analysis of Variance ......      3.655 Check
PLAT906_ALERT_3_C Large K Value in the Analysis of Variance ......      2.035 Check
PLAT911_ALERT_3_C Missing FCF Refl Between Thmin & STh/L=    0.600        241 Report
```

Alert level G

```
PLAT002_ALERT_2_G Number of Distance or Angle Restraints on AtSite         18 Note
PLAT072_ALERT_2_G SHELXL First  Parameter in WGHT  Unusually Large       0.18 Report
```

```
PLAT083_ALERT_2_G SHELXL Second Parameter in WGHT  Unusually Large      30.21 Why ?
PLAT172_ALERT_4_G The CIF-Embedded .res File Contains DFIX Records          4 Report
PLAT380_ALERT_4_G Incorrectly? Oriented X(sp2)-Methyl Moiety .....        C2 Check
PLAT380_ALERT_4_G Incorrectly? Oriented X(sp2)-Methyl Moiety .....       C10 Check
PLAT790_ALERT_4_G Centre of Gravity not Within Unit Cell: Resd.  #          4 Note
              C8 H10
PLAT790_ALERT_4_G Centre of Gravity not Within Unit Cell: Resd.  #          5 Note
              C8 H10
PLAT790_ALERT_4_G Centre of Gravity not Within Unit Cell: Resd.  #          6 Note
              C8 H10
PLAT790_ALERT_4_G Centre of Gravity not Within Unit Cell: Resd.  #          8 Note
              C8 H10
PLAT802_ALERT_4_G CIF Input Record(s) with more than 80 Characters          1 Info
PLAT860_ALERT_3_G Number of Least-Squares Restraints ............          18 Note
PLAT912_ALERT_4_G Missing # of FCF Reflections Above STh/L=  0.600        161 Note
PLAT930_ALERT_2_G FCF-based   Twin Law ( 0 1 0)         Est.d BASF       0.33 Check
PLAT931_ALERT_5_G CIFcalcFCF  Twin Law ( 0 1 0)         Est.d BASF       0.33 Check
PLAT933_ALERT_2_G Number of HKL-OMIT Records in Embedded .res File         86 Note
PLAT941_ALERT_3_G Average HKL Measurement Multiplicity ..........         3.2 Low
PLAT978_ALERT_2_G Number C-C Bonds with Positive Residual Density.          0 Info
PLAT992_ALERT_5_G Repd & Actual _reflns_number_gt Values Differ by          3 Check
```

---

0 **ALERT level A** = Most likely a serious problem - resolve or explain
2 **ALERT level B** = A potentially serious problem, consider carefully
25 **ALERT level C** = Check. Ensure it is not caused by an omission or oversight
19 **ALERT level G** = General information/check it is not something unexpected

2 ALERT type 1 CIF construction/syntax error, inconsistent or missing data
10 ALERT type 2 Indicator that the structure model may be wrong or deficient
8 ALERT type 3 Indicator that the structure quality may be low
24 ALERT type 4 Improvement, methodology, query or suggestion
2 ALERT type 5 Informative message, check

It is advisable to attempt to resolve as many as possible of the alerts in all categories. Often the minor alerts point to easily fixed oversights, errors and omissions in your CIF or refinement strategy, so attention to these fine details can be worthwhile. In order to resolve some of the more serious problems it may be necessary to carry out additional measurements or structure refinements. However, the purpose of your study may justify the reported deviations and the more serious of these should normally be commented upon in the discussion or experimental section of a paper or in the "special_details" fields of the CIF. checkCIF was carefully designed to identify outliers and unusual parameters, but every test has its limitations and alerts that are not important in a particular case may appear. Conversely, the absence of alerts does not guarantee there are no aspects of the results needing attention. It is up to the individual to critically assess their own results and, if necessary, seek expert advice.

Publication of your CIF in IUCr journals

A basic structural check has been run on your CIF. These basic checks will be run on all CIFs submitted for publication in IUCr journals (*Acta Crystallographica*, *Journal of Applied Crystallography*, *Journal of Synchrotron Radiation*); however, if you intend to submit to *Acta Crystallographica Section C* or *E* or *IUCrData*, you should make sure that full publication checks are run on the final version of your CIF prior to submission.

Publication of your CIF in other journals

Please refer to the *Notes for Authors* of the relevant journal for any special instructions relating to CIF submission.

PLATON version of 06/07/2023; check.def file version of 30/06/2023

Datablock lll-1 - ellipsoid plot

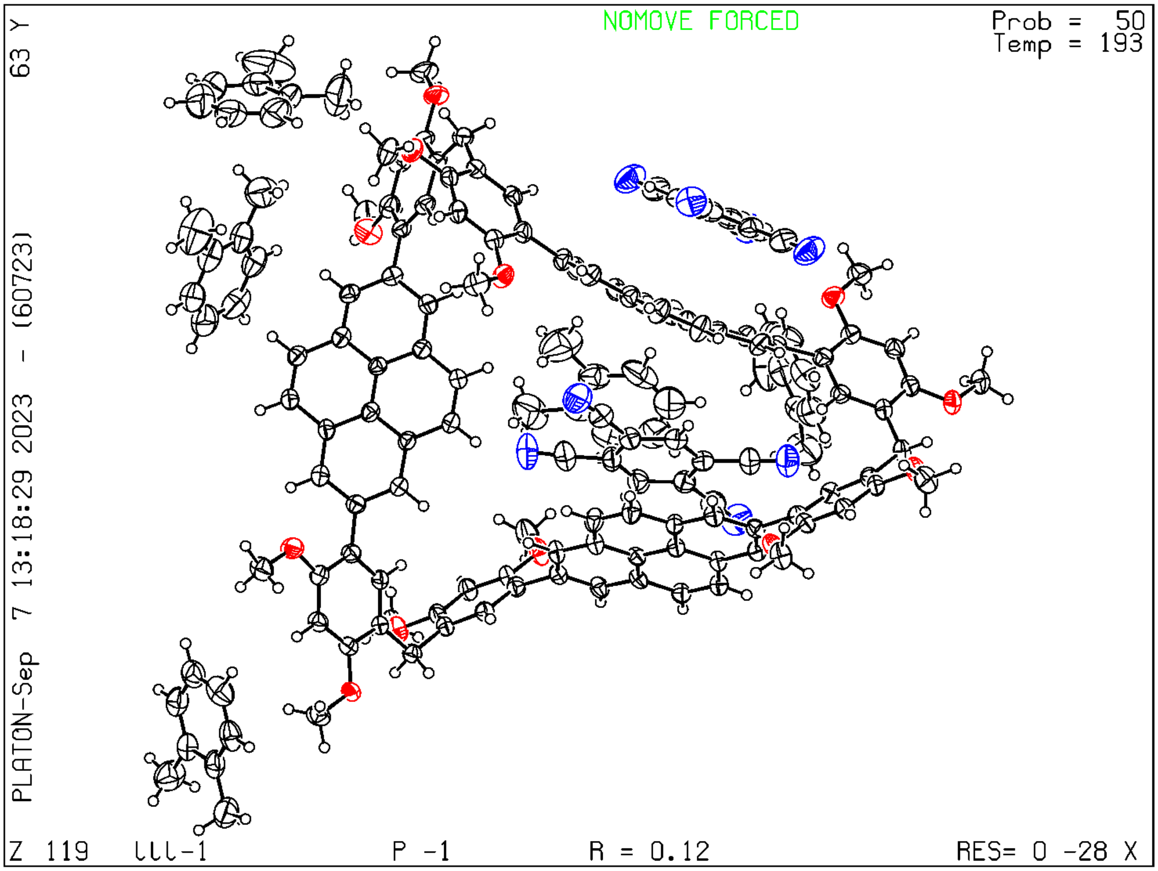

Supplement: Supplementary file 5 — Source Data [file 41467_2024_46788_MOESM5_ESM.zip › Single-crystal structures/MCC-o-Xylene-checkcif.pdf]
