# Supplementary material for: Structurally diverse macrocycle co-crystals for solid-state luminescence modulation
Source: Nat Commun. 2024 Mar 21;15:2535. doi: 10.1038/s41467-024-46788-6 (PMC10957888; doi:10.1038/s41467-024-46788-6)

checkCIF/PLATON report

Structure factors have been supplied for datablock(s) 1

THIS REPORT IS FOR GUIDANCE ONLY. IF USED AS PART OF A REVIEW PROCEDURE FOR PUBLICATION, IT SHOULD NOT REPLACE THE EXPERTISE OF AN EXPERIENCED CRYSTALLOGRAPHIC REFEREE.

No syntax errors found. CIF dictionary Interpreting this report

Datablock: 1

Bond precision:	C-C = 0.0030 A	Wavelength=0.71073	
Cell:	a=38.5418(18)	b=38.5418(18)	c=11.7183(6)
	alpha=90	beta=90	gamma=120
Temperature:	193 K		

	Calculated	Reported
Volume	15075.1(16)	15075.1(16)
Space group	R -3	R -3
Hall group	-R 3	-R 3
Moiety formula	C99 H78 O12, 3(C3 H6 N O) [+ solvent]	C99 H78 O12, 3(C3 H6 N O)
Sum formula	C108 H96 N3 O15 [+ solvent]	C108 H99 N3 O15
Mr	1675.88	1678.90
Dx,g cm-3	1.108	1.110
Z	6	6
Mu (mm-1)	0.074	0.074
F000	5310.0	5328.0
F000'	5312.50	
h,k,lmax	50,50,15	50,50,15
Nref	7794	7763
Tmin,Tmax	0.982,0.993	0.699,0.746
Tmin'	0.978	

Correction method= # Reported T Limits: Tmin=0.699 Tmax=0.746
AbsCorr = NONE

Data completeness= 0.996 Theta(max)= 27.604

R(reflections)= 0.0608(5822) wR2(reflections)= 0.1779(7763)

S = 1.027 Npar= 385

The following ALERTS were generated. Each ALERT has the format
test-name_ALERT_alert-type_alert-level.
Click on the hyperlinks for more details of the test.

Alert level C

```
DIFMX02_ALERT_1_C  The maximum difference density is > 0.1*ZMAX*0.75
            The relevant atom site should be identified.
PLAT094_ALERT_2_C Ratio of Maximum / Minimum Residual Density ....       2.30 Report
PLAT097_ALERT_2_C Large Reported Max.  (Positive) Residual Density       0.65 eA-3
PLAT911_ALERT_3_C Missing FCF Refl Between Thmin & STh/L=    0.600         14 Report
PLAT913_ALERT_3_C Missing # of Very Strong Reflections in FCF ....          5 Note
```

Alert level G

```
FORMU01_ALERT_1_G  There is a discrepancy between the atom counts in the
            _chemical_formula_sum and _chemical_formula_moiety. This is
            usually due to the moiety formula being in the wrong format.
            Atom count from _chemical_formula_sum:   C108 H99 N3 O15
            Atom count from _chemical_formula_moiety:C108 H96 N3 O15
FORMU01_ALERT_2_G  There is a discrepancy between the atom counts in the
            _chemical_formula_sum and the formula from the _atom_site* data.
            Atom count from _chemical_formula_sum:C108 H99 N3 O15
            Atom count from the _atom_site data:  C108 H96 N3 O15
CELLZ01_ALERT_1_G Difference between formula and atom_site contents detected.
CELLZ01_ALERT_1_G WARNING: H atoms missing from atom site list. Is this intentional?
           From the CIF: _cell_formula_units_Z    6
           From the CIF: _chemical_formula_sum  C108 H99 N3 O15
           TEST: Compare cell contents of formula and atom_site data

           atom    Z*formula  cif sites diff
           C        648.00    648.00    0.00
           H        594.00    576.00   18.00
           N         18.00     18.00    0.00
           O         90.00     90.00    0.00
PLAT041_ALERT_1_G Calc. and Reported SumFormula    Strings  Differ     Please Check
PLAT083_ALERT_2_G SHELXL Second Parameter in WGHT  Unusually Large      27.10 Why ?
PLAT344_ALERT_2_G Unusual sp?       Angle Range in Solvent/Ion for         C3 Check
PLAT606_ALERT_4_G Solvent Accessible VOID(S) in Structure ........          ! Info
PLAT720_ALERT_4_G Number of Unusual/Non-Standard Labels ..........          7 Note
PLAT868_ALERT_4_G ALERTS Due to the Use of _smtbx_masks Suppressed          ! Info
PLAT910_ALERT_3_G Missing # of FCF Reflection(s) Below Theta(Min).          2 Note
PLAT912_ALERT_4_G Missing # of FCF Reflections Above STh/L=  0.600         17 Note
PLAT933_ALERT_2_G Number of HKL-OMIT Records in Embedded .res File          1 Note
PLAT978_ALERT_2_G Number C-C Bonds with Positive Residual Density.         16 Info
```

---

0 **ALERT level A** = Most likely a serious problem - resolve or explain
0 **ALERT level B** = A potentially serious problem, consider carefully
5 **ALERT level C** = Check. Ensure it is not caused by an omission or oversight
14 **ALERT level G** = General information/check it is not something unexpected

5 ALERT type 1 CIF construction/syntax error, inconsistent or missing data

```
 7 ALERT type 2 Indicator that the structure model may be wrong or deficient
 3 ALERT type 3 Indicator that the structure quality may be low
 4 ALERT type 4 Improvement, methodology, query or suggestion
 0 ALERT type 5 Informative message, check
```

It is advisable to attempt to resolve as many as possible of the alerts in all categories. Often the minor alerts point to easily fixed oversights, errors and omissions in your CIF or refinement strategy, so attention to these fine details can be worthwhile. In order to resolve some of the more serious problems it may be necessary to carry out additional measurements or structure refinements. However, the purpose of your study may justify the reported deviations and the more serious of these should normally be commented upon in the discussion or experimental section of a paper or in the "special_details" fields of the CIF. checkCIF was carefully designed to identify outliers and unusual parameters, but every test has its limitations and alerts that are not important in a particular case may appear. Conversely, the absence of alerts does not guarantee there are no aspects of the results needing attention. It is up to the individual to critically assess their own results and, if necessary, seek expert advice.

Publication of your CIF in IUCr journals

A basic structural check has been run on your CIF. These basic checks will be run on all CIFs submitted for publication in IUCr journals (*Acta Crystallographica*, *Journal of Applied Crystallography*, *Journal of Synchrotron Radiation*); however, if you intend to submit to *Acta Crystallographica Section C* or *E* or *IUCrData*, you should make sure that full publication checks are run on the final version of your CIF prior to submission.

Publication of your CIF in other journals

Please refer to the *Notes for Authors* of the relevant journal for any special instructions relating to CIF submission.

PLATON version of 06/07/2023; check.def file version of 30/06/2023

Datablock 1 - ellipsoid plot

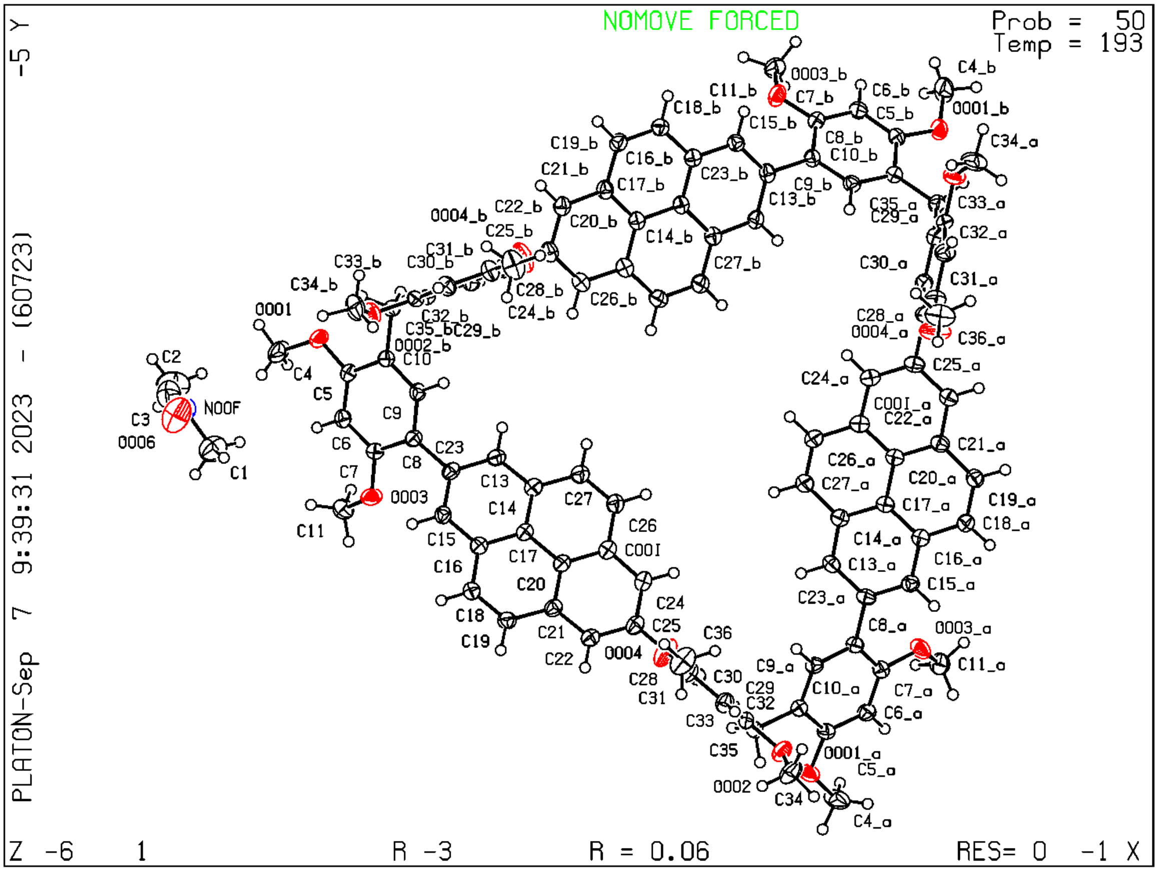

Supplement: Supplementary file 5 — Source Data [file 41467_2024_46788_MOESM5_ESM.zip › Single-crystal structures/Pe[3]-DMF-checkcif.pdf]
