# Supplementary material for: Structurally diverse macrocycle co-crystals for solid-state luminescence modulation
Source: Nat Commun. 2024 Mar 21;15:2535. doi: 10.1038/s41467-024-46788-6 (PMC10957888; doi:10.1038/s41467-024-46788-6)

checkCIF/PLATON report

Structure factors have been supplied for datablock(s) lb-4

THIS REPORT IS FOR GUIDANCE ONLY. IF USED AS PART OF A REVIEW PROCEDURE FOR PUBLICATION, IT SHOULD NOT REPLACE THE EXPERTISE OF AN EXPERIENCED CRYSTALLOGRAPHIC REFEREE.

No syntax errors found. CIF dictionary Interpreting this report

Datablock: lb-4

Bond precision: C-C = 0.0032 A Wavelength=0.71073

Cell:	a=7.5708(9)	b=11.5989(13)	c=11.9686(14)
	alpha=67.249(2)	beta=78.962(2)	gamma=89.497(2)
Temperature:	296 K		

	Calculated	Reported
Volume	948.82(19)	948.82(19)
Space group	P -1	P -1
Hall group	-P 1	-P 1
Moiety formula	C32 H26 O4, C10 H2 N4, C4 H8 O2	C32 H26 O4, C10 H2 N4, C4 H8 O2
Sum formula	C46 H36 N4 O6	C46 H36 N4 O6
Mr	740.79	740.79
Dx,g cm-3	1.296	1.296
Z	1	1
Mu (mm-1)	0.087	0.087
F000	388.0	388.0
F000'	388.18	
h,k,lmax	9,15,15	9,15,15
Nref	4441	4200
Tmin,Tmax	0.982,0.984	0.668,0.746
Tmin'	0.982	

Correction method= # Reported T Limits: Tmin=0.668 Tmax=0.746
AbsCorr = MULTI-SCAN

Data completeness= 0.946 Theta(max)= 27.711

R(reflections)= 0.0563(2720) wR2(reflections)= 0.1757(4200)

S = 1.031 Npar= 255

The following ALERTS were generated. Each ALERT has the format
test-name_ALERT_alert-type_alert-level.
Click on the hyperlinks for more details of the test.

Alert level B

```
PLAT097_ALERT_2_B Large Reported Max.  (Positive) Residual Density       0.86 eA-3
```

Author Response: It is due to disorder in the structure.

Alert level C

```
DIFMX02_ALERT_1_C  The maximum difference density is > 0.1*ZMAX*0.75
            The relevant atom site should be identified.
PLAT094_ALERT_2_C Ratio of Maximum / Minimum Residual Density ....       2.44 Report
PLAT193_ALERT_1_C Cell and Diffraction Temperatures Differ by ....          2 Degree
PLAT911_ALERT_3_C Missing FCF Refl Between Thmin & STh/L=    0.600         23 Report
```

Alert level G

```
PLAT154_ALERT_1_G The s.u.'s on the Cell Angles are Equal ..(Note)      0.002 Degree
PLAT398_ALERT_2_G Deviating  C-O-C   Angle From 120 for O3       .      109.4 Degree
PLAT910_ALERT_3_G Missing # of FCF Reflection(s) Below Theta(Min).          1 Note
PLAT912_ALERT_4_G Missing # of FCF Reflections Above STh/L=  0.600        218 Note
PLAT941_ALERT_3_G Average HKL Measurement Multiplicity ..........         1.4 Low
PLAT978_ALERT_2_G Number C-C Bonds with Positive Residual Density.          3 Info
PLAT992_ALERT_5_G Repd & Actual _reflns_number_gt Values Differ by          4 Check
```

0 **ALERT level A** = Most likely a serious problem - resolve or explain
1 **ALERT level B** = A potentially serious problem, consider carefully
4 **ALERT level C** = Check. Ensure it is not caused by an omission or oversight
7 **ALERT level G** = General information/check it is not something unexpected

3 ALERT type 1 CIF construction/syntax error, inconsistent or missing data
4 ALERT type 2 Indicator that the structure model may be wrong or deficient
3 ALERT type 3 Indicator that the structure quality may be low
1 ALERT type 4 Improvement, methodology, query or suggestion
1 ALERT type 5 Informative message, check

It is advisable to attempt to resolve as many as possible of the alerts in all categories. Often the minor alerts point to easily fixed oversights, errors and omissions in your CIF or refinement strategy, so attention to these fine details can be worthwhile. In order to resolve some of the more serious problems it may be necessary to carry out additional measurements or structure refinements. However, the purpose of your study may justify the reported deviations and the more serious of these should normally be commented upon in the discussion or experimental section of a paper or in the "special_details" fields of the CIF. checkCIF was carefully designed to identify outliers and unusual parameters, but every test has its limitations and alerts that are not important in a particular case may appear. Conversely, the absence of alerts does not guarantee there are no aspects of the results needing attention. It is up to the individual to critically assess their own results and, if necessary, seek expert advice.

Publication of your CIF in IUCr journals

A basic structural check has been run on your CIF. These basic checks will be run on all CIFs submitted for publication in IUCr journals (*Acta Crystallographica*, *Journal of Applied Crystallography*, *Journal of Synchrotron Radiation*); however, if you intend to submit to *Acta Crystallographica Section C* or *E* or *IUCrData*, you should make sure that full publication checks are run on the final version of your CIF prior to submission.

Publication of your CIF in other journals

Please refer to the *Notes for Authors* of the relevant journal for any special instructions relating to CIF submission.

PLATON version of 28/11/2022; check.def file version of 28/11/2022

Datablock lb-4 - ellipsoid plot

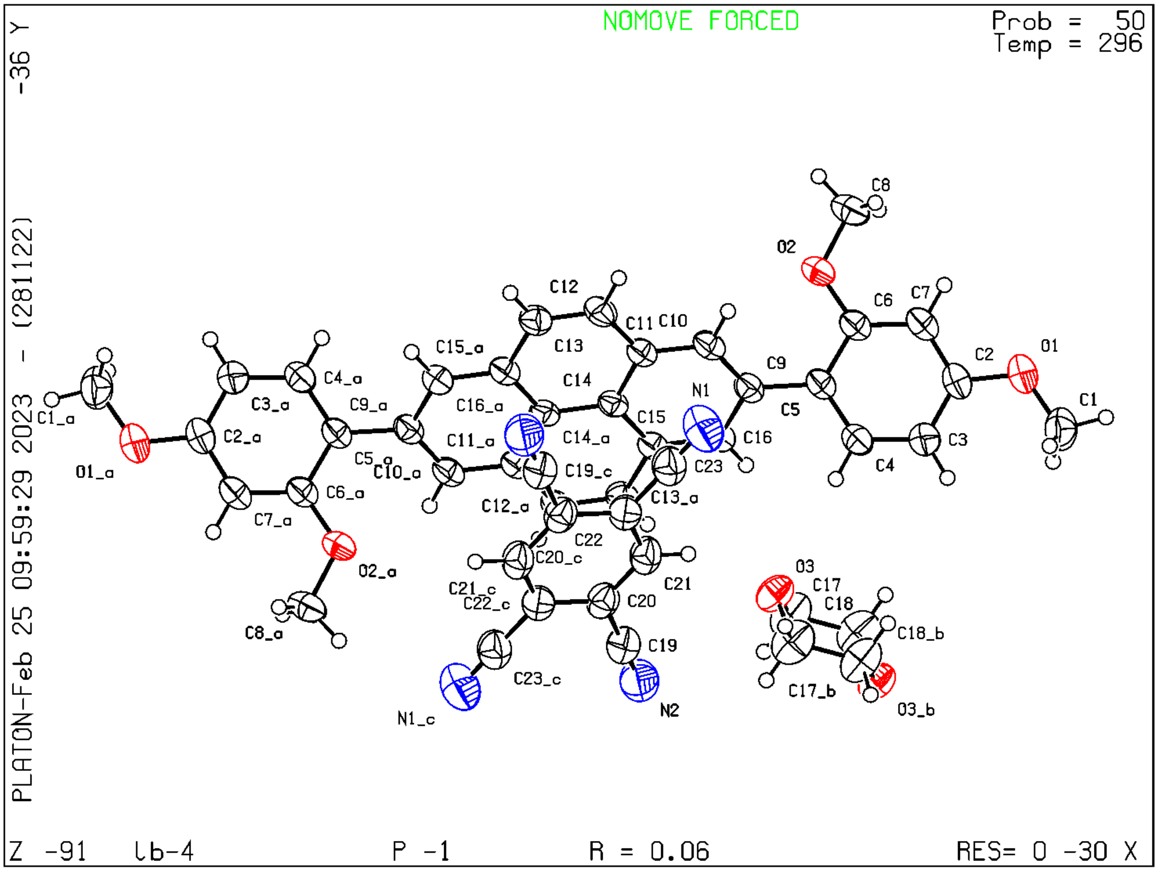

Supplement: Supplementary file 5 — Source Data [file 41467_2024_46788_MOESM5_ESM.zip › Single-crystal structures/Pe-TCNB-checkcif.pdf]
