# Supplementary material for: Structurally diverse macrocycle co-crystals for solid-state luminescence modulation
Source: Nat Commun. 2024 Mar 21;15:2535. doi: 10.1038/s41467-024-46788-6 (PMC10957888; doi:10.1038/s41467-024-46788-6)

checkCIF/PLATON report

Structure factors have been supplied for datablock(s) lll-1

THIS REPORT IS FOR GUIDANCE ONLY. IF USED AS PART OF A REVIEW PROCEDURE FOR PUBLICATION, IT SHOULD NOT REPLACE THE EXPERTISE OF AN EXPERIENCED CRYSTALLOGRAPHIC REFEREE.

No syntax errors found.   CIF dictionary   Interpreting this report

Datablock: lll-1

Bond precision:	C-C = 0.0020 A		Wavelength=0.71073
Cell:	a=8.733(4)	b=19.954(9)	c=7.088(3)
	alpha=90	beta=102.94(3)	gamma=90
Temperature:	296 K		

	Calculated	Reported
Volume	1203.8(9)	1203.8(9)
Space group	P 21/c	P 1 21/c 1
Hall group	-P 2ybc	-P 2ybc
Moiety formula	C32 H26 O4	C32 H26 O4
Sum formula	C32 H26 O4	C32 H26 O4
Mr	474.53	474.53
Dx,g cm-3	1.309	1.309
Z	2	2
Mu (mm-1)	0.085	0.085
F000	500.0	500.0
F000'	500.24	
h,k,lmax	11,26,9	11,26,9
Nref	3028	2991
Tmin,Tmax	0.989,0.991	
Tmin'	0.989	

Correction method= Not given

Data completeness= 0.988   Theta(max)= 28.393

R(reflections)= 0.0491(2287)   wR2(reflections)= 0.1488(2991)

S = 1.037   Npar= 165

The following ALERTS were generated. Each ALERT has the format
test-name_ALERT_alert-type_alert-level.
Click on the hyperlinks for more details of the test.

Alert level C

```
PLAT911_ALERT_3_C Missing FCF Refl Between Thmin & STh/L=    0.600           8 Report
```

Alert level G

```
PLAT912_ALERT_4_G Missing # of FCF Reflections Above STh/L=  0.600          30 Note
PLAT913_ALERT_3_G Missing # of Very Strong Reflections in FCF ....           1 Note
PLAT941_ALERT_3_G Average HKL Measurement Multiplicity ..........          3.8 Low
PLAT978_ALERT_2_G Number C-C Bonds with Positive Residual Density.          16 Info
```

0 **ALERT level A** = Most likely a serious problem - resolve or explain
0 **ALERT level B** = A potentially serious problem, consider carefully
1 **ALERT level C** = Check. Ensure it is not caused by an omission or oversight
4 **ALERT level G** = General information/check it is not something unexpected

0 ALERT type 1 CIF construction/syntax error, inconsistent or missing data
1 ALERT type 2 Indicator that the structure model may be wrong or deficient
3 ALERT type 3 Indicator that the structure quality may be low
1 ALERT type 4 Improvement, methodology, query or suggestion
0 ALERT type 5 Informative message, check

It is advisable to attempt to resolve as many as possible of the alerts in all categories. Often the minor alerts point to easily fixed oversights, errors and omissions in your CIF or refinement strategy, so attention to these fine details can be worthwhile. In order to resolve some of the more serious problems it may be necessary to carry out additional measurements or structure refinements. However, the purpose of your study may justify the reported deviations and the more serious of these should normally be commented upon in the discussion or experimental section of a paper or in the "special_details" fields of the CIF. checkCIF was carefully designed to identify outliers and unusual parameters, but every test has its limitations and alerts that are not important in a particular case may appear. Conversely, the absence of alerts does not guarantee there are no aspects of the results needing attention. It is up to the individual to critically assess their own results and, if necessary, seek expert advice.

Publication of your CIF in IUCr journals

A basic structural check has been run on your CIF. These basic checks will be run on all CIFs submitted for publication in IUCr journals (*Acta Crystallographica*, *Journal of Applied Crystallography*, *Journal of Synchrotron Radiation*); however, if you intend to submit to *Acta Crystallographica Section C* or *E* or *IUCrData*, you should make sure that full publication checks are run on the final version of your CIF prior to submission.

Publication of your CIF in other journals

Please refer to the *Notes for Authors* of the relevant journal for any special instructions relating to CIF submission.

PLATON version of 06/07/2023; check.def file version of 30/06/2023

Datablock lll-1 - ellipsoid plot

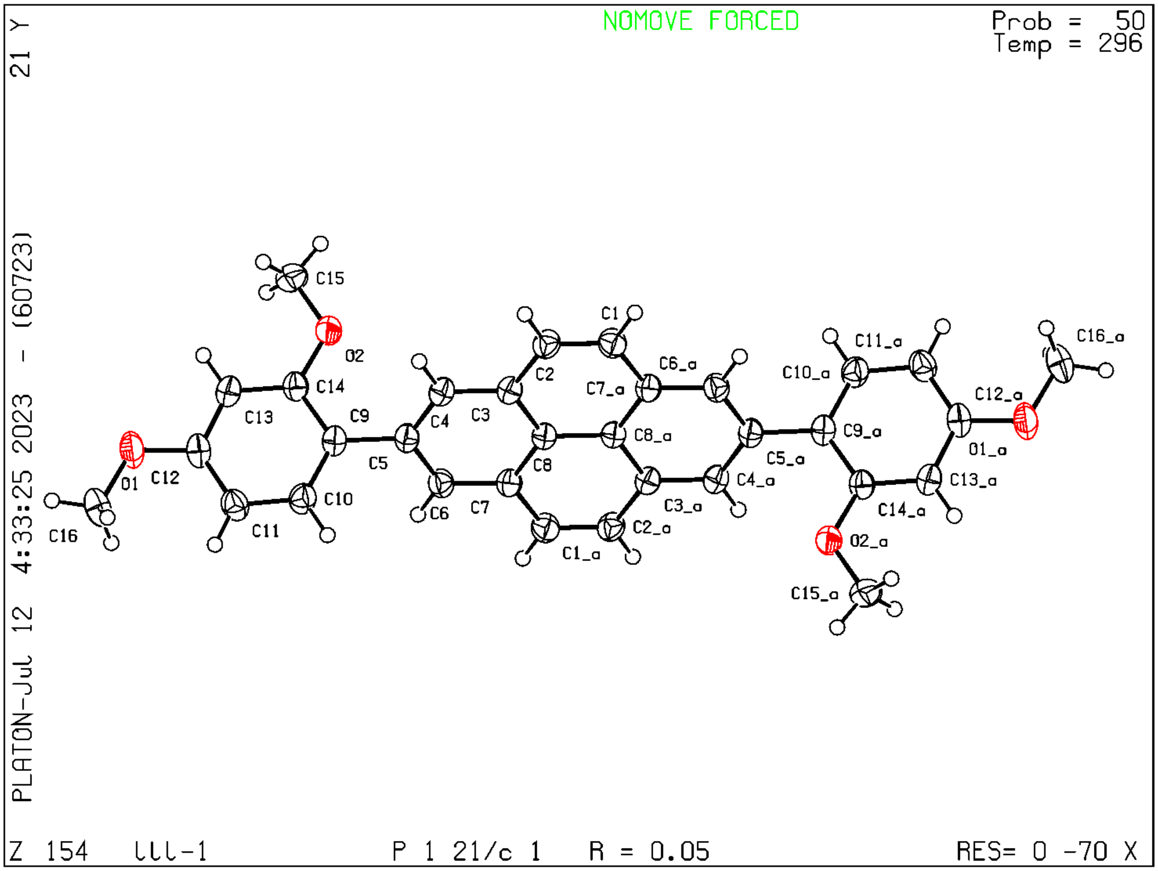

Supplement: Supplementary file 5 — Source Data [file 41467_2024_46788_MOESM5_ESM.zip › Single-crystal structures/Pe-THF-checkcif.pdf]
