# Supplementary material for: Structurally diverse macrocycle co-crystals for solid-state luminescence modulation
Source: Nat Commun. 2024 Mar 21;15:2535. doi: 10.1038/s41467-024-46788-6 (PMC10957888; doi:10.1038/s41467-024-46788-6)

# checkCIF/PLATON report

Structure factors have been supplied for datablock(s) mo_2012074967_0m

THIS REPORT IS FOR GUIDANCE ONLY. IF USED AS PART OF A REVIEW PROCEDURE FOR PUBLICATION, IT SHOULD NOT REPLACE THE EXPERTISE OF AN EXPERIENCED CRYSTALLOGRAPHIC REFEREE.

No syntax errors found. CIF dictionary Interpreting this report

# Datablock: mo_2012074967_0m

---

Bond precision: C-C = 0.0226 A Wavelength=0.71073

| | | | |
|---|---|---|---|
| Cell: | a=16.073(3) | b=17.902(3) | c=23.748(4) |
| | alpha=108.627(5) | beta=92.658(6) | gamma=102.860(6) |
| Temperature: | 120 K | | |

| | Calculated | Reported |
|---|---|---|
| Volume | 6261.6(19) | 6262(2) |
| Space group | P -1 | P -1 |
| Hall group | -P 1 | -P 1 |
| Moiety formula | C99 H78 O12, 0.5(C10 H2 N4), 11(C4 O) | C99 H78 O12, 11(C4 O), C5 H N2 |
| Sum formula | C148 H79 N2 O23 | C148 H79 N2 O23 |
| Mr | 2253.14 | 2253.13 |
| Dx,g cm-3 | 1.195 | 1.195 |
| Z | 2 | 2 |
| Mu (mm-1) | 0.081 | 0.081 |
| F000 | 2330.0 | 2330.0 |
| F000' | 2331.20 | |
| h,k,lmax | 14,15,20 | 13,15,20 |
| Nref | 8966 | 8779 |
| Tmin,Tmax | 0.985,0.991 | 0.570,0.744 |
| Tmin' | 0.984 | |

Correction method= # Reported T Limits: Tmin=0.570 Tmax=0.744
AbsCorr = MULTI-SCAN

Data completeness= 0.979 Theta(max)= 18.242

R(reflections)= 0.1442( 6464) wR2(reflections)= 0.4441( 8779)

S = 1.906 Npar= 1570

The following ALERTS were generated. Each ALERT has the format
**test-name_ALERT_alert-type_alert-level.**
Click on the hyperlinks for more details of the test.

### Alert level A

THETM01_ALERT_3_A The value of sine(theta_max)/wavelength is less than 0.550
Calculated sin(theta_max)/wavelength = 0.4404

**Author Response: As described above, the crystal structure is solvent-rich and disordered, leading to scatter weakly at high angle.**

PLAT088_ALERT_3_A Poor Data / Parameter Ratio .................... 5.59 Note

**Author Response: The reflections to parameters ratio is low due to the number of refined parameters necessary to describe the disorder and the weakly diffracting nature of the crystal at high angle.**

### Alert level B

PLAT084_ALERT_3_B High wR2 Value (i.e. > 0.25) .................. 0.44 Report

**Author Response: The structure contains large voids, which have highly disordered solvent molecules inside. This causes the poor quality of the single crystal of the title compound.**

PLAT097_ALERT_2_B Large Reported Max. (Positive) Residual Density 0.98 eA-3

**Author Response: The crystal structure is a solvent-rich solvate and contains 11 THF molecules per asymmetric unit. These THF molecules are disordered and show in part large anisotropic displacement ellipsoids.**

PLAT260_ALERT_2_B Large Average Ueq of Residue Including O23 0.489 Check

**Author Response: It could be due to disordered solvent molecules.**

PLAT340_ALERT_3_B Low Bond Precision on C-C Bonds .............. 0.02258 Ang.

**Author Response: Disorder present in the structure.**

PLAT910_ALERT_3_B Missing # of FCF Reflection(s) Below Theta(Min). 11 Note

**Author Response: The missing FCF reflections below theta(min) were caused by the high beamstop theta(min) limit set.**

```
PLAT922_ALERT_1_B wR2 in the CIF and FCF Differ by ..............     0.0067 Check
```

**Author Response: The spot quality of this crystal was not ideal. Most of the reflection were streaks. Numerous restraints and constraints were still needed to obtain a model that converged. The data reported here is to establish the connectivity of the complex.**

```
PLAT934_ALERT_3_B Number of (Iobs-Icalc)/Sigma(W) > 10 Outliers ..          5 Check
```

**Author Response: Because of highly disordered guest molecules (THF), some reflections did not match between observation and calculations.**

---

## Alert level C

```
DIFMX02_ALERT_1_C  The maximum difference density is > 0.1*ZMAX*0.75
            The relevant atom site should be identified.
PLAT029_ALERT_3_C _diffrn_measured_fraction_theta_full value Low .      0.979 Why?
PLAT042_ALERT_1_C Calc. and Reported MoietyFormula Strings  Differ     Please Check
PLAT082_ALERT_2_C High R1 Value ................................       0.14 Report
PLAT243_ALERT_4_C High   'Solvent' Ueq as Compared to Neighbors of       C109 Check
PLAT243_ALERT_4_C High   'Solvent' Ueq as Compared to Neighbors of       C124 Check
PLAT243_ALERT_4_C High   'Solvent' Ueq as Compared to Neighbors of       C119 Check
PLAT243_ALERT_4_C High   'Solvent' Ueq as Compared to Neighbors of       C125 Check
PLAT243_ALERT_4_C High   'Solvent' Ueq as Compared to Neighbors of        O17 Check
PLAT243_ALERT_4_C High   'Solvent' Ueq as Compared to Neighbors of        O18 Check
PLAT243_ALERT_4_C High   'Solvent' Ueq as Compared to Neighbors of       C128 Check
PLAT243_ALERT_4_C High   'Solvent' Ueq as Compared to Neighbors of       C133 Check
PLAT243_ALERT_4_C High   'Solvent' Ueq as Compared to Neighbors of        O19 Check
PLAT243_ALERT_4_C High   'Solvent' Ueq as Compared to Neighbors of        O20 Check
PLAT243_ALERT_4_C High   'Solvent' Ueq as Compared to Neighbors of       C136 Check
PLAT243_ALERT_4_C High   'Solvent' Ueq as Compared to Neighbors of        O21 Check
PLAT243_ALERT_4_C High   'Solvent' Ueq as Compared to Neighbors of       C143 Check
PLAT243_ALERT_4_C High   'Solvent' Ueq as Compared to Neighbors of       C139 Check
PLAT243_ALERT_4_C High   'Solvent' Ueq as Compared to Neighbors of       C140 Check
PLAT244_ALERT_4_C Low    'Solvent' Ueq as Compared to Neighbors of        C91 Check
PLAT244_ALERT_4_C Low    'Solvent' Ueq as Compared to Neighbors of        O15 Check
PLAT244_ALERT_4_C Low    'Solvent' Ueq as Compared to Neighbors of       C110 Check
PLAT244_ALERT_4_C Low    'Solvent' Ueq as Compared to Neighbors of       C112 Check
PLAT244_ALERT_4_C Low    'Solvent' Ueq as Compared to Neighbors of       C134 Check
PLAT244_ALERT_4_C Low    'Solvent' Ueq as Compared to Neighbors of       C114 Check
PLAT244_ALERT_4_C Low    'Solvent' Ueq as Compared to Neighbors of       C121 Check
PLAT244_ALERT_4_C Low    'Solvent' Ueq as Compared to Neighbors of       C115 Check
PLAT244_ALERT_4_C Low    'Solvent' Ueq as Compared to Neighbors of       C127 Check
PLAT244_ALERT_4_C Low    'Solvent' Ueq as Compared to Neighbors of       C105 Check
PLAT244_ALERT_4_C Low    'Solvent' Ueq as Compared to Neighbors of       C138 Check
PLAT250_ALERT_2_C Large U3/U1 Ratio for Average U(i,j) Tensor ....        2.2 Note
PLAT260_ALERT_2_C Large Average Ueq of Residue Including       O14      0.114 Check
```

**Author Response: It could be due to disordered solvent molecules.**

```
PLAT260_ALERT_2_C Large Average Ueq of Residue Including       O15       0.152 Check
```

**Author Response: It could be due to disordered solvent molecules.**

```
PLAT260_ALERT_2_C Large Average Ueq of Residue Including       O16       0.125 Check
```

**Author Response: It could be due to disordered solvent molecules.**

```
PLAT260_ALERT_2_C Large Average Ueq of Residue Including       O17       0.204 Check
```

**Author Response: It could be due to disordered solvent molecules.**

```
PLAT260_ALERT_2_C Large Average Ueq of Residue Including       O18       0.203 Check
```

**Author Response: It could be due to disordered solvent molecules.**

```
PLAT260_ALERT_2_C Large Average Ueq of Residue Including       O19       0.227 Check
```

**Author Response: It could be due to disordered solvent molecules.**

```
PLAT260_ALERT_2_C Large Average Ueq of Residue Including       O20       0.195 Check
```

**Author Response: It could be due to disordered solvent molecules.**

```
PLAT260_ALERT_2_C Large Average Ueq of Residue Including       O21       0.157 Check
```

**Author Response: It could be due to disordered solvent molecules.**

```
PLAT260_ALERT_2_C Large Average Ueq of Residue Including       O22       0.196 Check
```

**Author Response: It could be due to disordered solvent molecules.**

```
PLAT906_ALERT_3_C Large K Value in the Analysis of Variance ......      10.944 Check
PLAT906_ALERT_3_C Large K Value in the Analysis of Variance ......       2.561 Check
PLAT911_ALERT_3_C Missing FCF Refl Between Thmin & STh/L=    0.440         156 Report
PLAT921_ALERT_1_C R1  in the CIF and FCF Differ by ..............     0.0025 Check
PLAT923_ALERT_1_C S    Values in the CIF and FCF Differ by .......      0.034 Check
PLAT975_ALERT_2_C Check Calcd Resid. Dens.  0.87Ang From C131    .       0.55 eA-3
PLAT975_ALERT_2_C Check Calcd Resid. Dens.  0.92Ang From C116    .       0.41 eA-3
PLAT976_ALERT_2_C Check Calcd Resid. Dens.  0.88Ang From C118    .      -0.45 eA-3
PLAT976_ALERT_2_C Check Calcd Resid. Dens.  0.99Ang From O22     .      -0.42 eA-3
```

---

### Alert level G

```
PLAT002_ALERT_2_G Number of Distance or Angle Restraints on AtSite         33 Note
PLAT003_ALERT_2_G Number of Uiso or Uij Restrained non-H Atoms ...        173 Report
PLAT072_ALERT_2_G SHELXL First  Parameter in WGHT  Unusually Large       0.20 Report
PLAT172_ALERT_4_G The CIF-Embedded .res File Contains DFIX Records         15 Report
PLAT178_ALERT_4_G The CIF-Embedded .res File Contains SIMU Records          1 Report
PLAT187_ALERT_4_G The CIF-Embedded .res File Contains RIGU Records          1 Report
PLAT333_ALERT_2_G Large Aver C6-Ring C-C Dist C2       -C19      .       1.42 Ang.
PLAT344_ALERT_2_G Unusual sp?      Angle Range in Solvent/Ion for         C91 Check
PLAT344_ALERT_2_G Unusual sp?      Angle Range in Solvent/Ion for        C107 Check
PLAT344_ALERT_2_G Unusual sp?      Angle Range in Solvent/Ion for        C109 Check
PLAT344_ALERT_2_G Unusual sp?      Angle Range in Solvent/Ion for        C111 Check
PLAT344_ALERT_2_G Unusual sp?      Angle Range in Solvent/Ion for        C108 Check
PLAT344_ALERT_2_G Unusual sp?      Angle Range in Solvent/Ion for        C113 Check
PLAT344_ALERT_2_G Unusual sp?      Angle Range in Solvent/Ion for        C116 Check
PLAT344_ALERT_2_G Unusual sp?      Angle Range in Solvent/Ion for        C117 Check
PLAT344_ALERT_2_G Unusual sp?      Angle Range in Solvent/Ion for        C124 Check
PLAT344_ALERT_2_G Unusual sp?      Angle Range in Solvent/Ion for        C126 Check
PLAT344_ALERT_2_G Unusual sp?      Angle Range in Solvent/Ion for        C129 Check
PLAT344_ALERT_2_G Unusual sp?      Angle Range in Solvent/Ion for        C130 Check
PLAT344_ALERT_2_G Unusual sp?      Angle Range in Solvent/Ion for        C110 Check
PLAT344_ALERT_2_G Unusual sp?      Angle Range in Solvent/Ion for        C112 Check
PLAT344_ALERT_2_G Unusual sp?      Angle Range in Solvent/Ion for        C119 Check
PLAT344_ALERT_2_G Unusual sp?      Angle Range in Solvent/Ion for        C125 Check
PLAT344_ALERT_2_G Unusual sp?      Angle Range in Solvent/Ion for        C131 Check
PLAT344_ALERT_2_G Unusual sp?      Angle Range in Solvent/Ion for        C132 Check
PLAT344_ALERT_2_G Unusual sp?      Angle Range in Solvent/Ion for        C134 Check
PLAT344_ALERT_2_G Unusual sp?      Angle Range in Solvent/Ion for        C135 Check
PLAT344_ALERT_2_G Unusual sp?      Angle Range in Solvent/Ion for        C114 Check
PLAT344_ALERT_2_G Unusual sp?      Angle Range in Solvent/Ion for        C121 Check
PLAT344_ALERT_2_G Unusual sp?      Angle Range in Solvent/Ion for        C128 Check
PLAT344_ALERT_2_G Unusual sp?      Angle Range in Solvent/Ion for        C133 Check
PLAT344_ALERT_2_G Unusual sp?      Angle Range in Solvent/Ion for        C118 Check
PLAT344_ALERT_2_G Unusual sp?      Angle Range in Solvent/Ion for        C141 Check
PLAT344_ALERT_2_G Unusual sp?      Angle Range in Solvent/Ion for        C142 Check
PLAT344_ALERT_2_G Unusual sp?      Angle Range in Solvent/Ion for        C144 Check
PLAT344_ALERT_2_G Unusual sp?      Angle Range in Solvent/Ion for        C115 Check
PLAT344_ALERT_2_G Unusual sp?      Angle Range in Solvent/Ion for        C123 Check
PLAT344_ALERT_2_G Unusual sp?      Angle Range in Solvent/Ion for        C127 Check
PLAT344_ALERT_2_G Unusual sp?      Angle Range in Solvent/Ion for        C136 Check
PLAT344_ALERT_2_G Unusual sp?      Angle Range in Solvent/Ion for        C105 Check
PLAT344_ALERT_2_G Unusual sp?      Angle Range in Solvent/Ion for        C120 Check
PLAT344_ALERT_2_G Unusual sp?      Angle Range in Solvent/Ion for        C122 Check
PLAT344_ALERT_2_G Unusual sp?      Angle Range in Solvent/Ion for        C143 Check
PLAT344_ALERT_2_G Unusual sp?      Angle Range in Solvent/Ion for        C137 Check
PLAT344_ALERT_2_G Unusual sp?      Angle Range in Solvent/Ion for        C138 Check
PLAT344_ALERT_2_G Unusual sp?      Angle Range in Solvent/Ion for        C139 Check
PLAT344_ALERT_2_G Unusual sp?      Angle Range in Solvent/Ion for        C140 Check
PLAT344_ALERT_2_G Unusual sp?      Angle Range in Solvent/Ion for        C145 Check
PLAT344_ALERT_2_G Unusual sp?      Angle Range in Solvent/Ion for        C146 Check
PLAT344_ALERT_2_G Unusual sp?      Angle Range in Solvent/Ion for        C147 Check
PLAT344_ALERT_2_G Unusual sp?      Angle Range in Solvent/Ion for        C148 Check
PLAT367_ALERT_2_G Long?  C(sp?)-C(sp?) Bond  C91      - C109     .       1.51 Ang.
PLAT367_ALERT_2_G Long?  C(sp?)-C(sp?) Bond  C108     - C116     .       1.56 Ang.
PLAT367_ALERT_2_G Long?  C(sp?)-C(sp?) Bond  C129     - C130     .       1.54 Ang.
PLAT367_ALERT_2_G Long?  C(sp?)-C(sp?) Bond  C112     - C119     .       1.52 Ang.
PLAT367_ALERT_2_G Long?  C(sp?)-C(sp?) Bond  C131     - C132     .       1.52 Ang.
```

```
PLAT367_ALERT_2_G Long?  C(sp?)-C(sp?) Bond  C131     - C134     .       1.56 Ang.
PLAT367_ALERT_2_G Long?  C(sp?)-C(sp?) Bond  C132     - C135     .       1.53 Ang.
PLAT367_ALERT_2_G Long?  C(sp?)-C(sp?) Bond  C114     - C128     .       1.52 Ang.
PLAT367_ALERT_2_G Long?  C(sp?)-C(sp?) Bond  C114     - C133     .       1.51 Ang.
PLAT367_ALERT_2_G Long?  C(sp?)-C(sp?) Bond  C121     - C133     .       1.54 Ang.
PLAT367_ALERT_2_G Long?  C(sp?)-C(sp?) Bond  C118     - C141     .       1.53 Ang.
PLAT367_ALERT_2_G Long?  C(sp?)-C(sp?) Bond  C118     - C144     .       1.53 Ang.
PLAT367_ALERT_2_G Long?  C(sp?)-C(sp?) Bond  C127     - C136     .       1.54 Ang.
PLAT367_ALERT_2_G Long?  C(sp?)-C(sp?) Bond  C120     - C122     .       1.52 Ang.
PLAT367_ALERT_2_G Long?  C(sp?)-C(sp?) Bond  C139     - C140     .       1.51 Ang.
PLAT367_ALERT_2_G Long?  C(sp?)-C(sp?) Bond  C145     - C147     .       1.53 Ang.
PLAT367_ALERT_2_G Long?  C(sp?)-C(sp?) Bond  C146     - C148     .       1.52 Ang.
PLAT398_ALERT_2_G Deviating  C-O-C   Angle From 120 for O15      .      107.4 Degree
PLAT398_ALERT_2_G Deviating  C-O-C   Angle From 120 for O16      .      108.7 Degree
PLAT398_ALERT_2_G Deviating  C-O-C   Angle From 120 for O17      .      105.5 Degree
PLAT398_ALERT_2_G Deviating  C-O-C   Angle From 120 for O18      .      107.7 Degree
PLAT398_ALERT_2_G Deviating  C-O-C   Angle From 120 for O19      .      102.8 Degree
PLAT398_ALERT_2_G Deviating  C-O-C   Angle From 120 for O21      .      109.6 Degree
PLAT398_ALERT_2_G Deviating  C-O-C   Angle From 120 for O23      .       96.1 Degree
PLAT432_ALERT_2_G Short Inter X...Y Contact  C122     ..C142     .       3.18 Ang.
                                                1-x,-y,-z  =      2_655 Check
PLAT432_ALERT_2_G Short Inter X...Y Contact  C141     ..C141     .       2.29 Ang.
                                                1-x,-y,-z  =      2_655 Check
PLAT790_ALERT_4_G Centre of Gravity not Within Unit Cell: Resd.  #          4 Note
              C4 O
PLAT790_ALERT_4_G Centre of Gravity not Within Unit Cell: Resd.  #          9 Note
              C4 O
PLAT802_ALERT_4_G CIF Input Record(s) with more than 80 Characters          1 Info
PLAT860_ALERT_3_G Number of Least-Squares Restraints ............        2515 Note
PLAT909_ALERT_3_G Percentage of I>2sig(I) Data at Theta(Max) Still        36% Note
PLAT978_ALERT_2_G Number C-C Bonds with Positive Residual Density.          0 Info
```

---

2 **ALERT level A** = Most likely a serious problem - resolve or explain
7 **ALERT level B** = A potentially serious problem, consider carefully
49 **ALERT level C** = Check. Ensure it is not caused by an omission or oversight
83 **ALERT level G** = General information/check it is not something unexpected

5 ALERT type 1 CIF construction/syntax error, inconsistent or missing data
92 ALERT type 2 Indicator that the structure model may be wrong or deficient
12 ALERT type 3 Indicator that the structure quality may be low
32 ALERT type 4 Improvement, methodology, query or suggestion
0 ALERT type 5 Informative message, check

---

It is advisable to attempt to resolve as many as possible of the alerts in all categories. Often the minor alerts point to easily fixed oversights, errors and omissions in your CIF or refinement strategy, so attention to these fine details can be worthwhile. In order to resolve some of the more serious problems it may be necessary to carry out additional measurements or structure refinements. However, the purpose of your study may justify the reported deviations and the more serious of these should normally be commented upon in the discussion or experimental section of a paper or in the "special_details" fields of the CIF. checkCIF was carefully designed to identify outliers and unusual parameters, but every test has its limitations and alerts that are not important in a particular case may appear. Conversely, the absence of alerts does not guarantee there are no aspects of the results needing attention. It is up to the individual to critically assess their own results and, if necessary, seek expert advice.

**Publication of your CIF in IUCr journals**

A basic structural check has been run on your CIF. These basic checks will be run on all CIFs submitted for publication in IUCr journals (*Acta Crystallographica*, *Journal of Applied Crystallography*, *Journal of Synchrotron Radiation*); however, if you intend to submit to *Acta Crystallographica Section C* or *E* or *IUCrData*, you should make sure that full publication checks are run on the final version of your CIF prior to submission.

**Publication of your CIF in other journals**

Please refer to the *Notes for Authors* of the relevant journal for any special instructions relating to CIF submission.

---

**PLATON version of 28/11/2022; check.def file version of 28/11/2022**

**Datablock mo_2012074967_0m** - ellipsoid plot

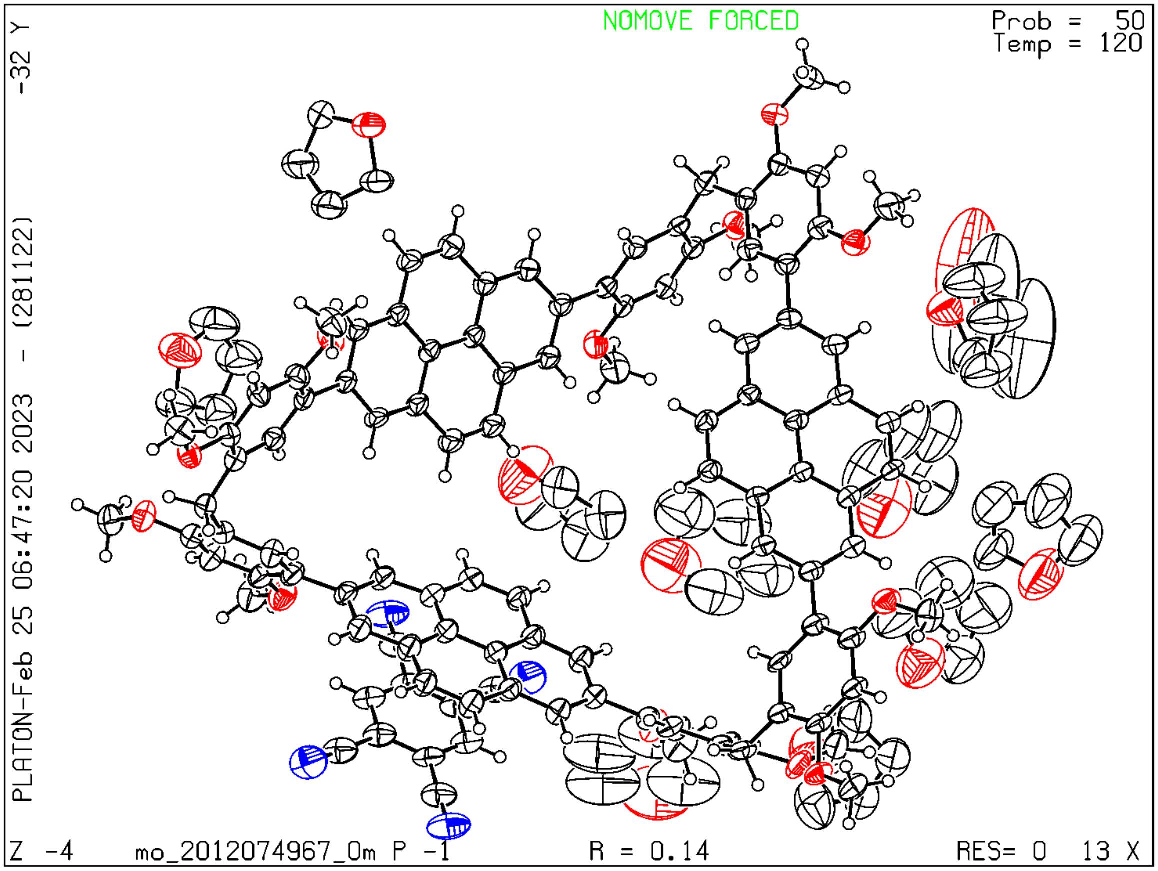

Supplement: Supplementary file 5 — Source Data [file MCC-1-checkcif.pdf › Single-crystal structures/MCC-1-checkcif.pdf]
