# Supplementary material for: Structurally diverse macrocycle co-crystals for solid-state luminescence modulation
Source: Nat Commun. 2024 Mar 21;15:2535. doi: 10.1038/s41467-024-46788-6 (PMC10957888; doi:10.1038/s41467-024-46788-6)

# checkCIF/PLATON report

Structure factors have been supplied for datablock(s) zzy8_0m_sq

THIS REPORT IS FOR GUIDANCE ONLY. IF USED AS PART OF A REVIEW PROCEDURE FOR PUBLICATION, IT SHOULD NOT REPLACE THE EXPERTISE OF AN EXPERIENCED CRYSTALLOGRAPHIC REFEREE.

No syntax errors found. CIF dictionary Interpreting this report

# Datablock: zzy8_0m_sq

---

Bond precision: C-C = 0.0152 A Wavelength=0.71073

| | | | |
|---|---|---|---|
| Cell: | a=19.465(2) | b=19.978(2) | c=20.219(2) |
| | alpha=93.593(3) | beta=116.099(3) | gamma=99.007(3) |
| Temperature: | 150 K | | |

| | Calculated | Reported |
|---|---|---|
| Volume | 6896.7(12) | 6896.8(14) |
| Space group | P -1 | P -1 |
| Hall group | -P 1 | -P 1 |
| Moiety formula | C99 H78 O12, C10 H2 N4, C4 H8 O2 [+ solvent] | 2(C99 H78 O12), 2(C10 H2 N4), 2(C4 H8 O2) |
| Sum formula | C113 H88 N4 O14 [+ solvent] | C226 H176 N8 O28 |
| Mr | 1725.88 | 3451.74 |
| Dx,g cm-3 | 0.831 | 0.831 |
| Z | 2 | 1 |
| Mu (mm-1) | 0.055 | 0.055 |
| F000 | 1812.0 | 1812.0 |
| F000' | 1812.84 | |
| h,k,lmax | 22,23,23 | 22,23,23 |
| Nref | 23508 | 23402 |
| Tmin,Tmax | 0.997,0.998 | 0.638,0.745 |
| Tmin' | 0.996 | |

Correction method= # Reported T Limits: Tmin=0.638 Tmax=0.745
AbsCorr = NONE

Data completeness= 0.995 Theta(max)= 24.713

R(reflections)= 0.1283( 6917) wR2(reflections)= 0.4206( 23402)

S = 1.008 Npar= 1193

The following ALERTS were generated. Each ALERT has the format
**test-name_ALERT_alert-type_alert-level.**
Click on the hyperlinks for more details of the test.

### Alert level B

PLAT026_ALERT_3_B Ratio Observed / Unique Reflections (too) Low .. 30% Check

**Author Response: This alert is due to the small size of the crystal and bad diffraction**

PLAT084_ALERT_3_B High wR2 Value (i.e. > 0.25) .................. 0.42 Report

**Author Response: We made several attempts to obtain better quality data for this structure, however, due to poor crystal quality, the wR2 value is high.**

PLAT230_ALERT_2_B Hirshfeld Test Diff for O1 --C031 . 8.5 s.u.

**Author Response: Caused by disorder of Pe[3].**

PLAT230_ALERT_2_B Hirshfeld Test Diff for C00X --C01H . 7.1 s.u.

**Author Response: Caused by disorder of Pe[3].**

PLAT230_ALERT_2_B Hirshfeld Test Diff for C01M --C01Z . 7.4 s.u.

**Author Response: Caused by disorder of Pe[3].**

PLAT230_ALERT_2_B Hirshfeld Test Diff for C01P --C02H . 9.1 s.u.

**Author Response: Caused by disorder of Pe[3].**

PLAT230_ALERT_2_B Hirshfeld Test Diff for C01X --C035 . 8.2 s.u.

**Author Response: Caused by disorder of Pe[3].**

PLAT230_ALERT_2_B Hirshfeld Test Diff for C01Z --C02L . 8.6 s.u.

**Author Response: Caused by disorder of Pe[3].**

PLAT230_ALERT_2_B Hirshfeld Test Diff for C020 --C029 . 10.6 s.u.

**Author Response: Caused by disorder of Pe[3].**

```
PLAT230_ALERT_2_B Hirshfeld Test Diff for    C02L     --C03J     .       11.4 s.u.
```

**Author Response: Caused by disorder of Pe[3].**

```
PLAT230_ALERT_2_B Hirshfeld Test Diff for    C02S     --C031     .        7.9 s.u.
```

**Author Response: Caused by disorder of Pe[3].**

```
PLAT230_ALERT_2_B Hirshfeld Test Diff for    C035     --C038     .        7.4 s.u.
```

**Author Response: Caused by disorder of Pe[3].**

```
PLAT234_ALERT_4_B Large Hirshfeld Difference O3       --C03S     .       0.27 Ang.
```

**Author Response: Caused by disorder of Pe[3].**

```
PLAT241_ALERT_2_B High   'MainMol' Ueq as Compared to Neighbors of       C03J Check
```

**Author Response: Though we tried our best, the final data is not high quality due to the high porosity, causing high Ueq.**

```
PLAT340_ALERT_3_B Low Bond Precision on  C-C Bonds ..............    0.01524 Ang.
```

**Author Response: This is attributed to disorder present in the structure.**

---

### Alert level C

```
RINTA01_ALERT_3_C  The value of Rint is greater than 0.12
            Rint given   0.135
THETM01_ALERT_3_C  The value of sine(theta_max)/wavelength is less than 0.590
            Calculated sin(theta_max)/wavelength =    0.5882
PLAT020_ALERT_3_C The Value of Rint is Greater Than 0.12 ........      0.135 Report
PLAT082_ALERT_2_C High R1 Value ..................................      0.13 Report
PLAT213_ALERT_2_C Atom C026            has ADP max/min Ratio .....       3.3 prolat
PLAT213_ALERT_2_C Atom C03K            has ADP max/min Ratio .....       3.5 prolat
PLAT220_ALERT_2_C NonSolvent   Resd 1  C   Ueq(max)/Ueq(min) Range       3.4 Ratio
PLAT230_ALERT_2_C Hirshfeld Test Diff for    O2       --C02T     .        5.4 s.u.
```

**Author Response: Caused by disorder of Pe[3].**

```
PLAT230_ALERT_2_C Hirshfeld Test Diff for    C00C     --C00Q     .        6.3 s.u.
```

**Author Response: Caused by disorder of Pe[3].**

```
PLAT230_ALERT_2_C Hirshfeld Test Diff for    C00U     --C01R     .         5.4 s.u.
```

**Author Response: Caused by disorder of Pe[3].**

```
PLAT230_ALERT_2_C Hirshfeld Test Diff for    C018     --C01O     .         5.5 s.u.
```

**Author Response: Caused by disorder of Pe[3].**

```
PLAT230_ALERT_2_C Hirshfeld Test Diff for    C01N     --C02K     .         5.5 s.u.
```

**Author Response: Caused by disorder of Pe[3].**

```
PLAT230_ALERT_2_C Hirshfeld Test Diff for    C02S     --C02T     .         6.3 s.u.
```

**Author Response: Caused by disorder of Pe[3].**

```
PLAT234_ALERT_4_C Large Hirshfeld Difference O1       --C1       .        0.18 Ang.
```

**Author Response: Caused by disorder of Pe[3].**

```
PLAT234_ALERT_4_C Large Hirshfeld Difference O3       --C02Q     .        0.17 Ang.
```

**Author Response: Caused by disorder of Pe[3].**

```
PLAT234_ALERT_4_C Large Hirshfeld Difference O4       --C034     .        0.16 Ang.
```

**Author Response: Caused by disorder of Pe[3].**

```
PLAT234_ALERT_4_C Large Hirshfeld Difference O5       --C01U     .        0.18 Ang.
```

**Author Response: Caused by disorder of Pe[3].**

```
PLAT234_ALERT_4_C Large Hirshfeld Difference O6       --C03A     .        0.18 Ang.
```

**Author Response: Caused by disorder of Pe[3].**

```
PLAT234_ALERT_4_C Large Hirshfeld Difference O8       --C03E     .        0.20 Ang.
```

**Author Response: Caused by disorder of Pe[3].**

PLAT234_ALERT_4_C Large Hirshfeld Difference O11 --C03C . 0.16 Ang.

**Author Response: Caused by disorder of Pe[3].**

PLAT234_ALERT_4_C Large Hirshfeld Difference O12 --C030 . 0.16 Ang.

**Author Response: Caused by disorder of Pe[3].**

PLAT234_ALERT_4_C Large Hirshfeld Difference O13 --C038 . 0.21 Ang.

**Author Response: Caused by disorder of Pe[3].**

PLAT234_ALERT_4_C Large Hirshfeld Difference O14 --C03D . 0.20 Ang.

**Author Response: Caused by disorder of Pe[3].**

PLAT234_ALERT_4_C Large Hirshfeld Difference C00H --C018 . 0.18 Ang.

**Author Response: Caused by disorder of Pe[3].**

PLAT234_ALERT_4_C Large Hirshfeld Difference C00H --C02K . 0.19 Ang.

**Author Response: Caused by disorder of Pe[3].**

PLAT234_ALERT_4_C Large Hirshfeld Difference C00J --C00N . 0.17 Ang.

**Author Response: Caused by disorder of Pe[3].**

PLAT234_ALERT_4_C Large Hirshfeld Difference C00K --C022 . 0.19 Ang.

**Author Response: Caused by disorder of Pe[3].**

PLAT234_ALERT_4_C Large Hirshfeld Difference C00N --C00S . 0.20 Ang.

**Author Response: Caused by disorder of Pe[3].**

PLAT234_ALERT_4_C Large Hirshfeld Difference C00O --C00R . 0.16 Ang.

**Author Response: Caused by disorder of Pe[3].**

PLAT234_ALERT_4_C Large Hirshfeld Difference C00P --C019 . 0.19 Ang.

**Author Response: Caused by disorder of Pe[3].**

PLAT234_ALERT_4_C Large Hirshfeld Difference C00T --C00X . 0.17 Ang.

**Author Response: Caused by disorder of Pe[3].**

PLAT234_ALERT_4_C Large Hirshfeld Difference C00U --C01J . 0.17 Ang.

**Author Response: Caused by disorder of Pe[3].**

PLAT234_ALERT_4_C Large Hirshfeld Difference C00U --C026 . 0.21 Ang.

**Author Response: Caused by disorder of Pe[3].**

PLAT234_ALERT_4_C Large Hirshfeld Difference C00Y --C02I . 0.19 Ang.

**Author Response: Caused by disorder of Pe[3].**

PLAT234_ALERT_4_C Large Hirshfeld Difference C00Z --C01H . 0.19 Ang.

**Author Response: Caused by disorder of Pe[3].**

PLAT234_ALERT_4_C Large Hirshfeld Difference C012 --C025 . 0.18 Ang.

**Author Response: Caused by disorder of Pe[3].**

PLAT234_ALERT_4_C Large Hirshfeld Difference C015 --C02M . 0.16 Ang.

**Author Response: Caused by disorder of Pe[3].**

PLAT234_ALERT_4_C Large Hirshfeld Difference C017 --C02V . 0.20 Ang.

**Author Response: Caused by disorder of Pe[3].**

PLAT234_ALERT_4_C Large Hirshfeld Difference C01B --C01J . 0.20 Ang.

**Author Response: Caused by disorder of Pe[3].**

PLAT234_ALERT_4_C Large Hirshfeld Difference C01F --C01O . 0.19 Ang.

**Author Response: Caused by disorder of Pe[3].**

PLAT234_ALERT_4_C Large Hirshfeld Difference C01G --C02X . 0.21 Ang.

**Author Response: Caused by disorder of Pe[3].**

PLAT234_ALERT_4_C Large Hirshfeld Difference C01H --C01I . 0.18 Ang.

**Author Response: Caused by disorder of Pe[3].**

PLAT234_ALERT_4_C Large Hirshfeld Difference C01N --C02B . 0.16 Ang.

**Author Response: Caused by disorder of Pe[3].**

PLAT234_ALERT_4_C Large Hirshfeld Difference C01Q --C023 . 0.18 Ang.

**Author Response: Caused by disorder of Pe[3].**

PLAT234_ALERT_4_C Large Hirshfeld Difference C01Q --C02N . 0.16 Ang.

**Author Response: Caused by disorder of Pe[3].**

PLAT234_ALERT_4_C Large Hirshfeld Difference C01S --C02E . 0.20 Ang.

**Author Response: Caused by disorder of Pe[3].**

PLAT234_ALERT_4_C Large Hirshfeld Difference C01W --C02G . 0.16 Ang.

**Author Response: Caused by disorder of Pe[3].**

PLAT234_ALERT_4_C Large Hirshfeld Difference C01X --C02H . 0.19 Ang.

**Author Response: Caused by disorder of Pe[3].**

PLAT234_ALERT_4_C Large Hirshfeld Difference C020 --C026 . 0.22 Ang.

**Author Response: Caused by disorder of Pe[3].**

PLAT234_ALERT_4_C Large Hirshfeld Difference C028 --C039 . 0.21 Ang.

**Author Response: Caused by disorder of Pe[3].**

PLAT234_ALERT_4_C Large Hirshfeld Difference C02A --C02P . 0.21 Ang.

**Author Response: Caused by disorder of Pe[3].**

PLAT234_ALERT_4_C Large Hirshfeld Difference C02O --C02Q . 0.19 Ang.

**Author Response: Caused by disorder of Pe[3].**

PLAT234_ALERT_4_C Large Hirshfeld Difference C033 --C036 . 0.20 Ang.

**Author Response: Caused by disorder of Pe[3].**

PLAT234_ALERT_4_C Large Hirshfeld Difference N1 --C02U . 0.23 Ang.

**Author Response: Caused by disorder of Pe[3].**

PLAT234_ALERT_4_C Large Hirshfeld Difference C009 --C00F . 0.17 Ang.

**Author Response: Caused by disorder of Pe[3].**

PLAT234_ALERT_4_C Large Hirshfeld Difference C009 --C00V . 0.21 Ang.

**Author Response: Caused by disorder of Pe[3].**

PLAT234_ALERT_4_C Large Hirshfeld Difference C00F --C02R . 0.18 Ang.

**Author Response: Caused by disorder of Pe[3].**

PLAT234_ALERT_4_C Large Hirshfeld Difference C00D --C021 . 0.16 Ang.

**Author Response: Caused by disorder of Pe[3].**

PLAT241_ALERT_2_C High 'MainMol' Ueq as Compared to Neighbors of O14 Check

**Author Response: Though we tried our best, the final data is not high quality due to the high porosity, causing high Ueq.**

```
PLAT241_ALERT_2_C High   'MainMol' Ueq as Compared to Neighbors of       C023 Check
```

**Author Response: Though we tried our best, the final data is not high quality due to the high porosity, causing high Ueq.**

```
PLAT241_ALERT_2_C High   'MainMol' Ueq as Compared to Neighbors of       C025 Check
```

**Author Response: Though we tried our best, the final data is not high quality due to the high porosity, causing high Ueq.**

```
PLAT241_ALERT_2_C High   'MainMol' Ueq as Compared to Neighbors of       C029 Check
```

**Author Response: Though we tried our best, the final data is not high quality due to the high porosity, causing high Ueq.**

```
PLAT241_ALERT_2_C High   'MainMol' Ueq as Compared to Neighbors of       C02K Check
```

**Author Response: Though we tried our best, the final data is not high quality due to the high porosity, causing high Ueq.**

```
PLAT241_ALERT_2_C High   'MainMol' Ueq as Compared to Neighbors of       C02Y Check
```

**Author Response: Though we tried our best, the final data is not high quality due to the high porosity, causing high Ueq.**

```
PLAT241_ALERT_2_C High   'MainMol' Ueq as Compared to Neighbors of       C035 Check
```

**Author Response: Though we tried our best, the final data is not high quality due to the high porosity, causing high Ueq.**

```
PLAT242_ALERT_2_C Low    'MainMol' Ueq as Compared to Neighbors of        O12 Check
PLAT242_ALERT_2_C Low    'MainMol' Ueq as Compared to Neighbors of       C01X Check
PLAT242_ALERT_2_C Low    'MainMol' Ueq as Compared to Neighbors of       C020 Check
PLAT242_ALERT_2_C Low    'MainMol' Ueq as Compared to Neighbors of       C02L Check
PLAT242_ALERT_2_C Low    'MainMol' Ueq as Compared to Neighbors of       C036 Check
PLAT242_ALERT_2_C Low    'MainMol' Ueq as Compared to Neighbors of       C009 Check
PLAT243_ALERT_4_C High   'Solvent' Ueq as Compared to Neighbors of       C03V Check
PLAT244_ALERT_4_C Low    'Solvent' Ueq as Compared to Neighbors of         O9 Check
PLAT244_ALERT_4_C Low    'Solvent' Ueq as Compared to Neighbors of        O10 Check
PLAT250_ALERT_2_C Large U3/U1 Ratio for Average U(i,j) Tensor ....        2.1 Note
PLAT250_ALERT_2_C Large U3/U1 Ratio for Average U(i,j) Tensor ....        3.8 Note
PLAT250_ALERT_2_C Large U3/U1 Ratio for Average U(i,j) Tensor ....        2.2 Note
PLAT260_ALERT_2_C Large Average Ueq of Residue Including        O1      0.101 Check
PLAT260_ALERT_2_C Large Average Ueq of Residue Including        N1      0.111 Check
PLAT260_ALERT_2_C Large Average Ueq of Residue Including        O9      0.190 Check
PLAT334_ALERT_2_C Small <C-C> Benzene Dist.   C010     -C01T     .       1.37 Ang.
PLAT334_ALERT_2_C Small <C-C> Benzene Dist.   C01M     -C02T     .       1.37 Ang.
PLAT334_ALERT_2_C Small <C-C> Benzene Dist.   C01S     -C02O     .       1.37 Ang.
```

PLAT363_ALERT_2_C Long C(sp3)-C(sp2) Bond C036 - C03J . 1.63 Ang.
PLAT412_ALERT_2_C Short Intra XH3 .. XHn H02M ..H03U . 1.85 Ang.
x,y,z = 1_555 Check
PLAT905_ALERT_3_C Negative K value in the Analysis of Variance ... -26.874 Report
PLAT905_ALERT_3_C Negative K value in the Analysis of Variance ... -1.734 Report
PLAT910_ALERT_3_C Missing # of FCF Reflection(s) Below Theta(Min). 10 Note
PLAT911_ALERT_3_C Missing FCF Refl Between Thmin & STh/L= 0.588 96 Report
PLAT913_ALERT_3_C Missing # of Very Strong Reflections in FCF .... 5 Note
PLAT918_ALERT_3_C Reflection(s) with I(obs) much Smaller I(calc) . 5 Check

---

## Alert level G

PLAT003_ALERT_2_G Number of Uiso or Uij Restrained non-H Atoms ... 1 Report
PLAT045_ALERT_1_G Calculated and Reported Z Differ by a Factor ... 2 Check
PLAT072_ALERT_2_G SHELXL First Parameter in WGHT Unusually Large 0.20 Report
PLAT152_ALERT_1_G The Supplied and Calc. Volume s.u. Differ by ... -2 Units
PLAT154_ALERT_1_G The s.u.'s on the Cell Angles are Equal ..(Note) 0.003 Degree
PLAT186_ALERT_4_G The CIF-Embedded .res File Contains ISOR Records 1 Report
PLAT230_ALERT_2_G Hirshfeld Test Diff for N4 --C021 . 8.3 s.u.

**Author Response: Caused by disorder of Pe[3].**

PLAT333_ALERT_2_G Large Aver C6-Ring C-C Dist C00E -C00S . 1.43 Ang.
PLAT333_ALERT_2_G Large Aver C6-Ring C-C Dist C018 -C01Q . 1.42 Ang.
PLAT335_ALERT_2_G Check Large C6 Ring C-C Range C00C -C01J 0.16 Ang.
PLAT335_ALERT_2_G Check Large C6 Ring C-C Range C00E -C013 0.16 Ang.
PLAT335_ALERT_2_G Check Large C6 Ring C-C Range C00P -C025 0.17 Ang.
PLAT335_ALERT_2_G Check Large C6 Ring C-C Range C018 -C01Q 0.20 Ang.
PLAT335_ALERT_2_G Check Large C6 Ring C-C Range C01M -C02T 0.22 Ang.
PLAT398_ALERT_2_G Deviating C-O-C Angle From 120 for O9 . 107.4 Degree
PLAT432_ALERT_2_G Short Inter X...Y Contact C020 ..C03K . 3.20 Ang.
1+x,y,z = 1_655 Check
PLAT432_ALERT_2_G Short Inter X...Y Contact C026 ..C03K . 3.16 Ang.
1+x,y,z = 1_655 Check
PLAT606_ALERT_4_G Solvent Accessible VOID(S) in Structure ........ ! Info
PLAT720_ALERT_4_G Number of Unusual/Non-Standard Labels .......... 192 Note
PLAT802_ALERT_4_G CIF Input Record(s) with more than 80 Characters 1 Info
PLAT860_ALERT_3_G Number of Least-Squares Restraints ............ 6 Note
PLAT869_ALERT_4_G ALERTS Related to the Use of SQUEEZE Suppressed ! Info
PLAT933_ALERT_2_G Number of HKL-OMIT Records in Embedded .res File 21 Note
PLAT941_ALERT_3_G Average HKL Measurement Multiplicity .......... 3.0 Low
PLAT978_ALERT_2_G Number C-C Bonds with Positive Residual Density. 0 Info

---

0 **ALERT level A** = Most likely a serious problem - resolve or explain
15 **ALERT level B** = A potentially serious problem, consider carefully
91 **ALERT level C** = Check. Ensure it is not caused by an omission or oversight
25 **ALERT level G** = General information/check it is not something unexpected

3 ALERT type 1 CIF construction/syntax error, inconsistent or missing data
60 ALERT type 2 Indicator that the structure model may be wrong or deficient
14 ALERT type 3 Indicator that the structure quality may be low
54 ALERT type 4 Improvement, methodology, query or suggestion
0 ALERT type 5 Informative message, check

---

---

It is advisable to attempt to resolve as many as possible of the alerts in all categories. Often the minor alerts point to easily fixed oversights, errors and omissions in your CIF or refinement strategy, so attention to these fine details can be worthwhile. In order to resolve some of the more serious problems it may be necessary to carry out additional measurements or structure refinements. However, the purpose of your study may justify the reported deviations and the more serious of these should normally be commented upon in the discussion or experimental section of a paper or in the "special_details" fields of the CIF. checkCIF was carefully designed to identify outliers and unusual parameters, but every test has its limitations and alerts that are not important in a particular case may appear. Conversely, the absence of alerts does not guarantee there are no aspects of the results needing attention. It is up to the individual to critically assess their own results and, if necessary, seek expert advice.

**Publication of your CIF in IUCr journals**

A basic structural check has been run on your CIF. These basic checks will be run on all CIFs submitted for publication in IUCr journals (*Acta Crystallographica*, *Journal of Applied Crystallography*, *Journal of Synchrotron Radiation*); however, if you intend to submit to *Acta Crystallographica Section C* or *E* or *IUCrData*, you should make sure that full publication checks are run on the final version of your CIF prior to submission.

**Publication of your CIF in other journals**

Please refer to the *Notes for Authors* of the relevant journal for any special instructions relating to CIF submission.

---

**PLATON version of 28/11/2022; check.def file version of 28/11/2022**

**Datablock zzy8_0m_sq** - ellipsoid plot

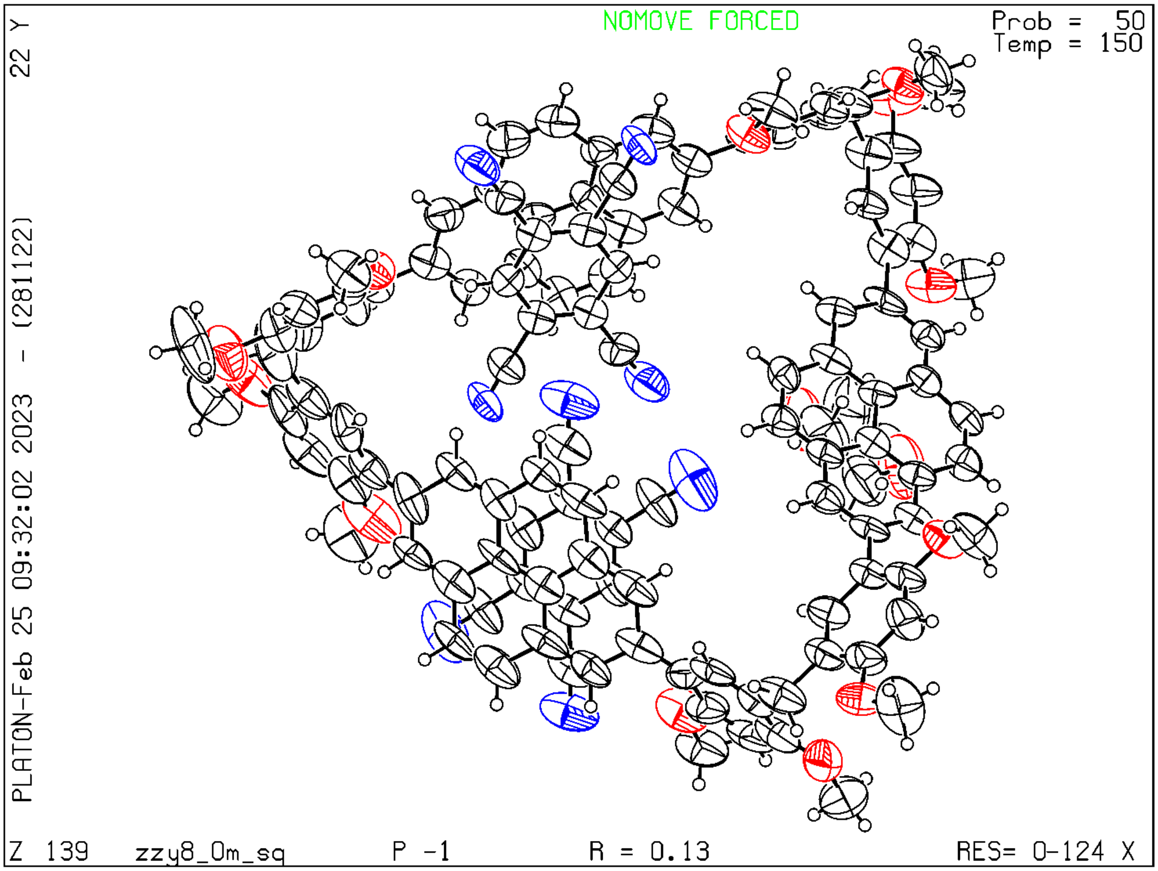

Supplement: Supplementary file 5 — Source Data [file MCC-2-checkcif.pdf › Single-crystal structures/MCC-2-checkcif.pdf]
