# Supplementary material for: Structurally diverse macrocycle co-crystals for solid-state luminescence modulation
Source: Nat Commun. 2024 Mar 21;15:2535. doi: 10.1038/s41467-024-46788-6 (PMC10957888; doi:10.1038/s41467-024-46788-6)

checkCIF/PLATON report

Structure factors have been supplied for datablock(s) 2

THIS REPORT IS FOR GUIDANCE ONLY. IF USED AS PART OF A REVIEW PROCEDURE FOR PUBLICATION, IT SHOULD NOT REPLACE THE EXPERTISE OF AN EXPERIENCED CRYSTALLOGRAPHIC REFEREE.

No syntax errors found. CIF dictionary Interpreting this report

Datablock: 2

Bond precision:	C-C = 0.0042 A		Wavelength=0.71073
Cell:	a=13.9873(8)	b=17.1586(11)	c=25.3581(16)
	alpha=73.354(2)	beta=77.262(2)	gamma=71.157(2)
Temperature:	193 K		

	Calculated	Reported
Volume	5463.2(6)	5463.2(6)
Space group	P -1	P -1
Hall group	-P 1	-P 1
Moiety formula	C99 H78 O12, 2(C2 H6 O S) [+ solvent]	2(C2 H6 O S), C99 H78 O12, 6.5[1DMSO], 1[2.51DMSO]
Sum formula	C103 H90 O14 S2 [+ solvent]	C103 H90 O14 S2
Mr	1615.88	1615.86
Dx,g cm-3	0.982	0.982
Z	2	2
Mu (mm-1)	0.101	0.101
F000	1704.0	1704.0
F000'	1705.28	
h,k,lmax	18,22,32	18,22,32
Nref	25166	24896
Tmin,Tmax	0.976,0.990	0.618,0.746
Tmin'	0.970	

Correction method= # Reported T Limits: Tmin=0.618 Tmax=0.746
AbsCorr = NONE

Data completeness= 0.989 Theta(max)= 27.528

R(reflections)= 0.0829(14841) wR2(reflections)= 0.2842(24896)

S = 1.037 Npar= 1088

The following ALERTS were generated. Each ALERT has the format
test-name_ALERT_alert-type_alert-level.
Click on the hyperlinks for more details of the test.

Alert level A

```
PLAT413_ALERT_2_A Short Inter XH3 .. XHn     H10H     ..H45      .       1.76 Ang.
                                                -1+x,y,z  =      1_455 Check
```

Author Response: This alert is generated because there are disorders of methyl in the solvent

Alert level B

```
PLAT413_ALERT_2_B Short Inter XH3 .. XHn     H10I     ..H51A     .       1.96 Ang.
                                              -1+x,1+y,z  =      1_465 Check
```

Author Response: This alert is generated because there are disorders of methyl in the solvent

Alert level C

```
CRYSC01_ALERT_1_C No recognised colour has been given for crystal colour.
PLAT084_ALERT_3_C High wR2 Value (i.e. > 0.25) ...................       0.28 Report
PLAT244_ALERT_4_C Low    'Solvent' Ueq as Compared to Neighbors of         S2 Check
PLAT260_ALERT_2_C Large Average Ueq of Residue Including        S2      0.102 Check
PLAT340_ALERT_3_C Low Bond Precision on  C-C Bonds ..............    0.00416 Ang.
PLAT413_ALERT_2_C Short Inter XH3 .. XHn     H23      ..H50A     .       2.13 Ang.
                                             2-x,1-y,1-z  =      2_766 Check
```

Author Response: This alert is generated because there are disorders of methyl in the solvent

```
PLAT413_ALERT_2_C Short Inter XH3 .. XHn     H10G     ..H42      .       2.00 Ang.
                                             2-x,1-y,1-z  =      2_766 Check
```

Author Response: This alert is generated because there are disorders of methyl in the solvent

```
PLAT413_ALERT_2_C Short Inter XH3 .. XHn     H10G     ..H44      .       2.14 Ang.
                                             2-x,1-y,1-z  =      2_766 Check
```

Author Response: This alert is generated because there are disorders of methyl in the solvent

```
PLAT905_ALERT_3_C Negative K value in the Analysis of Variance ...     -0.053 Report
PLAT910_ALERT_3_C Missing # of FCF Reflection(s) Below Theta(Min).          9 Note
PLAT911_ALERT_3_C Missing FCF Refl Between Thmin & STh/L=    0.600        141 Report
PLAT913_ALERT_3_C Missing # of Very Strong Reflections in FCF ....         17 Note
PLAT918_ALERT_3_C Reflection(s) with I(obs) much Smaller I(calc) .         11 Check
PLAT977_ALERT_2_C Check Negative Difference Density on H10H      .      -0.37 eA-3
```

Alert level G

```
FORMU01_ALERT_1_G  There is a discrepancy between the atom counts in the
            _chemical_formula_sum and _chemical_formula_moiety. This is
            usually due to the moiety formula being in the wrong format.
            Atom count from _chemical_formula_sum:   C103 H90 O14 S2
            Atom count from _chemical_formula_moiety:C103 H90 D6.5 O20.5 S8.5
PLAT042_ALERT_1_G Calc. and Reported MoietyFormula Strings  Differ     Please Check
PLAT072_ALERT_2_G SHELXL First  Parameter in WGHT  Unusually Large       0.16 Report
PLAT154_ALERT_1_G The s.u.'s on the Cell Angles are Equal ..(Note)      0.002 Degree
PLAT333_ALERT_2_G Large Aver C6-Ring C-C Dist C43      -C49      .       1.42 Ang.
PLAT606_ALERT_4_G Solvent Accessible VOID(S) in Structure ........          ! Info
PLAT868_ALERT_4_G ALERTS Due to the Use of _smtbx_masks Suppressed          ! Info
PLAT912_ALERT_4_G Missing # of FCF Reflections Above STh/L=  0.600        122 Note
PLAT933_ALERT_2_G Number of HKL-OMIT Records in Embedded .res File         17 Note
PLAT941_ALERT_3_G Average HKL Measurement Multiplicity ..........         2.1 Low
PLAT978_ALERT_2_G Number C-C Bonds with Positive Residual Density.          4 Info
```

---

1 **ALERT level A** = Most likely a serious problem - resolve or explain
1 **ALERT level B** = A potentially serious problem, consider carefully
14 **ALERT level C** = Check. Ensure it is not caused by an omission or oversight
11 **ALERT level G** = General information/check it is not something unexpected

4 ALERT type 1 CIF construction/syntax error, inconsistent or missing data
11 ALERT type 2 Indicator that the structure model may be wrong or deficient
8 ALERT type 3 Indicator that the structure quality may be low
4 ALERT type 4 Improvement, methodology, query or suggestion
0 ALERT type 5 Informative message, check

It is advisable to attempt to resolve as many as possible of the alerts in all categories. Often the minor alerts point to easily fixed oversights, errors and omissions in your CIF or refinement strategy, so attention to these fine details can be worthwhile. In order to resolve some of the more serious problems it may be necessary to carry out additional measurements or structure refinements. However, the purpose of your study may justify the reported deviations and the more serious of these should normally be commented upon in the discussion or experimental section of a paper or in the "special_details" fields of the CIF. checkCIF was carefully designed to identify outliers and unusual parameters, but every test has its limitations and alerts that are not important in a particular case may appear. Conversely, the absence of alerts does not guarantee there are no aspects of the results needing attention. It is up to the individual to critically assess their own results and, if necessary, seek expert advice.

Publication of your CIF in IUCr journals

A basic structural check has been run on your CIF. These basic checks will be run on all CIFs submitted for publication in IUCr journals (*Acta Crystallographica*, *Journal of Applied Crystallography*, *Journal of Synchrotron Radiation*); however, if you intend to submit to *Acta Crystallographica Section C* or *E* or *IUCrData*, you should make sure that full publication checks are run on the final version of your CIF prior to submission.

Publication of your CIF in other journals

Please refer to the *Notes for Authors* of the relevant journal for any special instructions relating to CIF submission.

PLATON version of 06/07/2023; check.def file version of 30/06/2023

Datablock 2 - ellipsoid plot

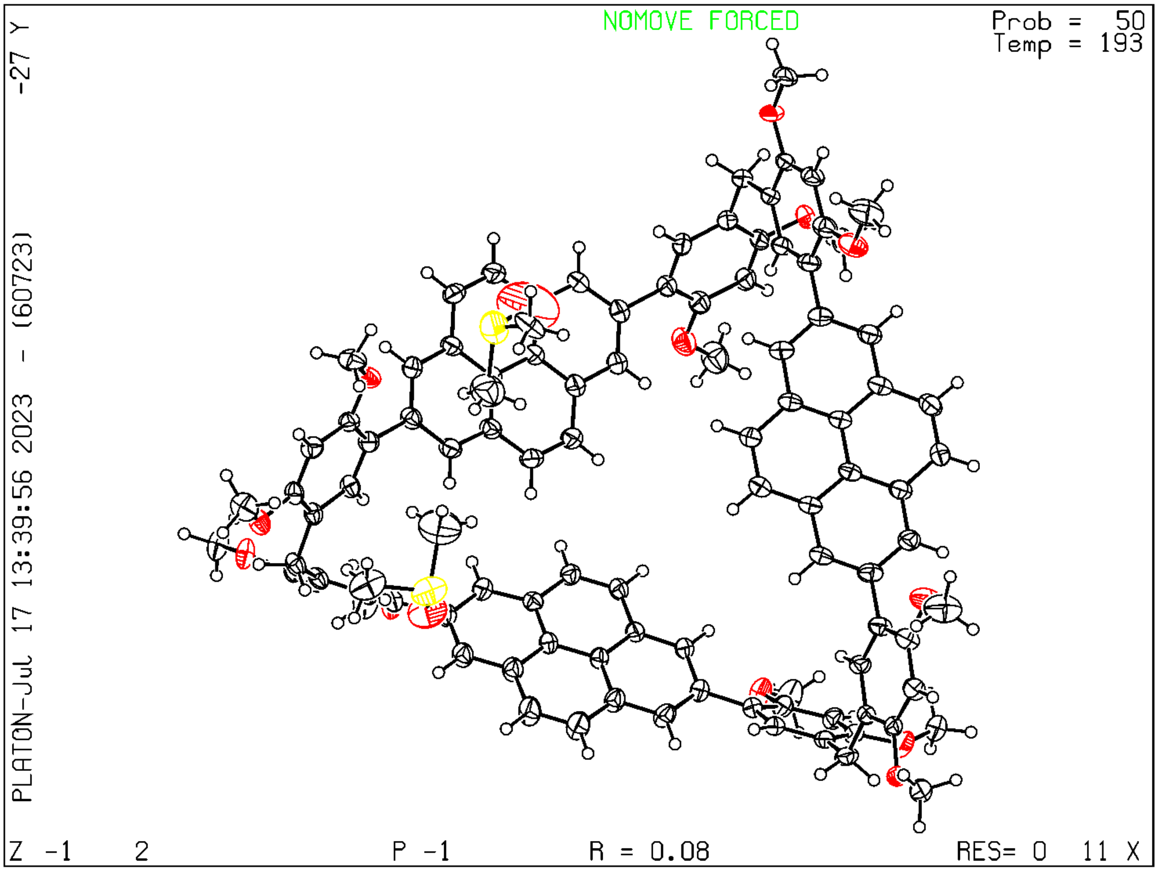

Supplement: Supplementary file 5 — Source Data [file Pe[3]-DMSO-checkcif.pdf › Single-crystal structures/Pe[3]-DMSO-checkcif.pdf]
